# Comparison of the Abiotic Preferences of Macroinvertebrates in Tropical River Basins

**DOI:** 10.1371/journal.pone.0108898

**Published:** 2014-10-03

**Authors:** Gert Everaert, Jan De Neve, Pieter Boets, Luis Dominguez-Granda, Seid Tiku Mereta, Argaw Ambelu, Thu Huong Hoang, Peter L. M. Goethals, Olivier Thas

**Affiliations:** 1 Aquatic Ecology Research Unit, Department Applied Ecology and Environmental Biology, Ghent University, Ghent, Belgium; 2 Environmental Toxicology Research Group, Department Applied Ecology and Environmental Biology, Ghent University, Ghent, Belgium; 3 Department of Mathematical Modelling, Statistics and Bioinformatics, Ghent University, Ghent, Belgium; 4 Department of Chemical and Environmental Sciences, Escuela Superior Politécnica del Litoral (ESPOL), Guayaquil, Ecuador; 5 Department of Environmental Health Science and Technology, Jimma University, Jimma, Ethiopia; 6 School of Environmental Science and Technology, Hanoi University of Science and Technology, Hanoi, Vietnam; 7 National Institute for Applied Statistics Research Australia (NIASRA), School of Mathematics and Applied Statistics, University of Wollongong, Wollongong, Australia; University of Shiga Prefecture, Japan

## Abstract

We assessed and compared abiotic preferences of aquatic macroinvertebrates in three river basins located in Ecuador, Ethiopia and Vietnam. Upon using logistic regression models we analyzed the relationship between the probability of occurrence of five macroinvertebrate families, ranging from pollution tolerant to pollution sensitive, (Chironomidae, Baetidae, Hydroptilidae, Libellulidae and Leptophlebiidae) and physical-chemical water quality conditions. Within the investigated physical-chemical ranges, nine out of twenty-five interaction effects were significant. Our analyses suggested river basin dependent associations between the macroinvertebrate families and the corresponding physical-chemical conditions. It was found that pollution tolerant families showed no clear abiotic preference and occurred at most sampling locations, i.e. Chironomidae were present in 91%, 84% and 93% of the samples taken in Ecuador, Ethiopia and Vietnam. Pollution sensitive families were strongly associated with dissolved oxygen and stream velocity, e.g. Leptophlebiidae were only present in 48%, 2% and 18% of the samples in Ecuador, Ethiopia and Vietnam. Despite some limitations in the study design, we concluded that associations between macroinvertebrates and abiotic conditions can be river basin-specific and hence are not automatically transferable across river basins in the tropics.

## Introduction

Benthic macroinvertebrates have often been used for water quality monitoring and assessment [1], [2]. They are direct measures of stream conditions, integrate human and natural stressors over a long period of time and reflect the quality of their surroundings [3]. Benthic macroinvertebrates can be used as bio-indicators since different macroinvertebrate taxa have different tolerances to pollution [4]. Therefore, benthic macroinvertebrates are suitable for assessing the ecological state of aquatic ecosystems. Macroinvertebrate-based water quality assessment methods have increasingly been applied in national monitoring campaigns (e.g., [5], [6]). In the United Kingdom, macroinvertebrate-based water quality assessment has been applied since 1970 by means of the Chandler Score [7]. In later years, the biological monitoring working party (BMWP; [8]) was developed and accepted as a standard international biotic index. In various regions biotic indices have been applied using a derivate of the BMWP, including the Iberian BMWP [9] and the South African Scoring System (SASS, [5]). In tropical countries, water quality assessment has been primarily performed based on physical-chemical water quality measurements [10]. However, in recent years, macroinvertebrate-based water quality assessments have also been conducted in tropical countries [11].

Most macroinvertebrate-based water quality assessment methods have been developed in temperate climate regions where relationships between environmental variables and the occurrence of macroinvertebrate taxa are well documented. Due to the infancy of the macroinvertebrate-based monitoring and assessment in the tropics [12], the ecological water quality in tropical countries was often assessed based on indices constructed for temperate climate regions [13], [14]. However, it has been shown that applying ecological indices and habitat suitability models from temperate regions to the tropics can lead to uncertain ecological valuations [15], [16]. In this perspective, river basin scale habitat suitability models have recently been developed for the tropics, e.g. in Ecuador [17], Ethiopia [18], [19] and Vietnam [20], [21]. In spite of this progress, limited knowledge is available about the habitat preferences of macroinvertebrates in rivers in the tropics [22]. For instance, it is poorly understood whether associations between macroinvertebrate taxa and environmental conditions vary between river basins and continents. As such, the question remains whether the relations obtained between the macroinvertebrate taxa and the physical-chemical water quality conditions are only valid in the river basin where they have been developed. Therefore, it is questioned whether the abiotic preferences of macroinvertebrates and their responses to environmental pollution are transferable across river basins.

The aim of this study was to assess if abiotic preferences of aquatic macroinvertebrates differed between three river basins in the tropics. We investigated associations between physical-chemical variables and macroinvertebrate occurrences in one Ecuadorian, one Ethiopian and one Vietnamese river basin. Five representative macroinvertebrate taxa, ranging from pollution tolerant to pollution sensitive were selected and their preferences towards environmental conditions were compared using regression-based ecological models.

## Materials and Methods

### Data collection

Samples were taken in the Chaguana river basin (Ecuador), Gilgel Gibe river basin (Ethiopia) and Cau river basin (Vietnam) (Table 1; Figure S1–S3). In each survey physical-chemical water samples and biological samples (macroinvertebrates) were collected. Per river basin, samples were taken at multiple sampling sites along a pollution gradient (Table 2). Details on sampling locations are provided in Table 1 and Figure S1–S3. The number of sampling sites varied for each river basin, but each sampling site was visited twice in each year; once in the wet season and once in the dry season. In total, 60, 104 and 306 samples were taken at 15, 29 and 47 sampling locations in the Vietnamese, Ecuadorian and Ethiopian river basin, respectively (Table 2). Water samples were analysed according to the ISO standards and only the environmental conditions that were monitored in all three river basins were selected for further analysis, being conductivity (µS.cm^−1^), dissolved oxygen concentration (mg.L^−1^), pH (-), stream velocity (m.s^−1^) and water temperature (°C) (Table 2).

**Table 1:** Description of the study areas.

	Ecuador	Ethiopia	Vietnam
**River basin facts**			
Name of river basin	Chaguana	Gilgel Gibe	Cau
Country	Southwest of Ecuador	South to West of Ethiopia	North of Vietnam
Province	El Oro	Oromia	Thai Nguyen
Tributary river	Pagua river 58	Gibe river ^61^	Cau river ^60^
Source	Occidental Andes 58	Ethiopian plateau^62^	Highland of Northern Vietnam^60^
Altitude	2900 m 58	1096–3259 m ^63^	275 m^60^
Surface area	320 km^2^ 58	5371 km^2 18^	6030 km^2 60^
**Climatic description**			
Average annual precipitation	930 mm ^59^	2000 mm ^63^	2063 mm ^60^
Air temperature range	19.9°C–31.4°C ^59^	8.3°C–29.1°C ^64^	10.0°C–39.0°C ^60^
Dry season	May – November ^59^	October – April ^63^	October – May ^60^
Wet season	December-April ^59^	May – September ^63^	June – September ^60^

58 Dominguez-Granda, 2007, ^59^ Matamoros, 2004, ^60^ MONRE, 2006, ^61^ Demissie et al., 2013, ^62^ Uhlenbrook et al., 2010, ^63^ Broothaerts et al., 2012, ^18^ Ambelu et al., 2010, ^64^ Colombo and Maran, 2004.

**Table 2:** Characteristics of biological and physical-chemical sampling.

	Units	Ecuador	Ethiopia	Vietnam
**Number of samples**		104	306	60
**Number of sampling locations**		29	47	15
**Monitoring years**		2005–2006	2006–2011	2009–2010
**Seasons**		wet	wet	wet
		dry	dry	dry
**Physical-chemical variables**				
Stream velocity	m/s	0.5±0.4	0.5±0.3	0.5±0.3
Water temperature	°C	23.3±2.8	20.0±2.5	28.6±2.0
Conductivity	µS/cm	149±152	108±59	235±181
pH	-	6.9±0.4	7.4±0.5	7.0±0.8
Dissolved oxygen	mg/L	7.1±1.3	6.5±1.5	6.4±0.8
**Macroinvertebrate sampling**				
**Chironomidae**				
Number of samples: absent		9	49	4
Number of samples: present		95	257	56
**Baetidae**				
Number of samples: absent		18	80	11
Number of samples: present		86	226	49
**Hydroptilidae**				
Number of samples: absent		88	300	34
Number of samples: present		16	6	26
**Libellulidae**				
Number of samples: absent		50	157	51
Number of samples: present		54	149	9
**Leptophlebiidae**				
Number of samples: absent		54	300	49
Number of samples: present		50	6	11

Descriptive statistics of physical-chemical variables are given as mean values ± standard deviations. Presence-absence records per family and river basin (represented as country) are given as the amount of samples in which macroinvertebrate families were present or absent, respectively.

Benthic macroinvertebrates were sampled, identified and quantified according to the method described in Gabriels et al. [6] which is an internationally accepted kick-sampling procedure for macroinvertebrate sampling. A conical net with a size of 20×30 cm and a mesh size of 300–500 µm, attached to a stick, was used. With the hand net, all accessible aquatic habitats within a stretch of 10–20 m were sampled using the kick sampling method. The sampling effort was equally divided over the different habitats per sampling site. The organisms were identified to family level and this resulted in binary presence-absence data. Subsequently, five macroinvertebrate families, present in the three river basins and ranging from a pollution tolerant family towards a pollution sensitive family based on the BMWP tolerance list, were selected. The five target macroinvertebrate families that were selected were Chironomidae (tolerance class 2 (TS2)), Baetidae (TS4), Hydroptilidae (TS6), Libellulidae (TS8) and Leptophlebiidae (TS10). For a complete overview of the taxa encountered in each river basin we refer to the related publications [17], [18], [20]. No specific permission was needed for our sampling activities nor locations since we were only interested in macroinvertebrates. Sampling did not involve endangered or protected species.

### Data exploration

Boxplots were used for data exploration. The first series of boxplots visualized the seasonality of the physical-chemical variables per river basin. The second series of boxplots summarized for each macroinvertebrate family and per physical-chemical variable and river basin the conditions under which the taxon was present and absent. Boxplots were constructed with the R software [23].

The univariate associations between explanatory variables were assessed with pairwise Spearman's rank correlations, which is often used in ecology due to its nonparametric nature [24].

### Logistic regression model

The occurrence of five macroinvertebrate families was related to the physical-chemical water quality conditions using a regression-based model and these relationships were compared between three river basins situated in the tropics. Logistic regression models (LRMs) were used to infer relationships between occurrences of five aquatic macroinvertebrate families and environmental data. LRMs have been frequently used to model the presence or absence of a species in relation to environmental variables [25]–[27]. Five logistic regression models (LRMs) were constructed, one for each macroinvertebrate family. In a LRM, a binary response variable (here family presence or absence) is modeled as a function of explanatory variables (here environmental conditions). Since multiple samples were collected on the same sampling site, the responses were not mutually independent. Therefore the LRMs were fitted with Generalized Estimating Equations (GEE, [28]) which account for the dependencies of the clustered sampling scheme. All LRMs were fitted with an independent working correlation matrix.

A hierarchical backward elimination model selection method was carried out to build the LRM. The starting model included five physical-chemical variables (conductivity, dissolved oxygen concentration, pH, stream velocity and water temperature), season and river basin (represented as country). In addition to the main effects, two-way interactions between the physical-chemical variables and the river basin as well as between season and river basin were included (Table S1 and S2). First it was tested whether the interactions were present at a 5% level of significance and insignificant interactions were excluded from the model. A significant interaction between a physical-chemical variable and a river basin suggests that the effect of the physical-chemical variable on the occurrence of the family under study differed between river basins. Furthermore, all main effects were included independent from their significance.

Residual plots and the extended Hosmer-Lemeshow test for LRMs based on GEE [29] were used to assess the goodness-of-fit of the LRM. None of the models showed lack-of-fit. Since the Hosmer-Lemeshow test is insensitive to omitted quadratic terms, quadratic effects of the physical-chemical variables were added to the LRM. However, none of these effects were significant.

The outcome of the LRM per family was visualized as the estimated probability that the family was present as a function of a physical-chemical variable. The explanatory variables different from the one on the x-axis, were set to their river basin specific medians and the season to “dry”. The river basin-specific observed range of the corresponding physical-chemical variable were plotted as horizontal boxplots below the response curves and were subdivided between presence and absence points. The gray-colored ends of the response curves indicate extrapolation outside the observed range of the corresponding physical-chemical variable.

For the data exploration, the effects of season and river basin on the continuous physical-chemical variables were assessed using a linear regression model fitted with GEE for accounting for the clustered sampling scheme. Only few p-values were reported in the main text to prevent information overload, all p-values were summarized in Table 3 and 4. All statistical tests were performed at the 5% significance level.

**Table 3:** P-values for river basin-based interaction effects.

	Stream velocity	Water temperature	Conductivity	pH	Dissolved oxygen
**Leptophlebiidae**	**0.008**	0.256	**0.033**	**0.038**	0.121
**Libellulidae**	**0.017**	**<0.001**	0.767	0.055	**0.015**
**Hydroptilidae**	0.905	0.692	**0.015**	0.420	0.262
**Baetidae**	0.126	**0.003**	0.113	0.924	0.582
**Chironomidae**	0.998	**<0.001**	0.999	0.999	0.999

The effect of an explanatory variable was significantly different between the three river basins in case that the p-value for the interaction effect was lower than 0.05. Significant relations are indicated in bold.

**Table 4:** Estimates of the main effects for Chironomidae, Baetidae, Hydroptilidae, Libellulidae, Leptophlebiidae.

	Seasonality	Stream velocity	Water temperature	Conductivity	pH	Dissolved oxygen
**Chironomidae**						
**Ecuador**	−0.873 (0.265)	**1.358 (0.016)**	0.091 (0.455)	−0.002 (0.316)	0.149 (0.697)	−0.108 (0.290)
**Ethiopia**	−0.313 (0.402)	**1.358 (0.016)**	−0.078 (0.151)	−0.002 (0.316)	0.149 (0.697)	−0.108 (0.290)
**Vietnam**	35.838 (>0.999)	**1.358 (0.016)**	−**1.649 (<0.001)**	−0.002 (0.317)	0.149 (0.697)	−0.108 (0.290)
**Baetidae**						
**Ecuador**	0.869 (0.184)	**1.289 (0.004)**	0.031 (0.700)	−0.002 (0.121)	**0.554 (0.030)**	−0.065 (0.410)
**Ethiopia**	**0.704 (0.019)**	**1.289 (0.004)**	−0.049 (0.407)	−0.002 (0.121)	**0.554 (0.030)**	−0.065 (0.410)
**Vietnam**	0.163 (0.804)	**1.289 (0.004)**	−0.351 (0.133)	−0.002 (0.121)	**0.554 (0.030)**	−0.065 (0.410)
**Hydroptilidae**						
**Ecuador**	−0.126 (0.864)	0.829 (0.223)	−0.062 (0.531)	−0.003 (0.297)	**0.963 (0.020)**	0.179 (0.519)
**Ethiopia**	−1.463 (0.203)	0.829 (0.223)	−0.062 (0.531)	−**0.020 (0.042)**	**0.963 (0.020)**	0.179 (0.519)
**Vietnam**	−0.408 (0.438)	0.829 (0.223)	−0.062 (0.531)	−**0.009 (0.031)**	**0.963 (0.020)**	0.179 (0.519)
**Libellulidae**						
**Ecuador**	−**1.788 (0.005)**	**2.488 (0.005)**	**0.387 (0.002)**	−0.0001 (0.961)	**0.674 (0.006)**	**0.481 (0.023)**
**Ethiopia**	0.192 (0.445)	−0.279 (0.534)	−**0.094 (0.021)**	−0.0001 (0.961)	**0.674 (0.006)**	−0.046 (0.619)
**Vietnam**	−1.369 (0.268)	**3.780 (0.021)**	−**0.708 (<0.001)**	−0.0001 (0.961)	**0.674 (0.006)**	0.135 (0.815)
**Leptophlebiidae**						
**Ecuador**	**2.147 (0.006)**	**3.679 (0.003)**	−**0.291 (0.048)**	−0.010 (0.072)	0.503 (0.356)	**1.371 (0.002)**
**Ethiopia**	1.385 (0.232)	0.694 (0.750)	−**0.291 (0.048)**	−0.024 (0.370)	−0.223 (0.840)	**1.371 (0.002)**
**Vietnam**	1.426 (0.100)	−1.703 (0.410)	−**0.291 (0.048)**	0.002 (0.355)	**1.943 (0.004)**	**1.371 (0.002)**

Corresponding p-values are given between brackets; effects were tested at the 5% significance level. Significant relations are indicated in bold.

## Results

### Data exploration and correlation analysis

The observed range of physical-chemical water quality conditions was not always equal between river basins. Seasonal effects were observed for all physical-chemical variables and the seasonal effect of conductivity and stream velocity was similar for the three river basins (Table S1; Figure S4–S8). Dissolved oxygen (DO) concentration and water temperature ranges differed between river basins (Table 2; Figure S9–S33). The average stream velocity was higher during the wet season compared to the dry season (p<0.01; Table S1; Figure S4). The average conductivity in wet season was lower than in dry season in the Ecuadorian river basin (p<0.01) and the Ethiopian river basin (p<0.01). In the Vietnamese river basin, however, this difference was not significant (p = 0.39; Figure S6). The seasonal effect differed per river basin for water temperature (Figure S5), pH (Figure S7) and DO concentration (Figure S8). For instance, dry season DO concentrations tended to exceed wet season conditions in the Ecuadorian river basin, but in the Ethiopian river basin the opposite was observed (Table S1; Figure S8). For both seasons there was no significant difference in mean stream velocity between river basins (p = 0.32 and p = 0.78, Table S2; Figure S4). For DO there was a significant difference between river basins for the dry season (p<0.01), but not for the wet season (p = 0.16). In contrast, the mean water temperature, pH and conductivity were significantly different across river basins for both seasons.

**Figure 1:**
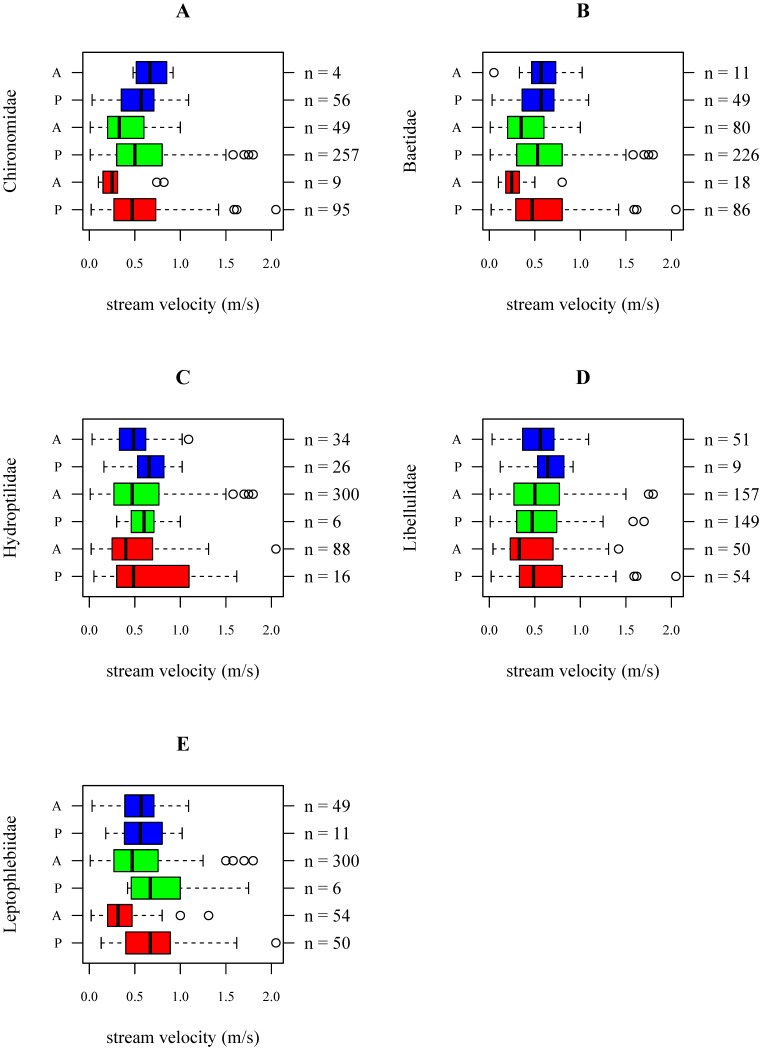
Stream velocity data for which Chironomidae (A), Baetidae (B), Hydroptilidae (C), Libellulidae (D) and Leptophlebiidae (E) are found to be present (denoted by P on the left axis) and absent (denoted by A on the left axis) in Ecuador (red), Ethiopia (green) and Vietnam (blue). The sample sizes per boxplot are shown on the right axis.

**Figure 2:**
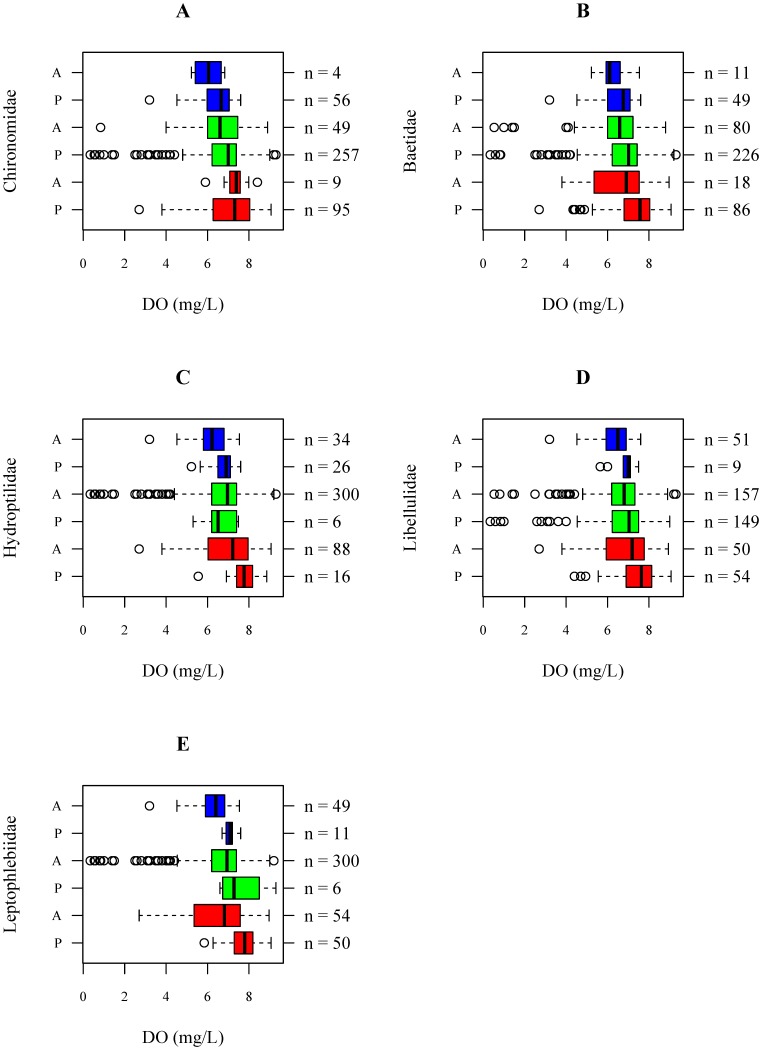
Dissolved oxygen concentrations for which Chironomidae (A), Baetidae (B), Hydroptilidae (C), Libellulidae (D) and Leptophlebiidae (E) are found to be present (denoted by P on the left axis) and absent (denoted by A on the left axis) in Ecuador (red), Ethiopia (green) and Vietnam (blue). The sample sizes per boxplot are shown on the right axis.

The prevalence values, known as the relative frequencies of occurrence of taxa (here families, [30]), did not always approximate 0.5, i.e. some families occurred rarely or ubiquitously (Table 2). Furthermore, it was found that the range of preferred physical-chemical conditions differed between families (Figure 1A–1E; Figure 2A–2E; Figure S9–S33). For less pollution sensitive families such as Chironomidae (TS2) and Baetidae (TS4) a wide range of suitable physical-chemical conditions has been observed. More sensitive families such as Leptophlebiidae (TS10) were only present within a more narrow range of stream velocity, conductivity and DO concentration (Figure 1A–1E; Figure 2A–2E; Figure S9–S33).

Based on the correlation analysis no variables were discarded from the dataset as most correlations were “weak” since they were smaller than 0.4 in absolute values [21], [24]. Only conductivity and water temperature (r = 0.41, p<0.01) and conductivity and DO (r = −0.42, p<0.01) were moderately correlated (Table 5).

**Table 5:** Correlation coefficients of the physical-chemical variables.

	Stream velocity	Water temperature	pH	Conductivity	Dissolved oxygen
**Stream velocity**	/				
**Water temperature**	**−0.14 (<0.001)**	/			
**pH**	0.03 (0.571)	**−0.21 (<0.001)**	/		
**Conductivity**	**−0.39 (<0.001)**	**0.41 (<0.001)**	**0.15 (0.001)**	/	
**Dissolved oxygen**	**0.28 (<0.001)**	**−0.28 (<0.001)**	**0.23 (<0.001)**	**−0.42 (<0.001)**	/

Corresponding p-values are given between brackets; effects were tested at the 5% significance level. Significant relations are indicated in bold.

### Logistic regression model

#### Chironomidae (TS2)

**Figure 3:**
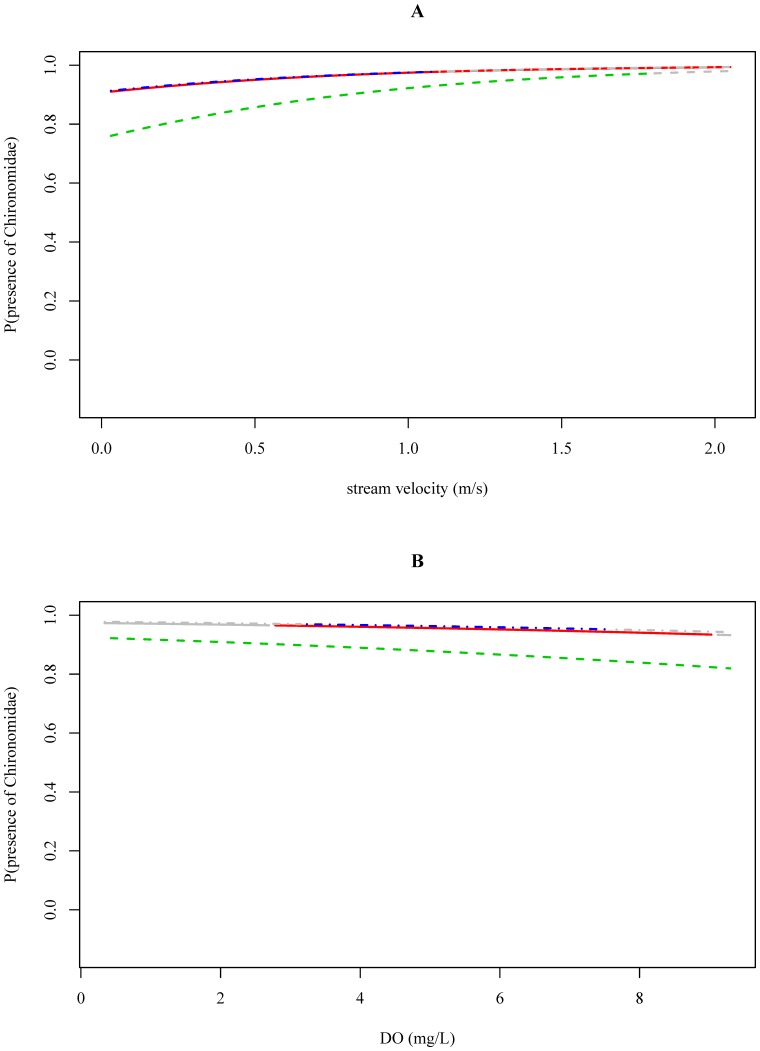
The probability of Chironomidae being present in relation to the stream velocity (A) and dissolved oxygen data (B) measured in Ecuador (red, solid), Ethiopia (green, dashed) and Vietnam (blue, dotdashed). The gray-colored ends of the response curves indicate extrapolation outside the observed physical-chemical range in the corresponding river basin.

For Chironomidae there was an interaction effect between river basin and water temperature (p<0.01), i.e. the effect of water temperature on the occurrence differed between river basins (Table 3). Although increasing water temperatures were associated with a lower probability that Chironomidae were present in the Vietnamese river basin (p<0.01), in the Ecuadorian river basin and in the Ethiopian river basin this effect was not observed (p = 0.46 and p = 0.15, Table 4; Figure S35). However, note that Chironomidae were absent in only 4 out of 60 samples from the Vietnamese river basin (Figure S35). We also found a positive association between the stream velocity and the probability of occurrence of Chironomidae (p = 0.02), i.e. a higher stream velocity was associated with a higher probability that Chironomidae were present (Table 4; Figure 3A). The effect of stream velocity was similar between river basins, i.e. regardless the stream velocity, Chironomidae were always likely to be present in the three river basins (probabilities between 0.8 and 1.0 in Figure 3A and S34). The latter is also reflected in the boxplots, which indicate that absence data for Chironomidae is low (4 out of 60 in Vietnam, 49 out of 306 in Ethiopia and 9 out of 104 in Ecuador; Figure 1A). Within the investigated physical-chemical range, conductivity, pH, and DO concentrations were not associated with the probability of occurrence of Chironomidae (Figure 3B; Figure S36–S38).

#### Baetidae (TS4)

The effect of water temperature on the probability of occurrence of Baetidae differed between river basins (p<0.01; Table 3). Indeed, when looking at the response curves (Figure S40) it is clear that within the observed water temperature ranges for each river basin, the slopes of the curves differ. However, probabilities of occurrence always exceeded the value of 0.6 in any river basin regardless the water temperature data. Moreover, within river basins no significant association was found between the presence of Baetidae and water temperature (Table 4). An increased stream velocity and pH was associated with an increased probability of occurrence of Baetidae in all three river basins (p = 0.01 and p = 0.03, respectively; Table 4; Figure S39 and S42). Again, similar as for the Chironomidae, we found that Baetidae were likely to be present in all the three river basins (Figure 1B, S39 and S42). In the investigated range, no significant association was found between the probability of occurrence of Baetidae and DO concentration (p = 0.58, Figure S43) or conductivity (p = 0.11, Figure S41).

#### Hydroptilidae (TS6)

Associations between the DO concentration, pH, stream velocity and water temperature relative to the probability of occurrence of Hydroptilidae were similar across river basins (Table 3). Only the effect of conductivity differed between river basins (p = 0.02; Table 4), i.e. higher conductivities were associated with a lower probability that Hydroptilidae were present; this was observed for the Vietnamese river basin (p = 0.03) and the Ethiopian river basin (p = 0.04; Table 4; Figure S46). Furthermore, a more alkaline pH was associated with a higher occurrence of Hydroptilidae (p = 0.02; Table 4; Figure S47). No significant associations were found between water temperature (p = 0.69; Figure S45), stream velocity (p = 0.90; Figure S44) and DO concentration (p = 0.26; Figure S48) and the probability of occurrence of Hydroptilidae (Table 4).

#### Libellulidae (TS8)

**Figure 4:**
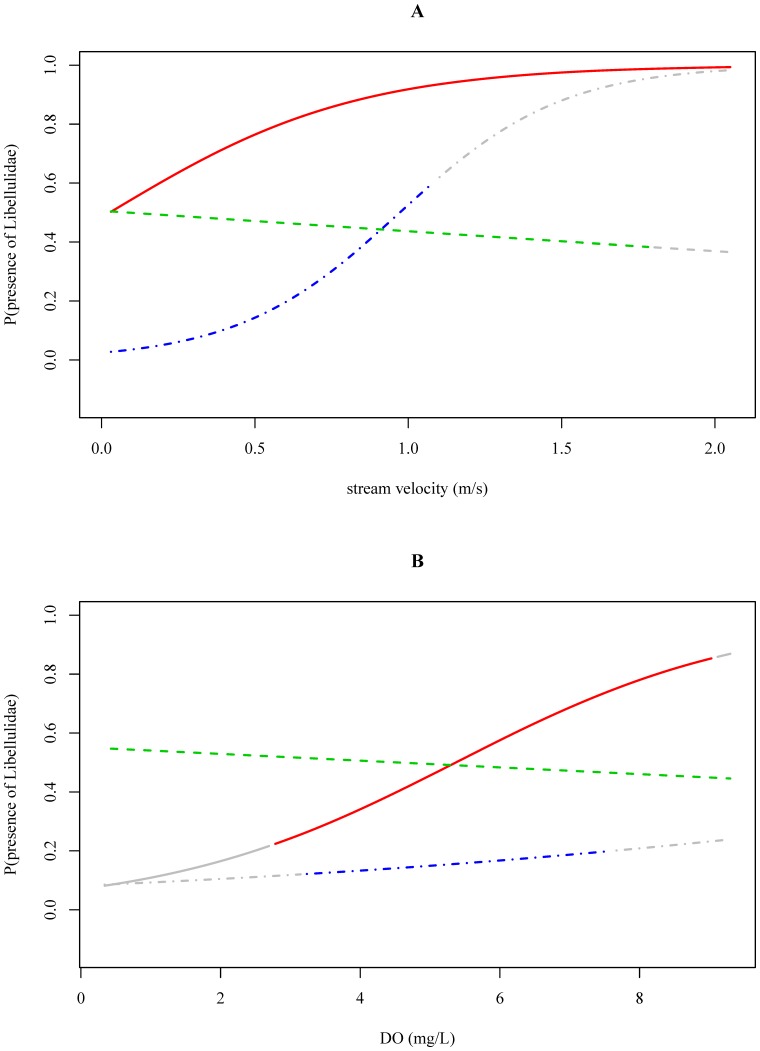
The probability of Libellulidae being present in relation to stream velocity (A) and dissolved oxygen data (B) measured in Ecuador (red, solid), Ethiopia (green, dashed) and Vietnam (blue, dotdashed). The gray-colored ends of the response curves indicate extrapolation outside the observed physical-chemical range in the corresponding river basin.

The associations between water temperature (p<0.01), stream velocity (p = 0.02) and DO concentration (p = 0.02) and the probability of occurrence of Libellulidae differed between river basins (Table 3). Whereas in the Ecuadorian river basin, the probability that Libellulidae occurred increased with increasing water temperatures (p<0.01), in the Ethiopian and Vietnamese river basin inverse associations were observed (p = 0.02 and p<0.01, respectively; Table 4; Figure S50). Concerning stream velocity, Libellulidae were likely to occur at higher stream velocities in the Ecuadorian and Vietnamese river basin (p<0.01 and p = 0.02, respectively), but in the Ethiopian river basin this was not observed (Table 4; Figure 4A and S49). Increasing DO concentrations were associated with a higher probability of occurrence of Libellulidae in the Ecuadorian river basin (p = 0.02), but for the Vietnamese (p = 0.82) and Ethiopian river basin (p = 0.62) this association was not significant (Figure 4B and S53). The associations between conductivity and pH and the probability of occurrence of Libellulidae were similar between river basins (Table 4; Figure S51 and S52).

#### Leptophlebiidae (TS10)

**Figure 5:**
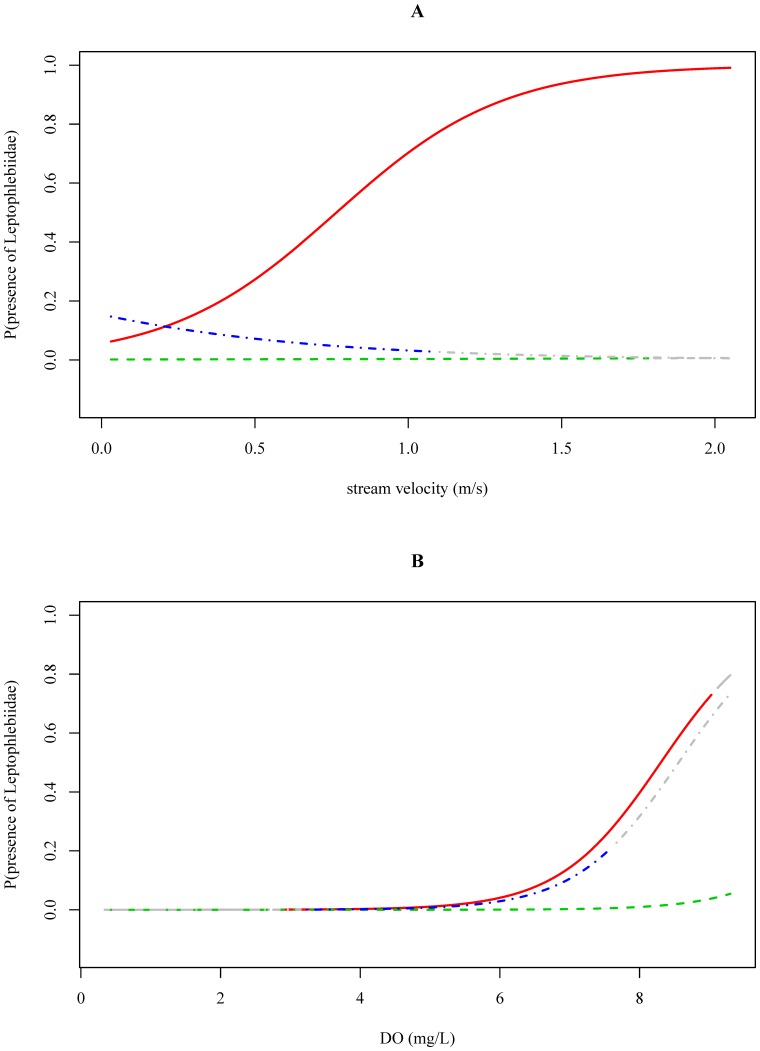
The probability of Leptophlebiidae being present in relation to stream velocity (A) and dissolved oxygen data (B) measured in Ecuador (red, solid), Ethiopia (green, dashed) and Vietnam (blue, dotdashed). The gray-colored ends of the response curves indicate extrapolation outside the observed physical-chemical range in the corresponding river basin.

Associations between stream velocity (Figure 1E, 5A and S54), pH (Figure S57) and conductivity (Figure S56) and the probability of occurrence of Leptophlebiidae were different amongst river basins (p = 0.01, p = 0.04 and p = 0.03; Table 3). However, when interpreting the response curves it was found that the prevalence of Leptophlebiidae was not balanced in the Vietnamese and Ethiopian river basin (Table 2). For streams situated in the Ecuadorian river basin Leptophlebiidae were more likely to occur at higher stream velocities (p<0.01). In the Vietnamese and Ethiopian river basin, however, these effects were not observed (Table 4; Figure 5A and S54), probably due the limited amount of presence data in these river basins. The association between conductivity and the probability of occurrence of Leptophlebiidae suggested the highest probability of occurrence at lowest conductivities in the Vietnamese river basin (Figure S56). For all three river basins, an increase in DO concentration was positively associated with the occurrence of Leptophlebiidae (p<0.01; Table 4; Figure 5B and S58). Associations between Leptophlebiidae and water temperature were similar between the three river basins (Figure S55).

## Discussion

For pollution tolerant macroinvertebrate families (e.g. Chironomidae (TS2)) only few significant associations between physical-chemical conditions and the occurrence of the families were found (Table 4) suggesting their occurrence at a wide range of physical-chemical conditions. This was also reflected in the data exploration as Chironomidae were present in 91%, 84% and 93% of the samples taken in the Ecuadorian, Ethiopian and Vietnamese river basin, respectively (Table 2). Chironomidae are known to be tolerant to disturbance, allowing them to occur in impacted environments [32]. Due to their physiological adaptations they have the ability to survive low oxygen conditions (Figure 2A and 3B, [33], [34]). Because of their pollution tolerant nature, it was surprising to conclude that high stream velocities are associated with an increased occurrence of Chironomidae (Figure 1A, 3A and S34). However, in this study we only identified macroinvertebrates to family level. Chironomidae are represented by many species that show a different sensitivity to environmental pollution [33], which might explain the weak association between environmental variables and the occurrence of Chironomidae. Baetidae, present in 83%, 74% and 82% of the samples taken in the Ecuadorian, Ethiopian and Vietnamese river basin, respectively (Table 2), were more sensitive to pollution compared to Chironomidae. The habitat preference of Baetidae was mainly determined by stream velocity and pH. Baetidae have often been reported as one of the most acid-sensitive macroinvertebrate families [35]. As taxa sensitivity to pollution increased, more significant associations were found between environmental conditions and the occurrence of macroinvertebrate families (Table 4). For instance, a significant positive association between DO concentrations and stream velocities and the occurrence of Libellulidae (TS8) and Leptophlebiidae (TS10) was found (Figure 4 and 5). DO concentration is a general indicator of water quality [34], [36] and also in other studies it was found that DO concentrations play a crucial role when analyzing the occurrence of macroinvertebrates (e.g., [37–39)]. In streams in Malaysia, Rawi et al. [40] found that DO concentration and stream velocity were crucial variables when explaining macroinvertebrate diversity. For other biological communities DO levels are also important, e.g. most fish require a DO concentration of at least 5 mg.L^−1^ for optimal health [41].

Habitat suitability of macro-invertebrates probably depends on more factors than those included in our statistical analysis. For instance, additional to the variables that were included in our analysis, Al-Shami et al. [42] also integrated geographical variables such as longitude, latitude and altitude. Blanchette and Pearson [43] related macroinvertebrate assemblages to chlorophyll, suspended solids, turbidity and nutrient data. Kasangaki et al. [44] studied benthic macroinvertebrates in Uganda and used DO concentration, conductivity, pH, turbidity and water temperature together with some hydromorphological stream characteristics. As such, physical-chemical variables used by Kasangaki et al. [44] are similar to those included in our statistical analysis. Conductivity can be seen as a general measure for disturbance as it integrates pollution related variables like minerals and inorganic pollutants [37]. For instance, Melo [45] concluded that stream size data and conductivity explained most of the variability in the macroinvertebrate community in a stream in the tropics. In Colombia, Holguin-Gonzalez et al. [26] used DO concentration and stream velocity to predict the presence of macroinvertebrates. According to Flecker and Feifarek [46] hydrodynamics (including stream velocity) play a crucial role in structuring tropic macroinvertebrate communities. Therefore, although only five physical-chemical variables were used in the models, explanatory variables embedded in the LRMs covered a wide spectrum of potential impacts.

Seasonal changes of environmental variables were taken into account [10], [43]. Two samples in each year and at each sampling location (in the wet and dry season) were collected for two consecutive years for each river basin (Table S1; Figure S4–S8). By including these, potential seasonal uncertainties were integrated in our statistical analysis.

The outcome of the LRM per family was visualized as the estimated probability that the family was present as a function of a physical-chemical variable (e.g. Figure 3–5). Within the physical-chemical ranges that were investigated and for the studied macroinvertebrate families (see boxplots and colored zone of the response curves), nine out of twenty-five interaction effects were significant (Table 3). Hence, the corresponding variable-macroinvertebrate relationships were different between river basins and suggested different habitat preferences for the investigated families. For Libellulidae for instance, it was found that the effect of dissolved oxygen was different between river basins (Table 3; Figure 4B). Increasing DO concentrations were associated with a higher probability that Libellulidae were present in Ecuadorian river basin (p = 0.02), but in the Vietnamese and Ethiopian river basin this relationship was not significant (p = 0.82 and p = 0.62). In the Vietnamese and Ethiopian river basin, Libellulidae were less responsive to shifts in DO concentrations as the probability of occurrence hardly changed at varying DO concentrations (Table 4, Figure 4B). In the Vietnamese river basin, this can be explained by the relative few observed presences of some taxa, i.e. Libellulidae were found in only nine out of sixty samples (Table 2). In the Ethiopian river basin however, presence and absence data were more equally represented. Concerning the association between stream velocity and the occurrence of Libellulidae it was found that Libellulidae favored high currents in the Ecuadorian and Vietnamese river basin (p = 0.01 and p = 0.02, respectively). In the Ethiopian river basin, this relationship was not observed (Table 4; Figure 4A). However, a negative relationship did not mean that Libellulidae were absent in the Ethiopian river basin as there was still an estimated probability of 50% that Libellulidae were present within the range of observations (Figure 4A).

The fact that river basin dependent associations were found is not surprising. For instance, Bonada et al. [47] found that the response of macroinvertebrates to pollution was different between Mediterranean ecoregions. Also in the US, Zuellig and Schmidt [48] found dissimilar regional benthic invertebrate community compositions. Moreover, it was stated by Thorne and Williams [13] that due to untreated domestic and urban effluents in developing countries the relationship between individual physical-chemical variables and macroinvertebrates present can be extremely complex. Hence, this may lead to river basin specific associations between abiotic conditions and the biological communities.

Since the range of the observed physical-chemical conditions were not always equal between river basins, in some cases extrapolations outside the observed range were shown (Figure 4A). For instance, water temperature measurements significantly differed between the three river basins (Table S2). Hence, it was found that the association between the occurrence of Libellulidae and water temperature was significantly different between river basins (Table 3). However, at the intersection of the three ranges, the estimated probabilities were similar (Figure S50). Therefore, major differences in the estimated probabilities across river basins are likely to be attributed to difference in the range of the water temperature between those basins.

No relation was found between the occurrence of Leptophlebiidae and stream velocity in the Ethiopian and the Vietnamese river basin. However, according to the data Leptophlebiidae occurred in both river basins in upstream sampling sites with a moderate to high stream velocity. The reason for the non-significant relation is the prevalence of the present-points. In the Vietnamese and Ethiopian river basin, Leptophlebiidae were only present in 18% and 2% of the samples, respectively. Consequently, the occurrence of Leptophlebiidae seems statistically not associated with stream velocity. Therefore, due to unbalanced dataset prevalence it is possible that not all models obtained represent true ecological relations [30]. As such, LRMs' outcomes should be carefully interpreted, but for those families and river basins where prevalence was in balance, the associations found, were ecologically relevant.

Presence and absence data are commonly used in macroinvertebrate research (e.g., [48]–[51]) as they provide a basic inventory to explore the species' ecology [52]. However, recently Howard et al. [53] concluded that using abundance data instead of presence and absence data could make models more informative since abundance data provide additional insight with regard to the population dynamics. Although it may result in a gain of information [54], including abundance data will also increase the variability of the response variable. In this perspective, Flecker and Feifarek [46] concluded that the abundance of macroinvertebrate families differed seasonally between one and four orders of magnitude. In this research the sample size (60–306) was relatively limited compared to the number of sampling locations (15–47) [55] and may not cover the entire seasonality in the macroinvertebrate abundance data. Therefore, it was more appropriate to analyze presence-absence data, which are more robust against seasonal changes. Moreover, logistic models have been frequently applied (e.g., [24]–[26]) as they are well suited to provide predictions of the probability of occurrence based on presence and absence data [52].

As an alternative to the conventional taxonomic division of the benthic invertebrates one can use functional traits to categorize macroinvertebrate assemblages (e.g., [48], [56]). Such a trait-based approach is a promising alternative to taxonomy-based approaches for assessing the conditions of freshwater ecosystems [57]. However, trait-based studies have often a limited scope and further testing is needed to establish their reliability [48], especially in regions where knowledge on macroinvertebrates is poor.

Overall, LRMs based on GEE are a flexible way to model the probability of occurrence of macroinvertebrates as a function of environmental variables. We revealed similar as well as dissimilar abiotic preferences of macroinvertebrates between the three river basins, but these estimated probabilities are restricted to the observed range of the predictor within each river basin. In conclusion we found that associations between macroinvertebrates and abiotic conditions can be river basin-specific.

## Supporting Information

Figure S1 **Sampling sites in the Chaguana river basin in Ecuador.**
(DOCX)

Figure S2 **Sampling sites in the Gilgel Gibe river basin in Ethiopia.**
(DOCX)

Figure S3 **Sampling sites in the Cau river basin in Vietnam.**
(DOCX)

Figure S4 **Boxplots showing the seasonality (dry (blue) and wet (red) season) of the stream velocity.**
(DOCX)

Figure S5 **Boxplots showing the seasonality (dry (blue) and wet (red) season) of the water temperature.**
(DOCX)

Figure S6 **Boxplots showing the seasonality (dry (blue) and wet (red) season) of the conductivity.**
(DOCX)

Figure S7 **Boxplots showing the seasonality (dry (blue) and wet (red) season) of the pH.**
(DOCX)

Figure S8 **Boxplots showing the seasonality (dry (blue) and wet (red) season) of the dissolved oxygen.**
(DOCX)

Figure S9 **Boxplots indicate the observed stream velocity in Ecuador (red), Ethiopia (green) and Vietnam (blue) at which Chironomidae are found to be present (denoted by P on the left axis) and absent (denoted by A on the left axis).** The sample sizes per boxplot are shown on the right axis.
(DOCX)

Figure S10 **Boxplots indicate the observed water temperature in Ecuador (red), Ethiopia (green) and Vietnam (blue) at which Chironomidae are found to be present (denoted by P on the left axis) and absent (denoted by A on the left axis).** The sample sizes per boxplot are shown on the right axis.
(DOCX)

Figure S11 **Boxplots indicate the observed conductivity in Ecuador (red), Ethiopia (green) and Vietnam (blue) at which Chironomidae are found to be present (denoted by P on the left axis) and absent (denoted by A on the left axis).** The sample sizes per boxplot are shown on the right axis.
(DOCX)

Figure S12 **Boxplots indicate the observed pH in Ecuador (red), Ethiopia (green) and Vietnam (blue) at which Chironomidae are found to be present (denoted by P on the left axis) and absent (denoted by A on the left axis).** The sample sizes per boxplot are shown on the right axis.
(DOCX)

Figure S13 **Boxplots indicate the observed DO concentrations in Ecuador (red), Ethiopia (green) and Vietnam (blue) at which Chironomidae are found to be present (denoted by P on the left axis) and absent (denoted by A on the left axis).** The sample sizes per boxplot are shown on the right axis.
(DOCX)

Figure S14 **Boxplots indicate the observed stream velocity in Ecuador (red), Ethiopia (green) and Vietnam (blue) at which Baetidae are found to be present (denoted by P on the left axis) and absent (denoted by A on the left axis).** The sample sizes per boxplot are shown on the right axis.
(DOCX)

Figure S15 **Boxplots indicate the observed water temperature in Ecuador (red), Ethiopia (green) and Vietnam (blue) at which Baetidae are found to be present (denoted by P on the left axis) and absent (denoted by A on the left axis).** The sample sizes per boxplot are shown on the right axis.
(DOCX)

Figure S16 **Boxplots indicate the observed conductivity in Ecuador (red), Ethiopia (green) and Vietnam (blue) at which Baetidae are found to be present (denoted by P on the left axis) and absent (denoted by A on the left axis).** The sample sizes per boxplot are shown on the right axis.
(DOCX)

Figure S17 **Boxplots indicate the observed pH in Ecuador (red), Ethiopia (green) and Vietnam (blue) at which Baetidae are found to be present (denoted by P on the left axis) and absent (denoted by A on the left axis).** The sample sizes per boxplot are shown on the right axis.
(DOCX)

Figure S18 **Boxplots indicate the observed DO concentrations in Ecuador (red), Ethiopia (green) and Vietnam (blue) at which Baetidae are found to be present (denoted by P on the left axis) and absent (denoted by A on the left axis).** The sample sizes per boxplot are shown on the right axis.
(DOCX)

Figure S19 **Boxplots indicate the observed stream velocity in Ecuador (red), Ethiopia (green) and Vietnam (blue) at which Hydroptilidae are found to be present (denoted by P on the left axis) and absent (denoted by A on the left axis).**
(DOCX)

Figure S20 **Boxplots indicate the observed water temperature in Ecuador (red), Ethiopia (green) and Vietnam (blue) at which Hydroptilidae are found to be present (denoted by P on the left axis) and absent (denoted by A on the left axis).** The sample sizes per boxplot are shown on the right axis.
(DOCX)

Figure S21 **Boxplots indicate the observed conductivity in Ecuador (red), Ethiopia (green) and Vietnam (blue) at which Hydroptilidae are found to be present (denoted by P on the left axis) and absent (denoted by A on the left axis).** The sample sizes per boxplot are shown on the right axis.
(DOCX)

Figure S22 **Bottom: Boxplots indicate the observed pH in Ecuador (red), Ethiopia (green) and Vietnam (blue) at which Hydroptilidae are found to be present (denoted by P on the left axis) and absent (denoted by A on the left axis).**
(DOCX)

Figure S23 **Boxplots indicate the observed DO concentrations in Ecuador (red), Ethiopia (green) and Vietnam (blue) at which Hydroptilidae are found to be present (denoted by P on the left axis) and absent (denoted by A on the left axis).** The sample sizes per boxplot are shown on the right axis.
(DOCX)

Figure S24 **Boxplots indicate the observed stream velocity in Ecuador (red), Ethiopia (green) and Vietnam (blue) at which Libellulidae are found to be present (denoted by P on the left axis) and absent (denoted by A on the left axis).** The sample sizes per boxplot are shown on the right axis.
(DOCX)

Figure S25 **Boxplots indicate the observed water temperature in Ecuador (red), Ethiopia (green) and Vietnam (blue) at which Libellulidae are found to be present (denoted by P on the left axis) and absent (denoted by A on the left axis).** The sample sizes per boxplot are shown on the right axis.
(DOCX)

Figure S26 **Boxplots indicate the observed conductivity in Ecuador (red), Ethiopia (green) and Vietnam (blue) at which Libellulidae are found to be present (denoted by P on the left axis) and absent (denoted by A on the left axis).** The sample sizes per boxplot are shown on the right axis.
(DOCX)

Figure S27 **Boxplots indicate the observed pH in Ecuador (red), Ethiopia (green) and Vietnam (blue) at which Libellulidae are found to be present (denoted by P on the left axis) and absent (denoted by A on the left axis).** The sample sizes per boxplot are shown on the right axis.
(DOCX)

Figure S28 **Boxplots indicate the observed DO concentrations in Ecuador (red), Ethiopia (green) and Vietnam (blue) at which Libellulidae are found to be present (denoted by P on the left axis) and absent (denoted by A on the left axis).** The sample sizes per boxplot are shown on the right axis.
(DOCX)

Figure S29 **Boxplots indicate the observed stream velocity in Ecuador (red), Ethiopia (green) and Vietnam (blue) at which Leptophlebiidae are found to be present (denoted by P on the left axis) and absent (denoted by A on the left axis).** The sample sizes per boxplot are shown on the right axis.
(DOCX)

Figure S30 **Boxplots indicate the observed water temperature in Ecuador (red), Ethiopia (green) and Vietnam (blue) at which Leptophlebiidae are found to be present (denoted by P on the left axis) and absent (denoted by A on the left axis).** The sample sizes per boxplot are shown on the right axis.
(DOCX)

Figure S31 **Boxplots indicate the observed conductivity in Ecuador (red), Ethiopia (green) and Vietnam (blue) at which Leptophlebiidae are found to be present (denoted by P on the left axis) and absent (denoted by A on the left axis).**
(DOCX)

Figure S32 **Boxplots indicate the observed pH in Ecuador (red), Ethiopia (green) and Vietnam (blue) at which Leptophlebiidae are found to be present (denoted by P on the left axis) and absent (denoted by A on the left axis).** The sample sizes per boxplot are shown on the right axis.
(DOCX)

Figure S33 **Boxplots indicate the observed DO concentrations in Ecuador (red), Ethiopia (green) and Vietnam (blue) at which Leptophlebiidae are found to be present (denoted by P on the left axis) and absent (denoted by A on the left axis).** The sample sizes per boxplot are shown on the right axis.
(DOCX)

Figure S34 **The probability of Chironomidae being present in relation to the stream velocity measured in Ecuador (red, solid), Ethiopia (green, dashed) and Vietnam (blue, dotdashed).** The gray-colored ends of the response curves indicate extrapolation outside the observed physical-chemical range in the corresponding river basin.
(DOCX)

Figure S35 **The probability of Chironomidae being present in relation to the water temperature measured in Ecuador (red, solid), Ethiopia (green, dashed) and Vietnam (blue, dotdashed).** The gray-colored ends of the response curves indicate extrapolation outside the observed physical-chemical range in the corresponding river basin.
(DOCX)

Figure S36 **The probability of Chironomidae being present in relation to the conductivity measured in Ecuador (red, solid), Ethiopia (green, dashed) and Vietnam (blue, dotdashed).** The gray-colored ends of the response curves indicate extrapolation outside the observed physical-chemical range in the corresponding river basin.
(DOCX)

Figure S37 **The probability of Chironomidae being present in relation to the pH measured in Ecuador (red, solid), Ethiopia (green, dashed) and Vietnam (blue, dotdashed).** The gray-colored ends of the response curves indicate extrapolation outside the observed physical-chemical range in the corresponding river basin.
(DOCX)

Figure S38 **The probability of Chironomidae being present in relation to dissolved oxygen (DO) concentration measured in Ecuador (red, solid), Ethiopia (green, dashed) and Vietnam (blue, dotdashed).** The gray-colored ends of the response curves indicate extrapolation outside the observed physical-chemical range in the corresponding river basin.
(DOCX)

Figure S39 **The probability of Baetidae being present in relation to stream velocity measured in Ecuador (red, solid), Ethiopia (green, dashed) and Vietnam (blue, dotdashed).** The gray-colored ends of the response curves indicate extrapolation outside the observed physical-chemical range in the corresponding river basin.
(DOCX)

Figure S40 **The probability of Baetidae being present in relation to water temperature measured in Ecuador (red, solid), Ethiopia (green, dashed) and Vietnam (blue, dotdashed).** The gray-colored ends of the response curves indicate extrapolation outside the observed physical-chemical range in the corresponding river basin.
(DOCX)

Figure S41 **The probability of Baetidae being present in relation to conductivity measured in Ecuador (red, solid), Ethiopia (green, dashed) and Vietnam (blue, dotdashed).** The gray-colored ends of the response curves indicate extrapolation outside the observed physical-chemical range in the corresponding river basin.
(DOCX)

Figure S42 **The probability of Baetidae being present in relation to pH measured in Ecuador (red, solid), Ethiopia (green, dashed) and Vietnam (blue, dotdashed).** The gray-colored ends of the response curves indicate extrapolation outside the observed physical-chemical range in the corresponding river basin.
(DOCX)

Figure S43 **The probability of Baetidae being present in relation to dissolved oxygen (DO) concentration measured in Ecuador (red, solid), Ethiopia (green, dashed) and Vietnam (blue, dotdashed).** The gray-colored ends of the response curves indicate extrapolation outside the observed physical-chemical range in the corresponding river basin.
(DOCX)

Figure S44 **The probability of Hydroptilidae being present in relation to stream velocity measured in Ecuador (red, solid), Ethiopia (green, dashed) and Vietnam (blue, dotdashed).** The gray-colored ends of the response curves indicate extrapolation outside the observed physical-chemical range in the corresponding river basin.
(DOCX)

Figure S45 **The probability of Chironomidae being present in relation to the Hydroptilidae measured in Ecuador (red, solid), Ethiopia (green, dashed) and Vietnam (blue, dotdashed).** The gray-colored ends of the response curves indicate extrapolation outside the observed physical-chemical range in the corresponding river basin.
(DOCX)

Figure S46 **The probability of Hydroptilidae being present in relation to the conductivity measured in Ecuador (red, solid), Ethiopia (green, dashed) and Vietnam (blue, dotdashed).** The gray-colored ends of the response curves indicate extrapolation outside the observed physical-chemical range in the corresponding river basin.
(DOCX)

Figure S47 **The probability of Hydroptilidae being present in relation to pH measured in Ecuador (red, solid), Ethiopia (green, dashed) and Vietnam (blue, dotdashed).** The gray-colored ends of the response curves indicate extrapolation outside the observed physical-chemical range in the corresponding river basin.
(DOCX)

Figure S48 **The probability of Hydroptilidae being present in relation to dissolved oxygen (DO) concentration measured in Ecuador (red, solid), Ethiopia (green, dashed) and Vietnam (blue, dotdashed).** The gray-colored ends of the response curves indicate extrapolation outside the observed physical-chemical range in the corresponding river basin.
(DOCX)

Figure S49 **The probability of Libellulidae being present in relation to stream velocity measured in Ecuador (red, solid), Ethiopia (green, dashed) and Vietnam (blue, dotdashed).** The gray-colored ends of the response curves indicate extrapolation outside the observed physical-chemical range in the corresponding river basin.
(DOCX)

Figure S50 **The probability of Libellulidae being present in relation to the water temperature measured in Ecuador (red, solid), Ethiopia (green, dashed) and Vietnam (blue, dotdashed).** The gray-colored ends of the response curves indicate extrapolation outside the observed physical-chemical range in the corresponding river basin.
(DOCX)

Figure S51 **The probability of Libellulidae being present in relation to the conductivity measured in Ecuador (red, solid), Ethiopia (green, dashed) and Vietnam (blue, dotdashed).** The gray-colored ends of the response curves indicate extrapolation outside the observed physical-chemical range in the corresponding river basin.
(DOCX)

Figure S52 **The probability of Libellulidae being present in relation to pH measured in Ecuador (red, solid), Ethiopia (green, dashed) and Vietnam (blue, dotdashed).** The gray-colored ends of the response curves indicate extrapolation outside the observed physical-chemical range in the corresponding river basin.
(DOCX)

Figure S53 **The probability of Libellulidae being present in relation to dissolved oxygen (DO) concentration measured in Ecuador (red, solid), Ethiopia (green, dashed) and Vietnam (blue, dotdashed).** The gray-colored ends of the response curves indicate extrapolation outside the observed physical-chemical range in the corresponding river basin.
(DOCX)

Figure S54 **The probability of Leptophlebiidae being present in relation to stream velocity measured in Ecuador (red, solid), Ethiopia (green, dashed) and Vietnam (blue, dotdashed).** The gray-colored ends of the response curves indicate extrapolation outside the observed physical-chemical range in the corresponding river basin.
(DOCX)

Figure S55 **The probability of Leptophlebiidae being present in relation to the water temperature measured in Ecuador (red, solid), Ethiopia (green, dashed) and Vietnam (blue, dotdashed).** The gray-colored ends of the response curves indicate extrapolation outside the observed physical-chemical range in the corresponding river basin.
(DOCX)

Figure S56 **The probability of Leptophlebiidae being present in relation to the conductivity measured in Ecuador (red, solid), Ethiopia (green, dashed) and Vietnam (blue, dotdashed).** The gray-colored ends of the response curves indicate extrapolation outside the observed physical-chemical range in the corresponding river basin.
(DOCX)

Figure S57 **The probability of Leptophlebiidae being present in relation to pH measured in Ecuador (red, solid), Ethiopia (green, dashed) and Vietnam (blue, dotdashed).** The gray-colored ends of the response curves indicate extrapolation outside the observed physical-chemical range in the corresponding river basin.
(DOCX)

Figure S58 **The probability of Leptophlebiidae being present in relation to dissolved oxygen (DO) concentration measured in Ecuador (red, solid), Ethiopia (green, dashed) and Vietnam (blue, dotdashed).** The gray-colored ends of the response curves indicate extrapolation outside the observed physical-chemical range in the corresponding river basin.
(DOCX)

Table S1 **Seasonal differences per physical-chemical variable and per country.** A p-value less than 0.05 demonstrates a significant difference between seasons for the mean physical-chemical variable that is considered for a specific country. Significant relations are indicated in bold.
(DOCX)

Table S2 **Country-wise differences per physical-chemical variable and per season.** A p-value less than 0.05 demonstrates a significant difference between countries for the mean physical-chemical variable that is considered for a specific season. Significant relations are indicated in bold.
(DOCX)
