# Supplementary figures and images for: Comparison of the Abiotic Preferences of Macroinvertebrates in Tropical River Basins
Source: PLoS One. 2014 Oct 3;9(10):e108898. doi: 10.1371/journal.pone.0108898 (PMC4184827; doi:10.1371/journal.pone.0108898)

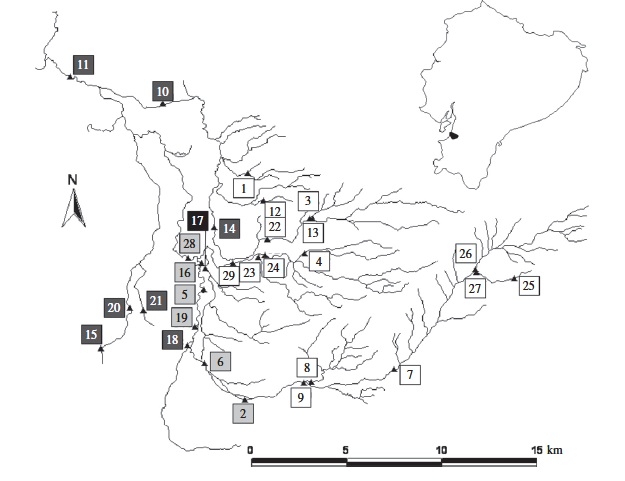

Supplement: Figure S1 — Sampling sites in the Chaguana river basin in Ecuador. (DOCX) [file pone.0108898.s001.docx]

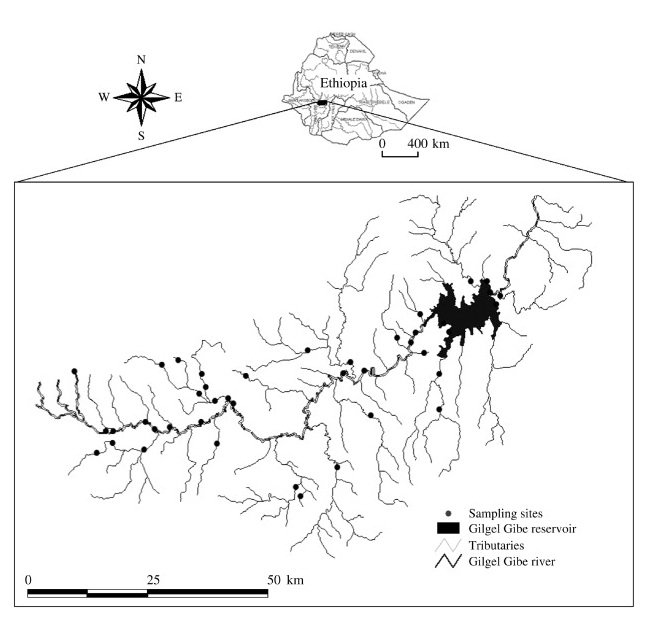

Supplement: Figure S2 — Sampling sites in the Gilgel Gibe river basin in Ethiopia. (DOCX) [file pone.0108898.s002.docx]

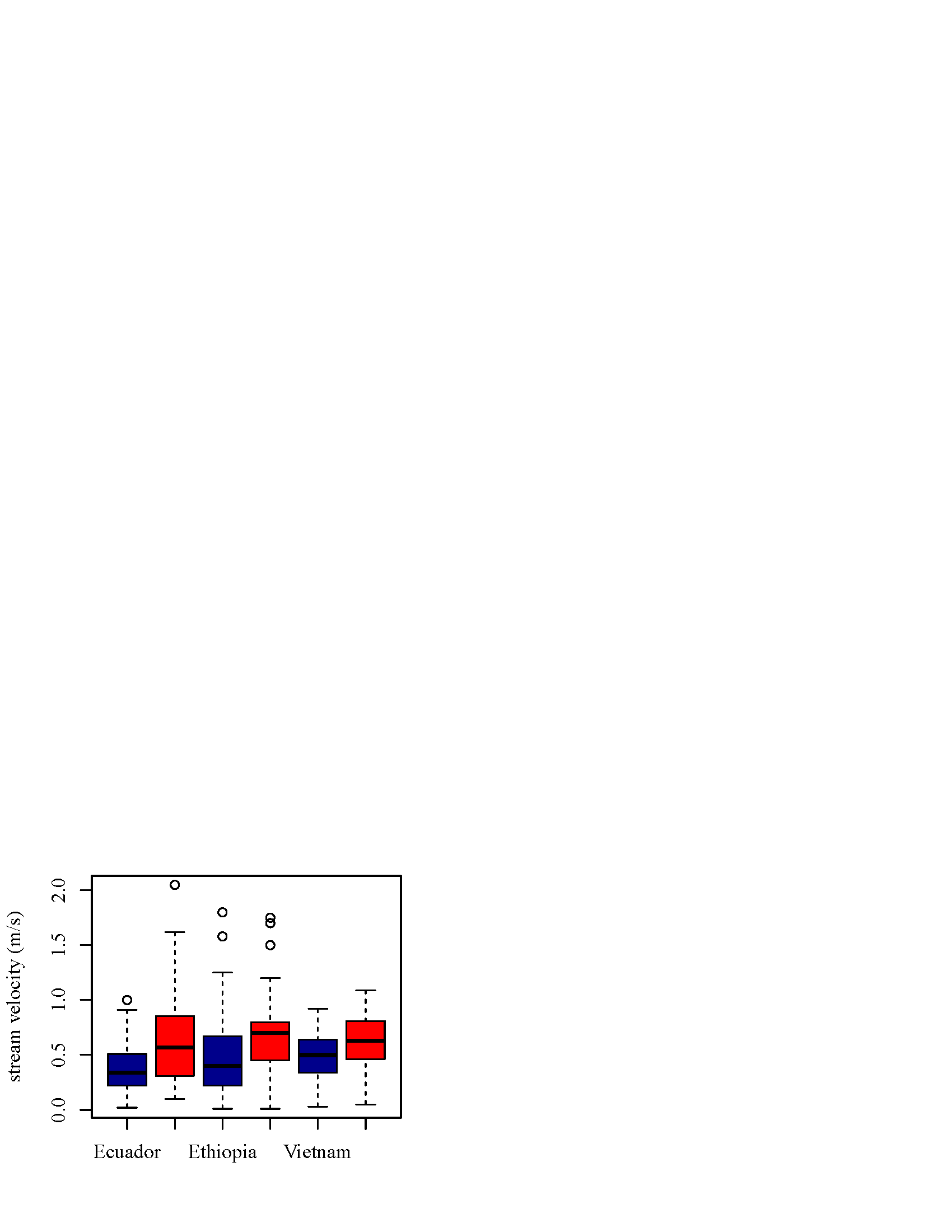

Supplement: Figure S4 — Boxplots showing the seasonality (dry (blue) and wet (red) season) of the stream velocity. (DOCX) [file pone.0108898.s004.docx]

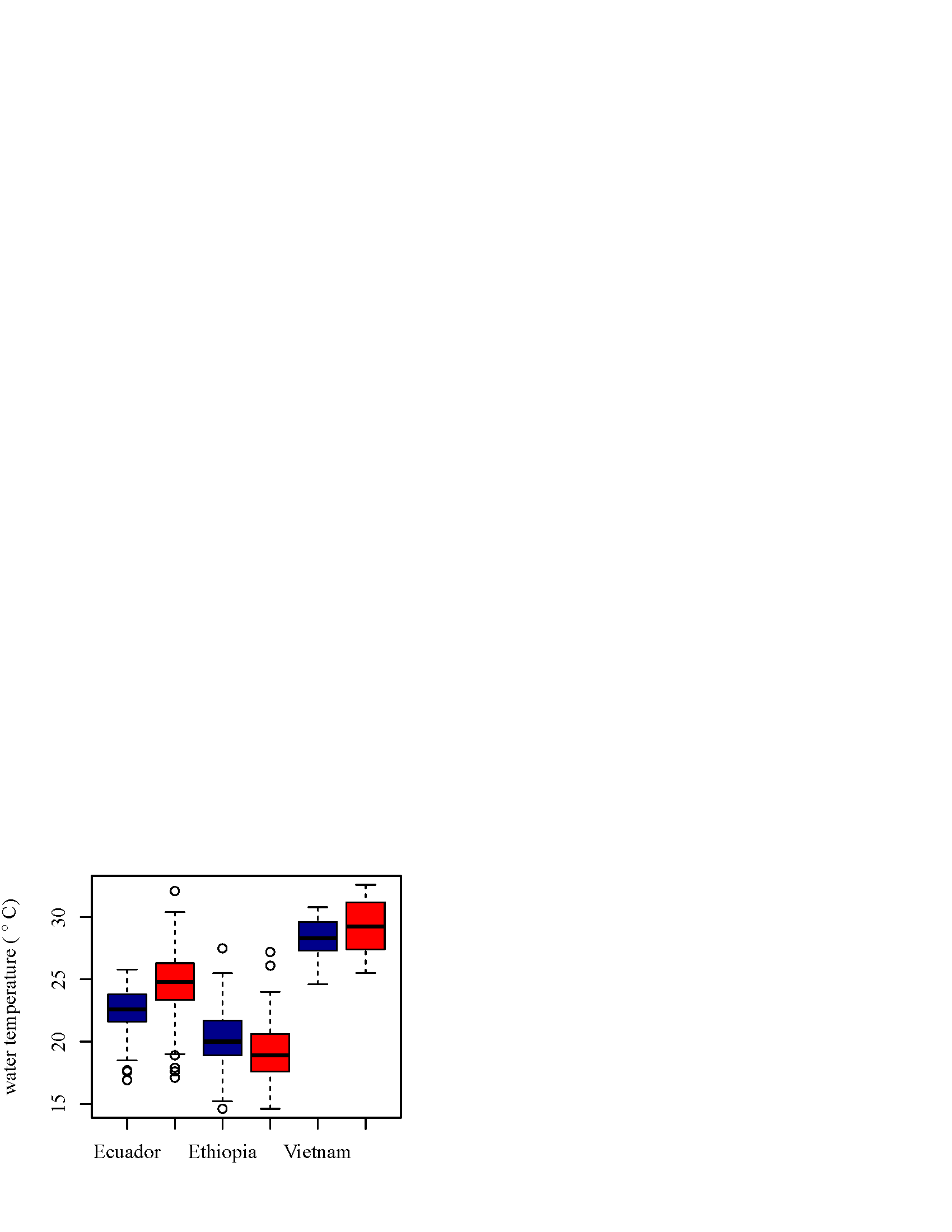

Supplement: Figure S5 — Boxplots showing the seasonality (dry (blue) and wet (red) season) of the water temperature. (DOCX) [file pone.0108898.s005.docx]

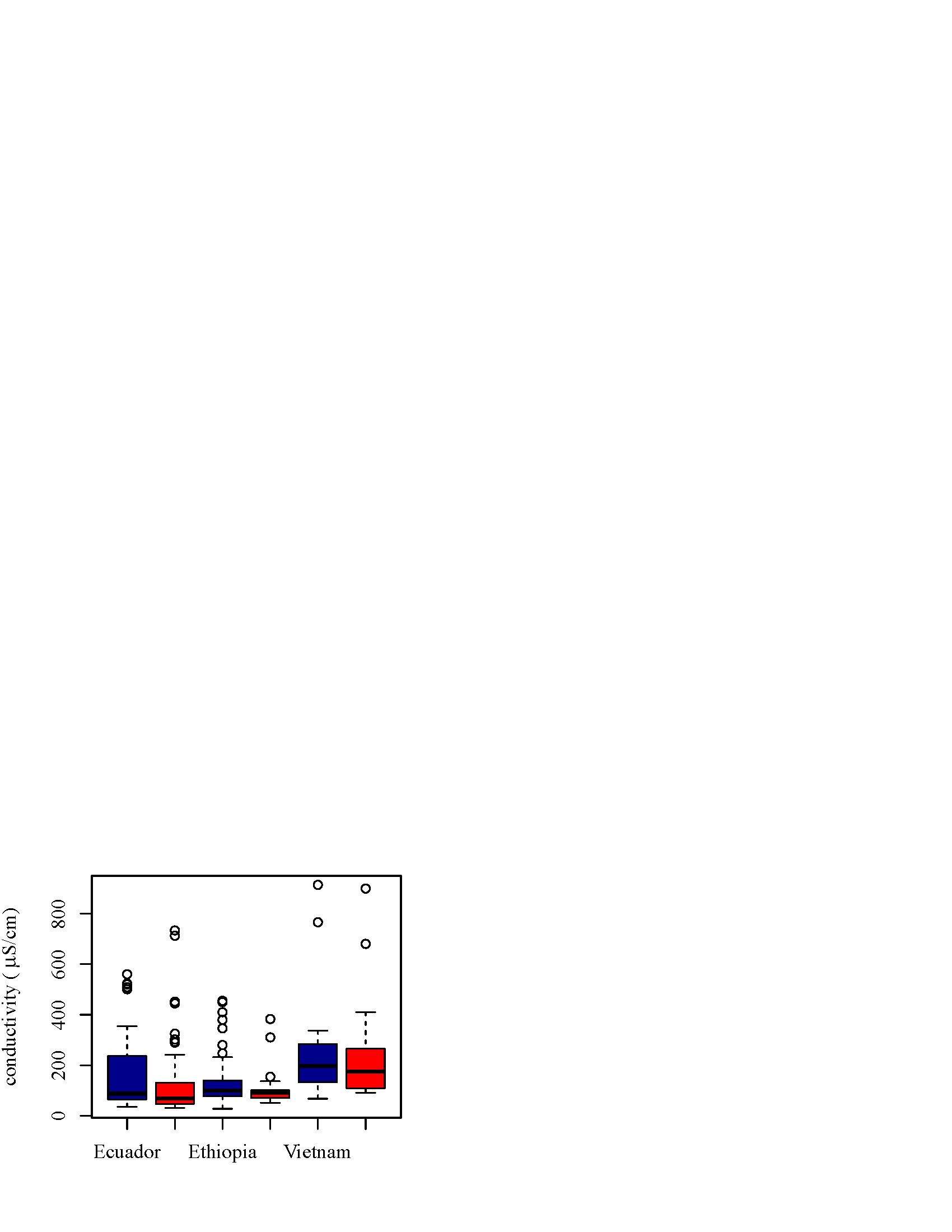

Supplement: Figure S6 — Boxplots showing the seasonality (dry (blue) and wet (red) season) of the conductivity. (DOCX) [file pone.0108898.s006.docx]

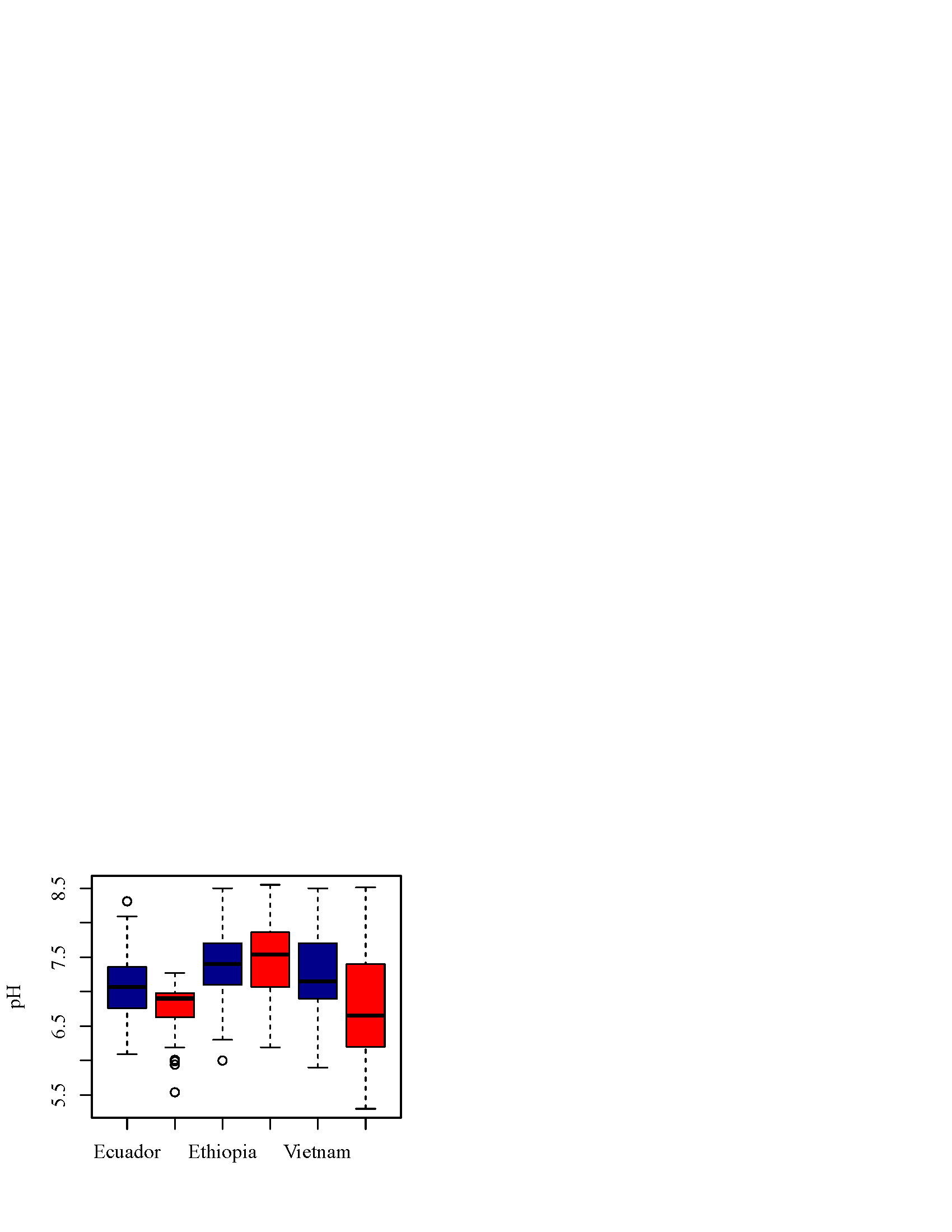

Supplement: Figure S7 — Boxplots showing the seasonality (dry (blue) and wet (red) season) of the pH. (DOCX) [file pone.0108898.s007.docx]

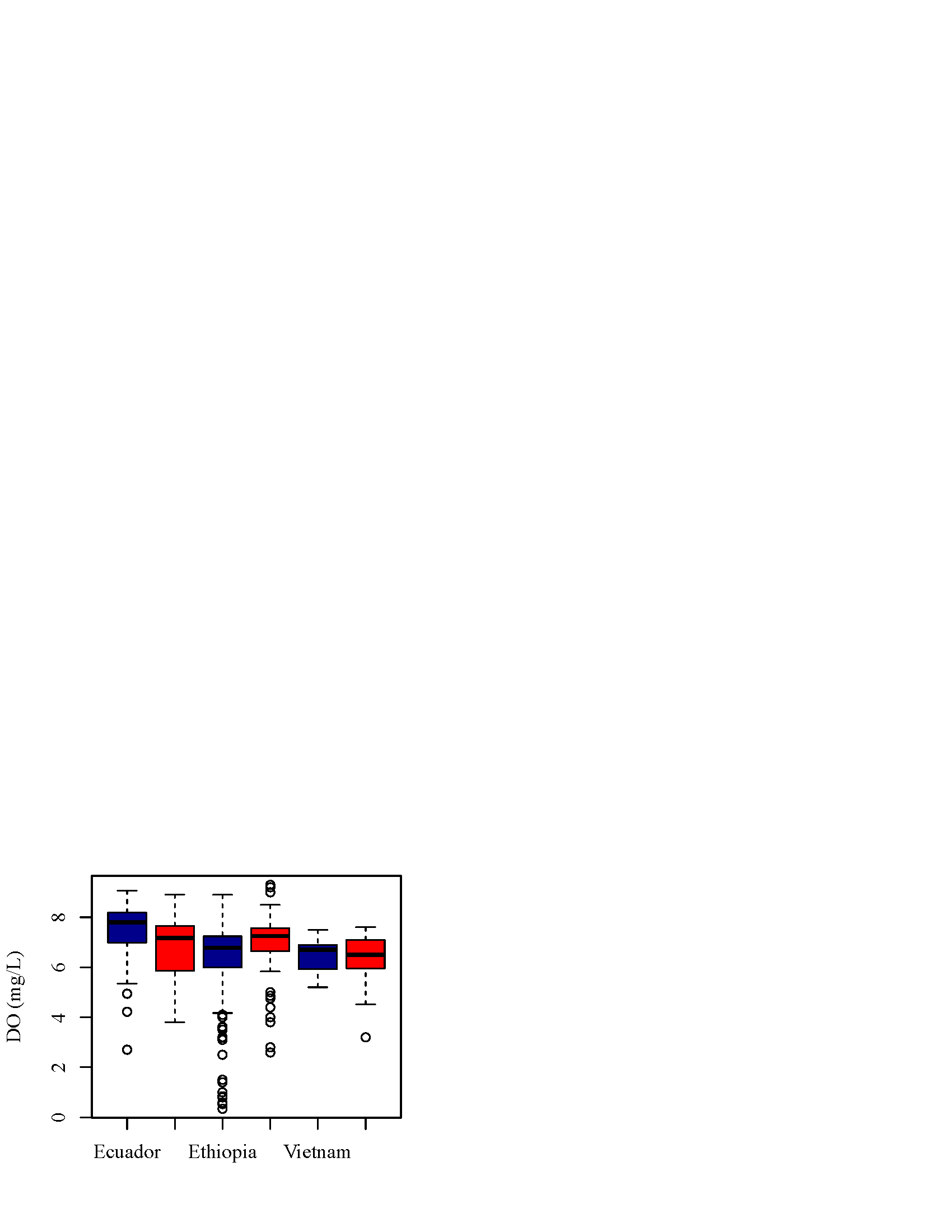

Supplement: Figure S8 — Boxplots showing the seasonality (dry (blue) and wet (red) season) of the dissolved oxygen. (DOCX) [file pone.0108898.s008.docx]

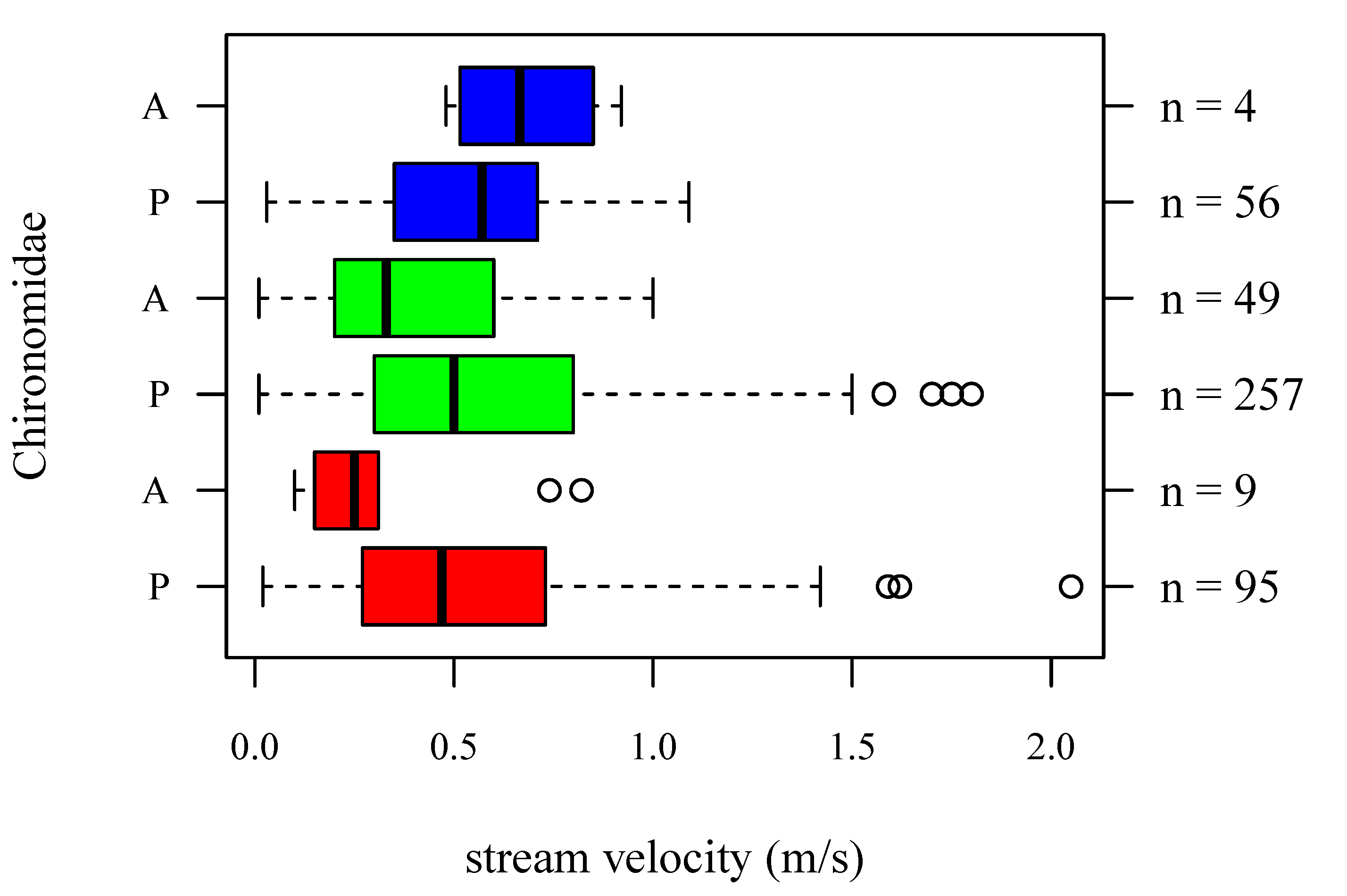

Supplement: Figure S9 — Boxplots indicate the observed stream velocity in Ecuador (red), Ethiopia (green) and Vietnam (blue) at which Chironomidae are found to be present (denoted by P on the left axis) and absent (denoted by A on the left axis). The sample sizes per boxplot are shown on the right axis. (DOCX) [file pone.0108898.s009.docx]

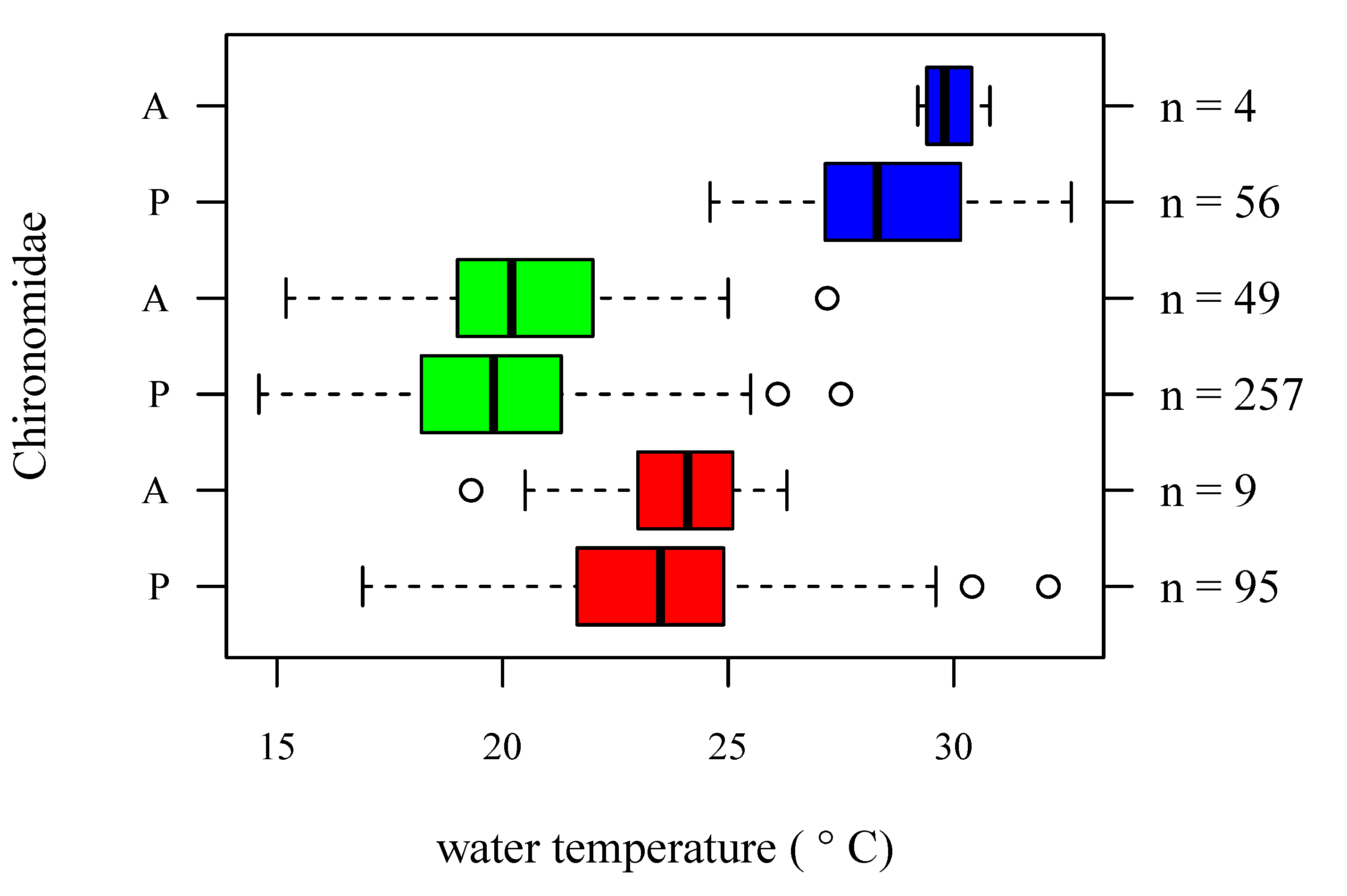

Supplement: Figure S10 — Boxplots indicate the observed water temperature in Ecuador (red), Ethiopia (green) and Vietnam (blue) at which Chironomidae are found to be present (denoted by P on the left axis) and absent (denoted by A on the left axis). The sample sizes per boxplot are shown on the right axis. (DOCX) [file pone.0108898.s010.docx]

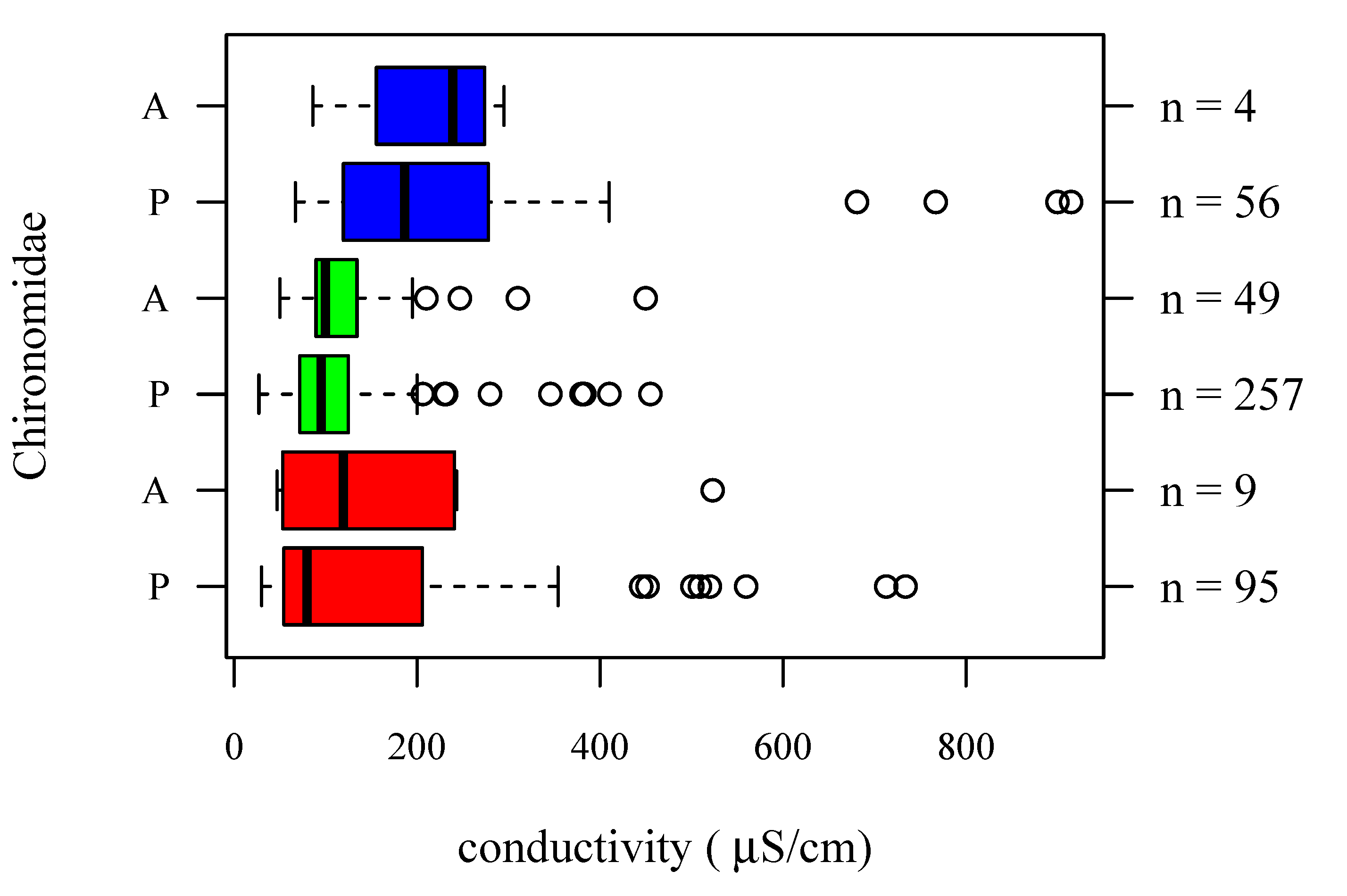

Supplement: Figure S11 — Boxplots indicate the observed conductivity in Ecuador (red), Ethiopia (green) and Vietnam (blue) at which Chironomidae are found to be present (denoted by P on the left axis) and absent (denoted by A on the left axis). The sample sizes per boxplot are shown on the right axis. (DOCX) [file pone.0108898.s011.docx]

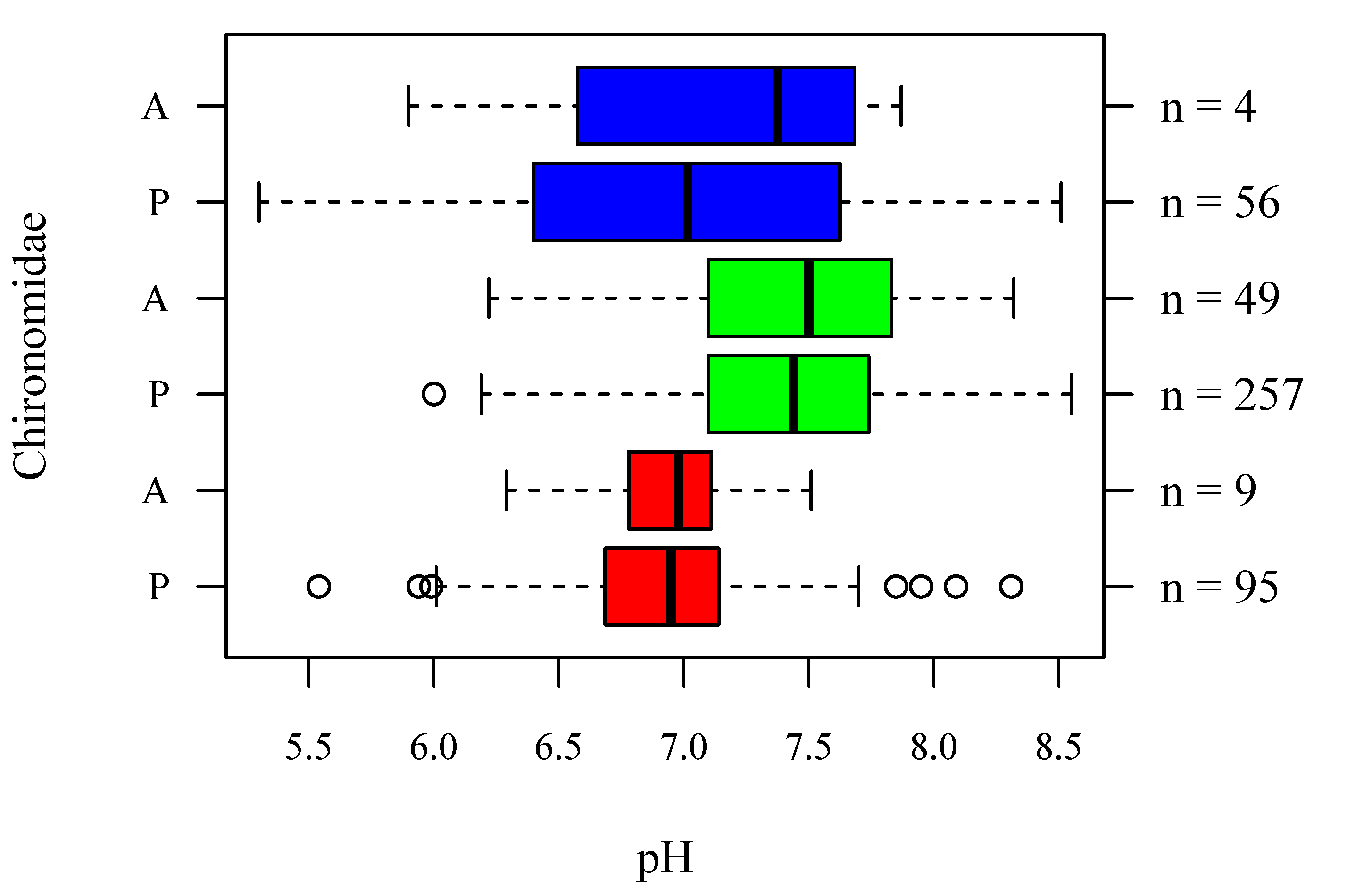

Supplement: Figure S12 — Boxplots indicate the observed pH in Ecuador (red), Ethiopia (green) and Vietnam (blue) at which Chironomidae are found to be present (denoted by P on the left axis) and absent (denoted by A on the left axis). The sample sizes per boxplot are shown on the right axis. (DOCX) [file pone.0108898.s012.docx]

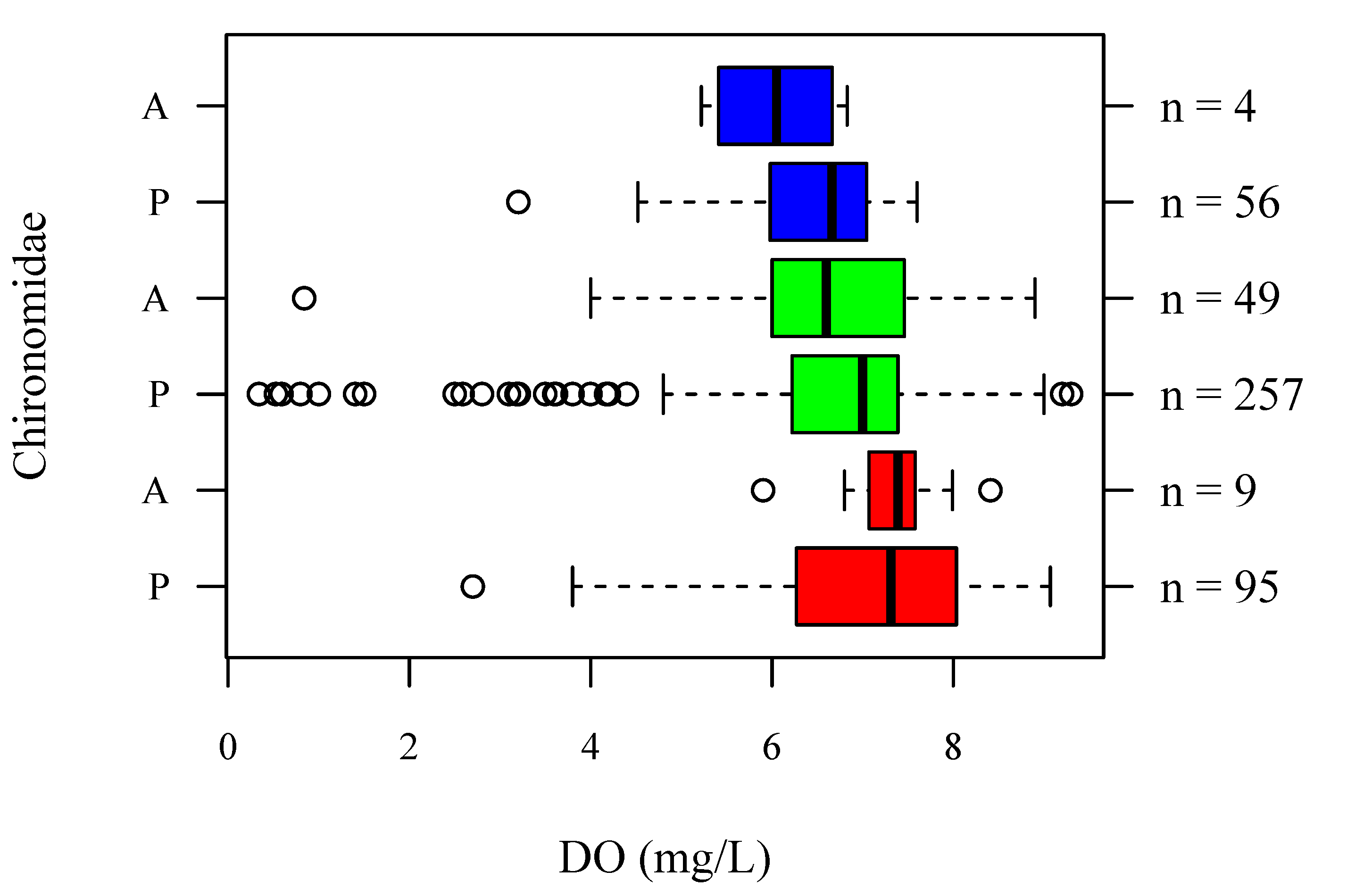

Supplement: Figure S13 — Boxplots indicate the observed DO concentrations in Ecuador (red), Ethiopia (green) and Vietnam (blue) at which Chironomidae are found to be present (denoted by P on the left axis) and absent (denoted by A on the left axis). The sample sizes per boxplot are shown on the right axis. (DOCX) [file pone.0108898.s013.docx]

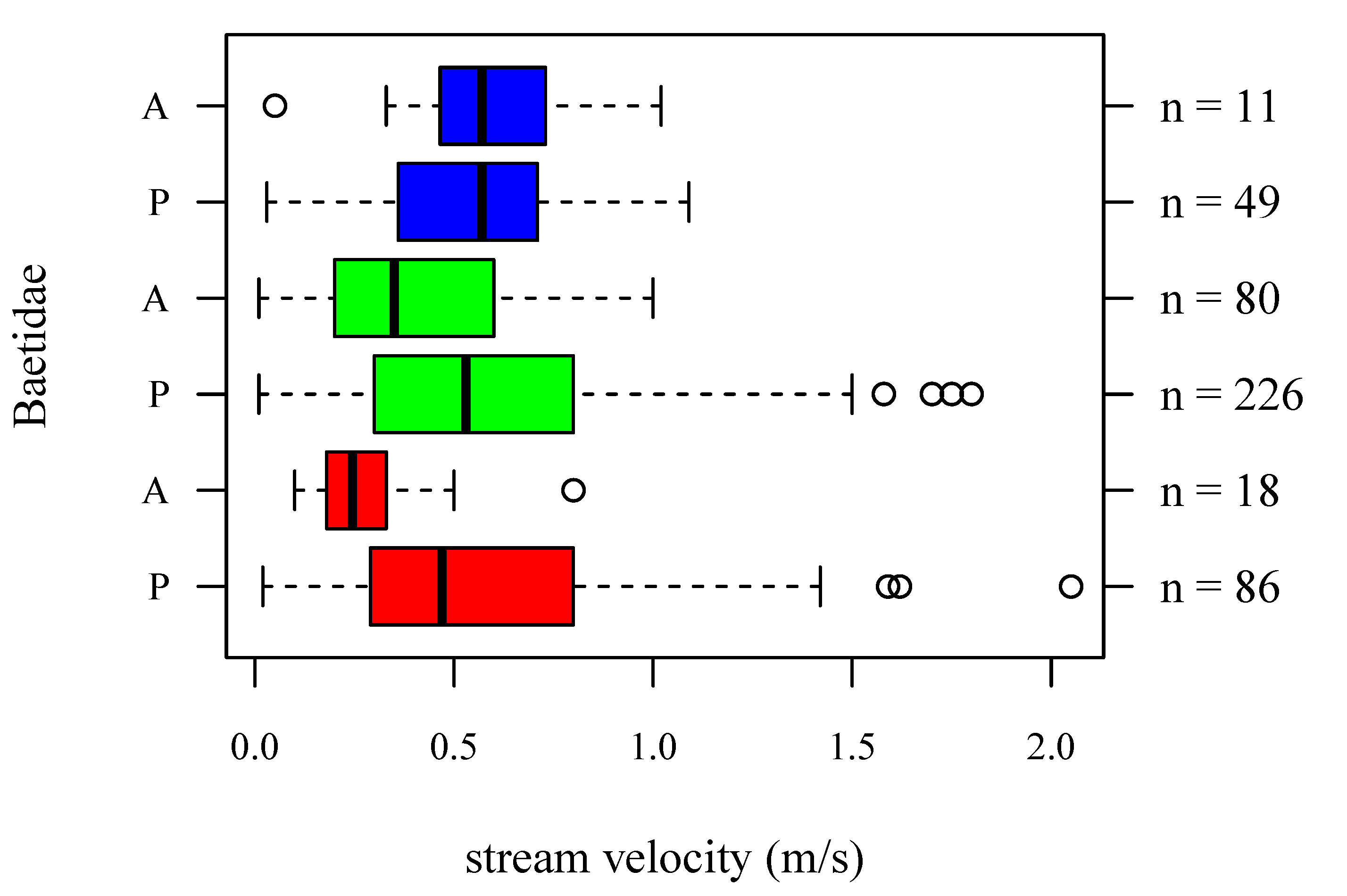

Supplement: Figure S14 — Boxplots indicate the observed stream velocity in Ecuador (red), Ethiopia (green) and Vietnam (blue) at which Baetidae are found to be present (denoted by P on the left axis) and absent (denoted by A on the left axis). The sample sizes per boxplot are shown on the right axis. (DOCX) [file pone.0108898.s014.docx]

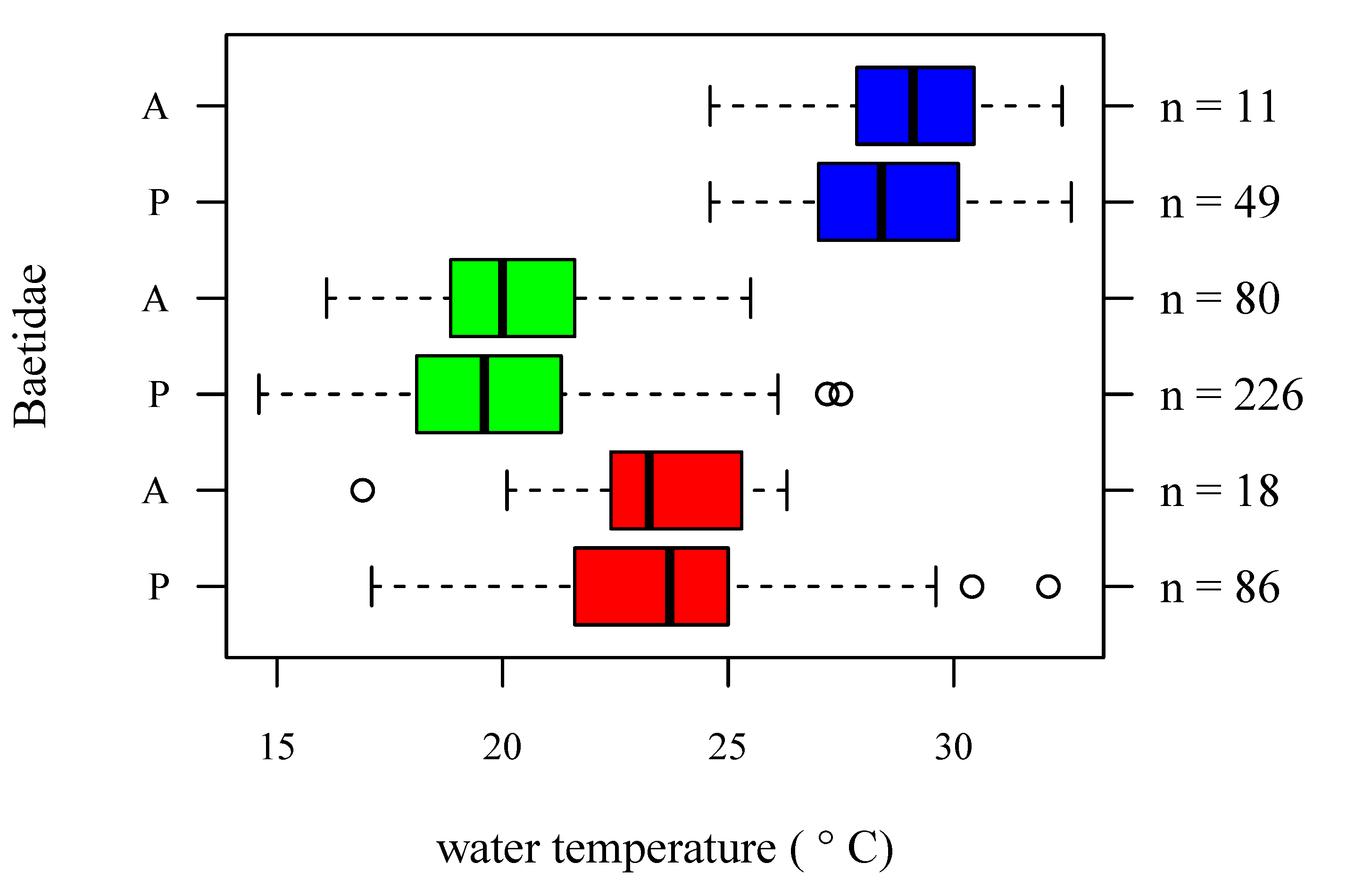

Supplement: Figure S15 — Boxplots indicate the observed water temperature in Ecuador (red), Ethiopia (green) and Vietnam (blue) at which Baetidae are found to be present (denoted by P on the left axis) and absent (denoted by A on the left axis). The sample sizes per boxplot are shown on the right axis. (DOCX) [file pone.0108898.s015.docx]

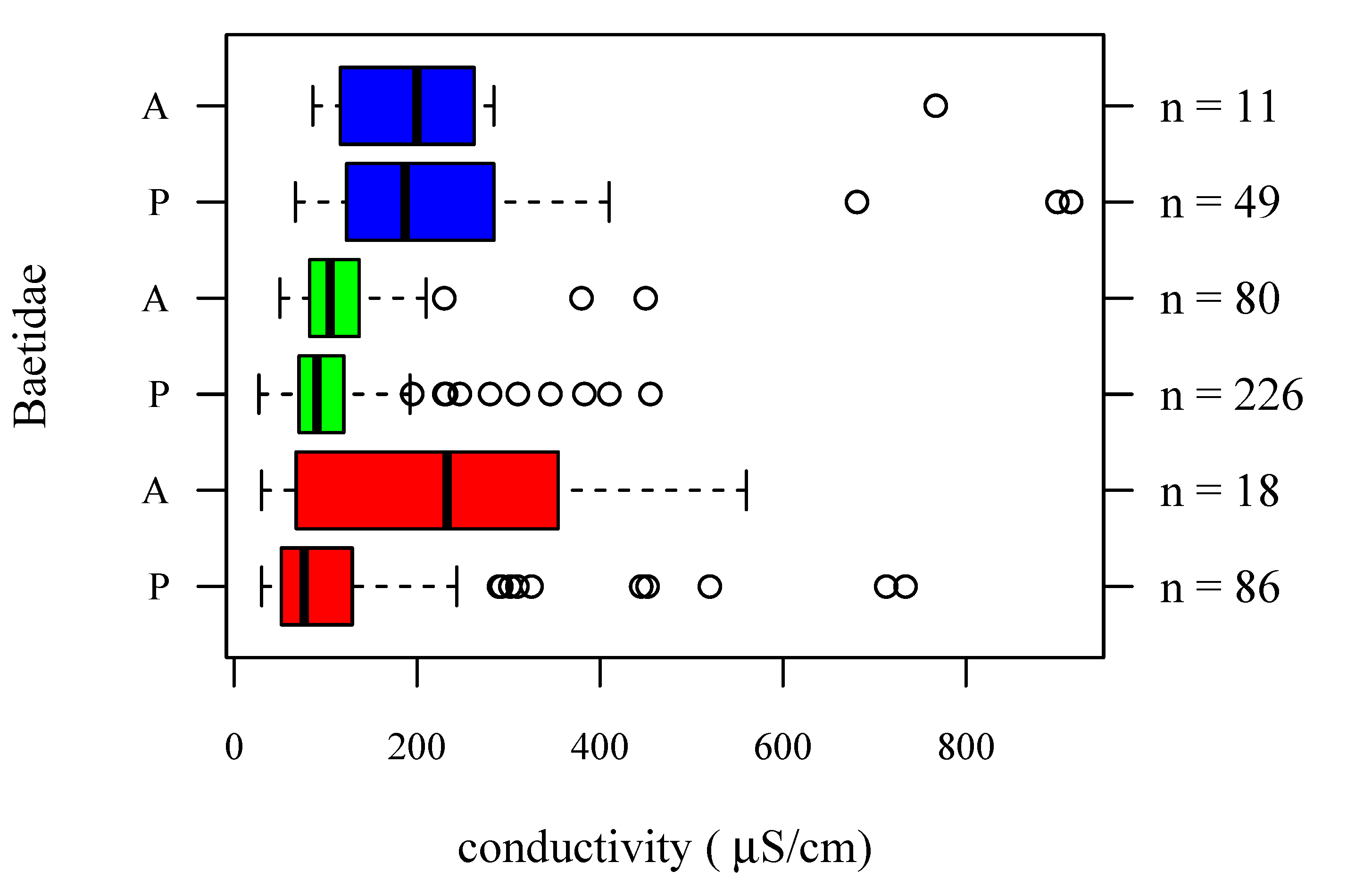

Supplement: Figure S16 — Boxplots indicate the observed conductivity in Ecuador (red), Ethiopia (green) and Vietnam (blue) at which Baetidae are found to be present (denoted by P on the left axis) and absent (denoted by A on the left axis). The sample sizes per boxplot are shown on the right axis. (DOCX) [file pone.0108898.s016.docx]

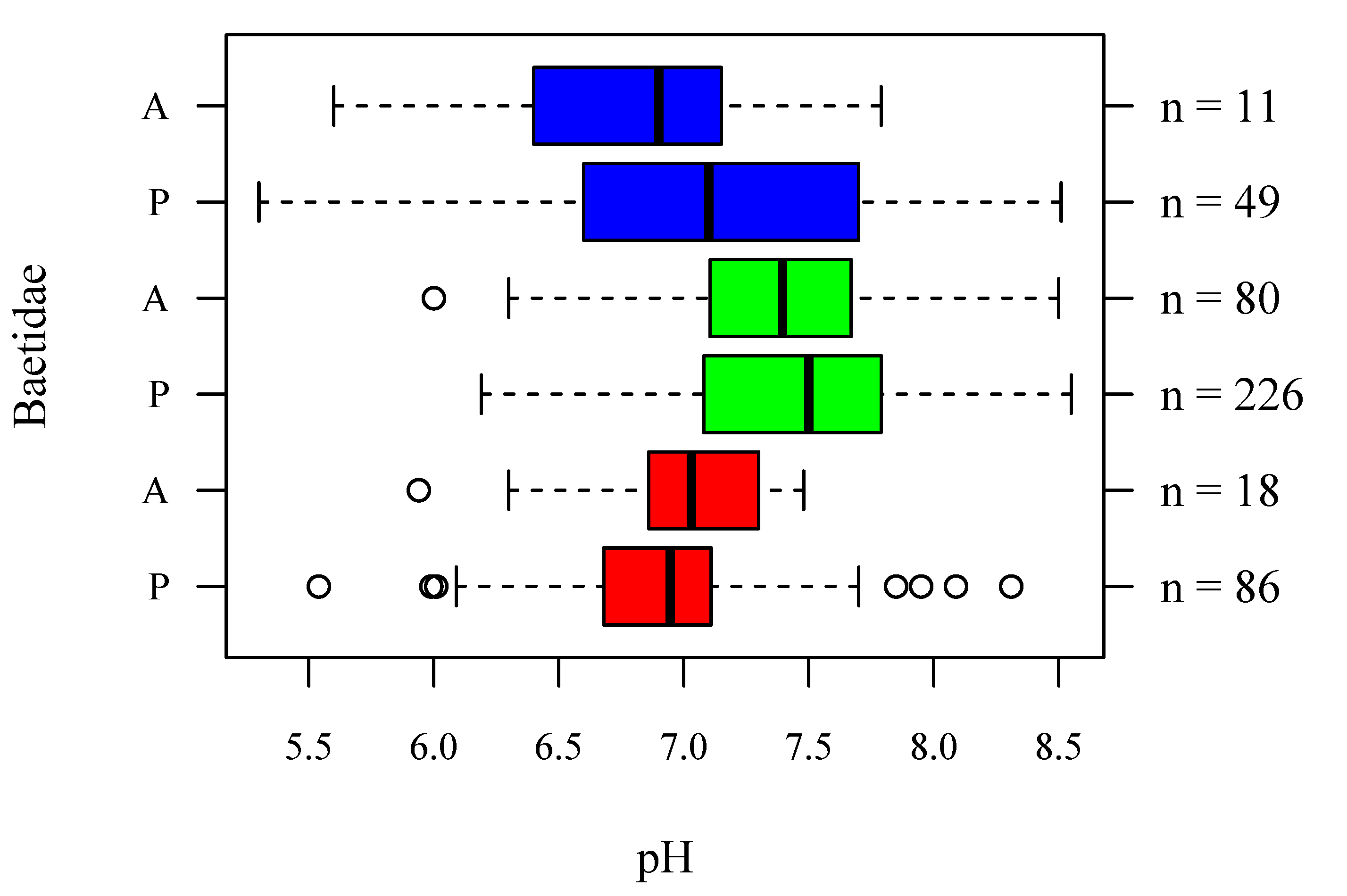

Supplement: Figure S17 — Boxplots indicate the observed pH in Ecuador (red), Ethiopia (green) and Vietnam (blue) at which Baetidae are found to be present (denoted by P on the left axis) and absent (denoted by A on the left axis). The sample sizes per boxplot are shown on the right axis. (DOCX) [file pone.0108898.s017.docx]

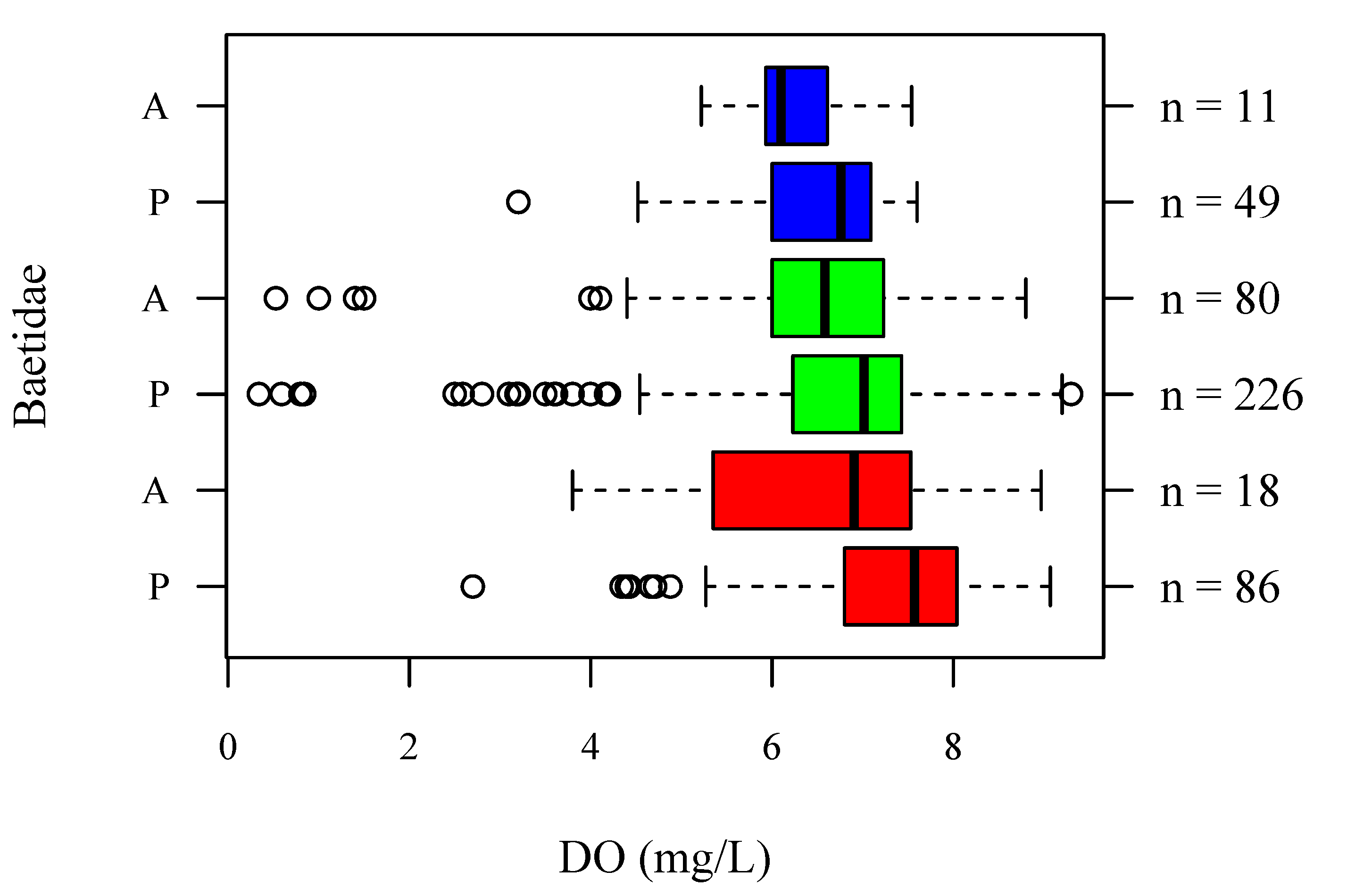

Supplement: Figure S18 — Boxplots indicate the observed DO concentrations in Ecuador (red), Ethiopia (green) and Vietnam (blue) at which Baetidae are found to be present (denoted by P on the left axis) and absent (denoted by A on the left axis). The sample sizes per boxplot are shown on the right axis. (DOCX) [file pone.0108898.s018.docx]

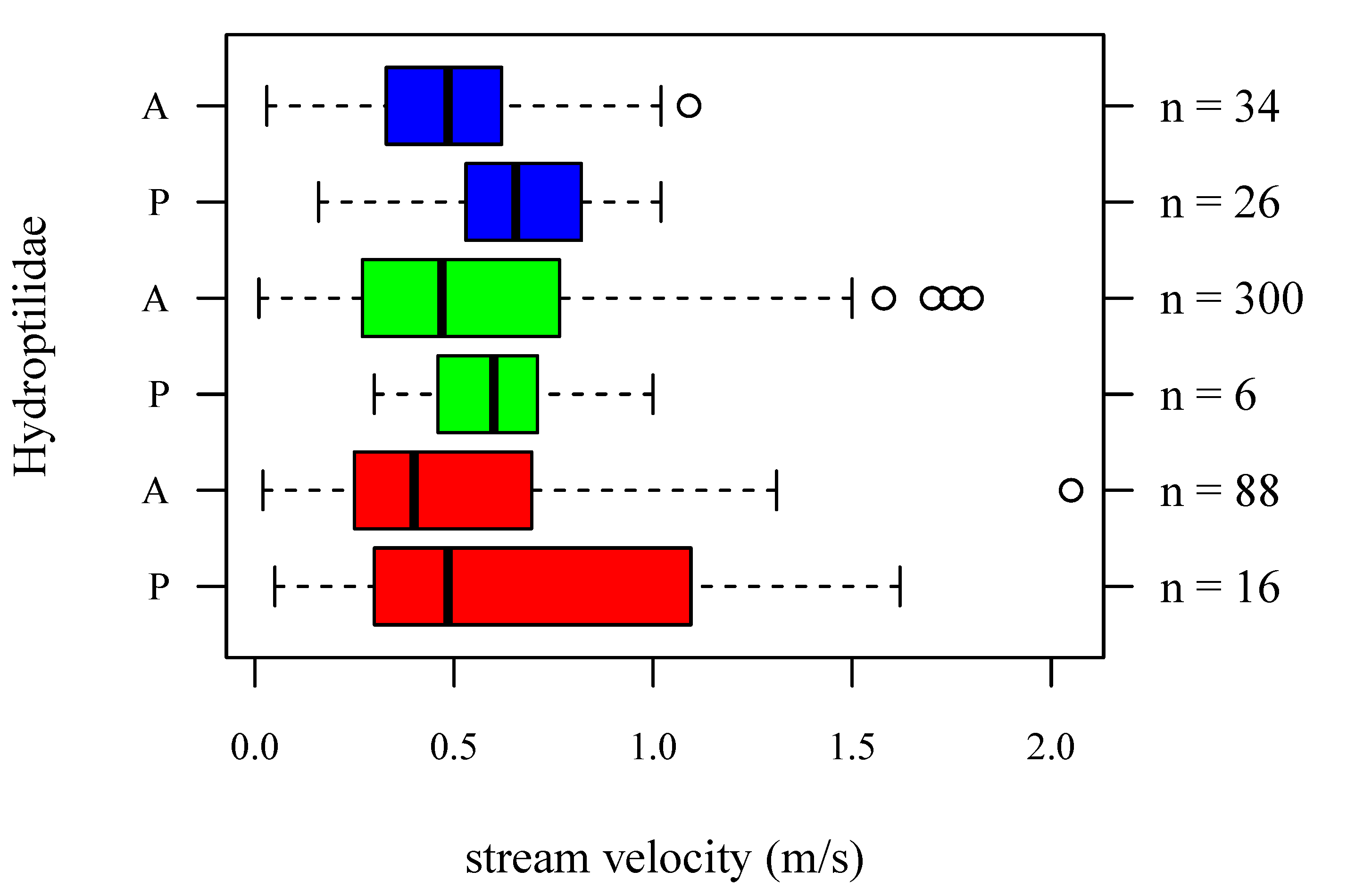

Supplement: Figure S19 — Boxplots indicate the observed stream velocity in Ecuador (red), Ethiopia (green) and Vietnam (blue) at which Hydroptilidae are found to be present (denoted by P on the left axis) and absent (denoted by A on the left axis). (DOCX) [file pone.0108898.s019.docx]

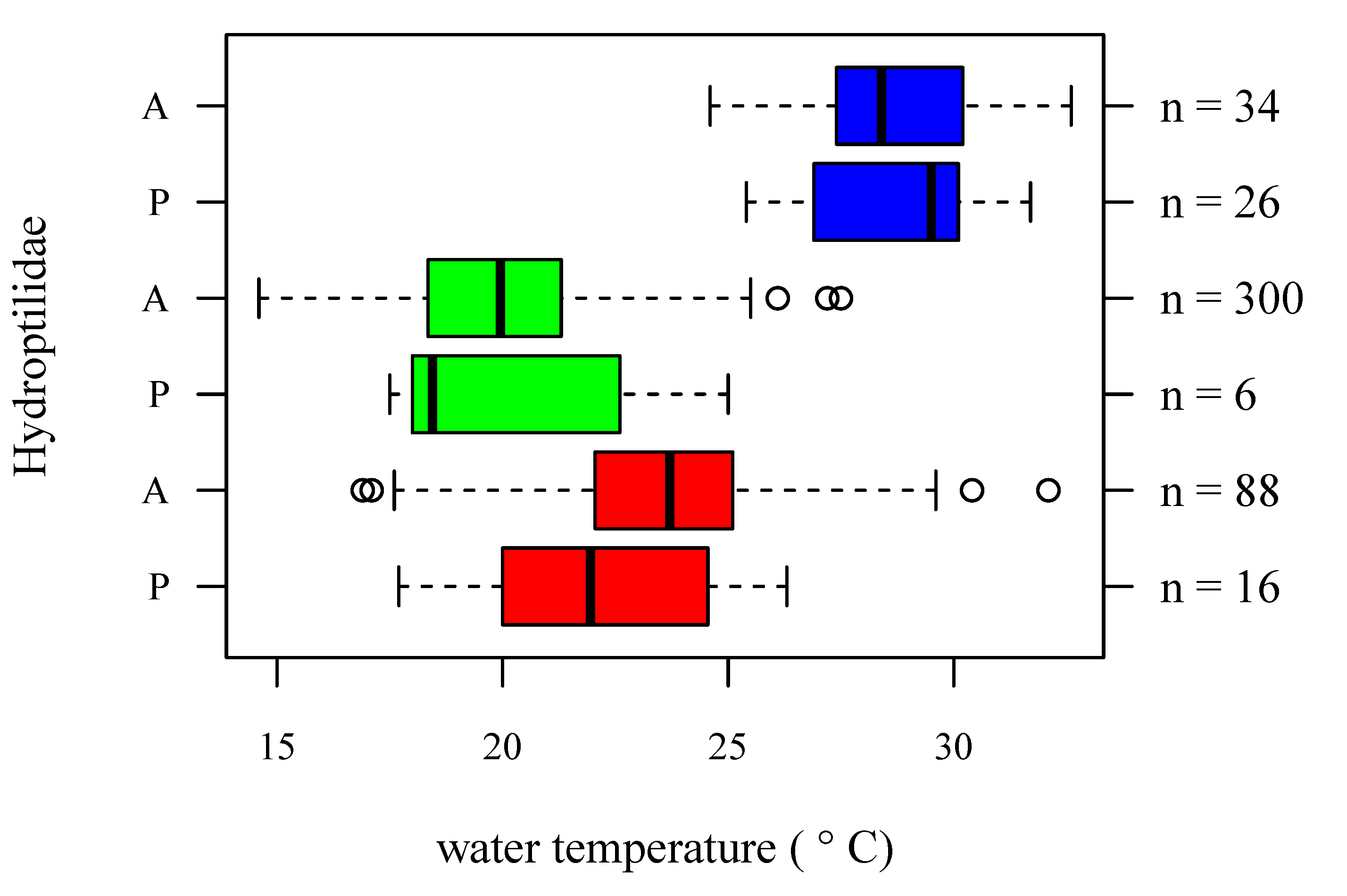

Supplement: Figure S20 — Boxplots indicate the observed water temperature in Ecuador (red), Ethiopia (green) and Vietnam (blue) at which Hydroptilidae are found to be present (denoted by P on the left axis) and absent (denoted by A on the left axis). The sample sizes per boxplot are shown on the right axis. (DOCX) [file pone.0108898.s020.docx]

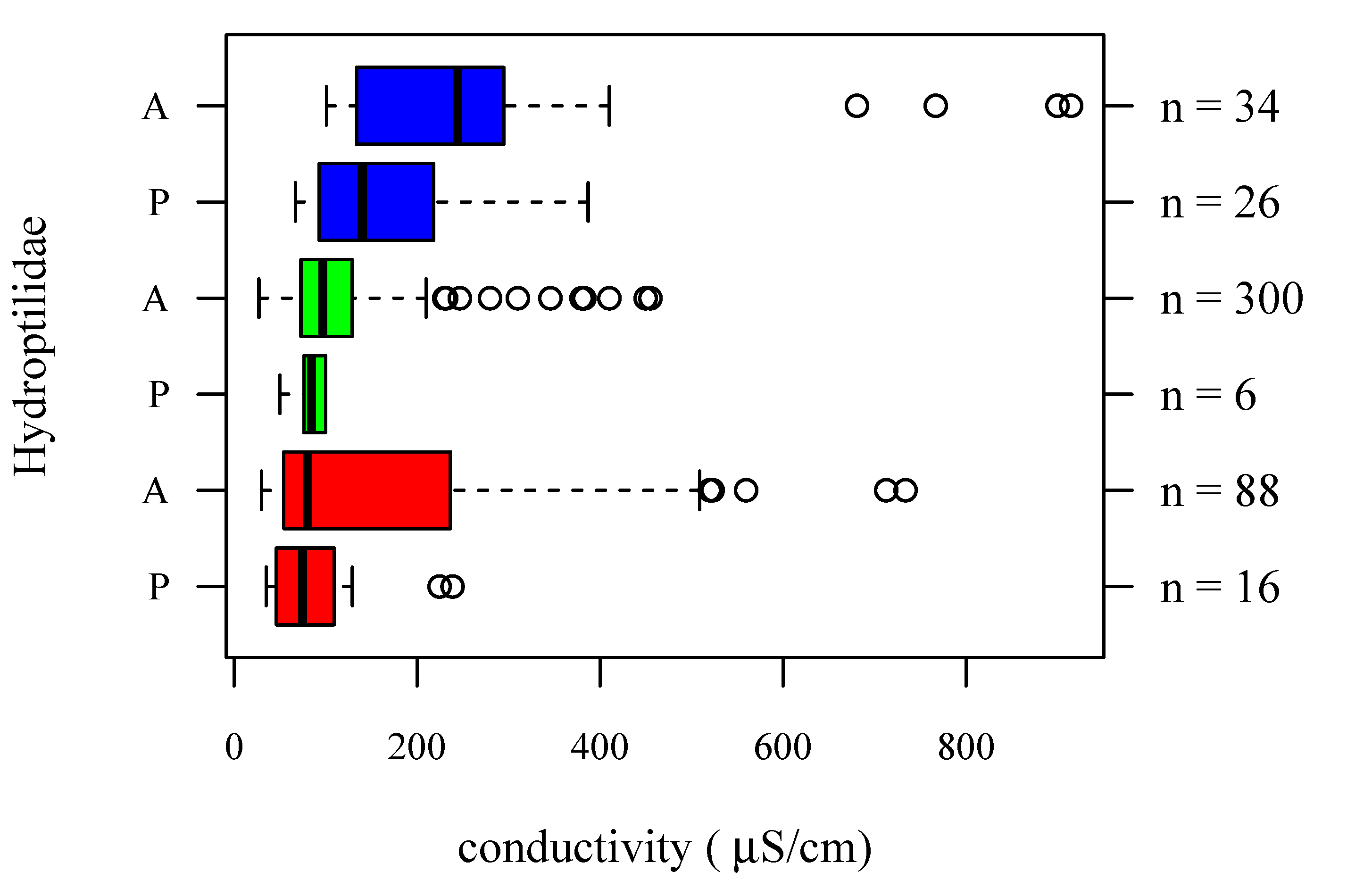

Supplement: Figure S21 — Boxplots indicate the observed conductivity in Ecuador (red), Ethiopia (green) and Vietnam (blue) at which Hydroptilidae are found to be present (denoted by P on the left axis) and absent (denoted by A on the left axis). The sample sizes per boxplot are shown on the right axis. (DOCX) [file pone.0108898.s021.docx]

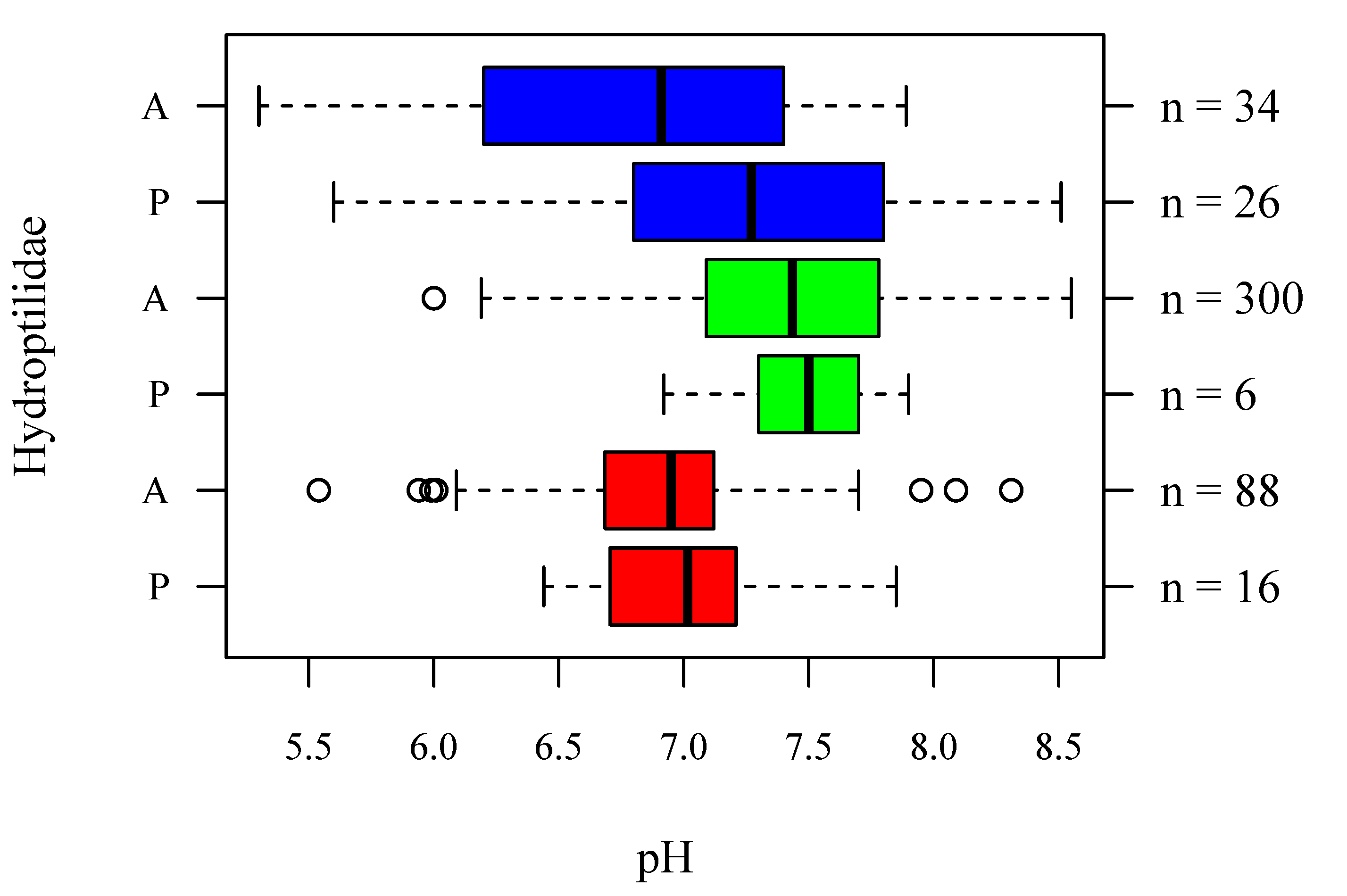

Supplement: Figure S22 — Bottom: Boxplots indicate the observed pH in Ecuador (red), Ethiopia (green) and Vietnam (blue) at which Hydroptilidae are found to be present (denoted by P on the left axis) and absent (denoted by A on the left axis). (DOCX) [file pone.0108898.s022.docx]

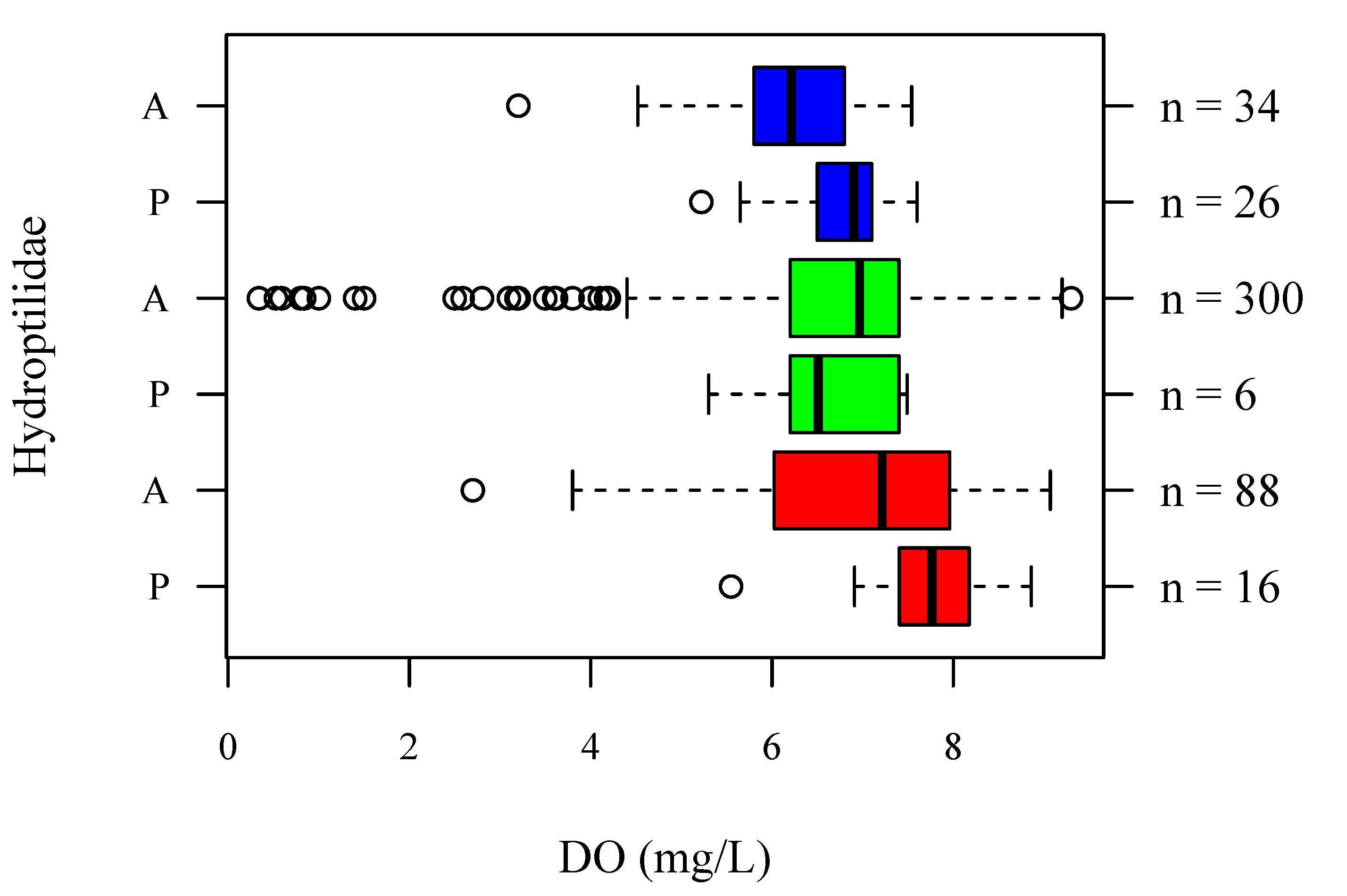

Supplement: Figure S23 — Boxplots indicate the observed DO concentrations in Ecuador (red), Ethiopia (green) and Vietnam (blue) at which Hydroptilidae are found to be present (denoted by P on the left axis) and absent (denoted by A on the left axis). The sample sizes per boxplot are shown on the right axis. (DOCX) [file pone.0108898.s023.docx]

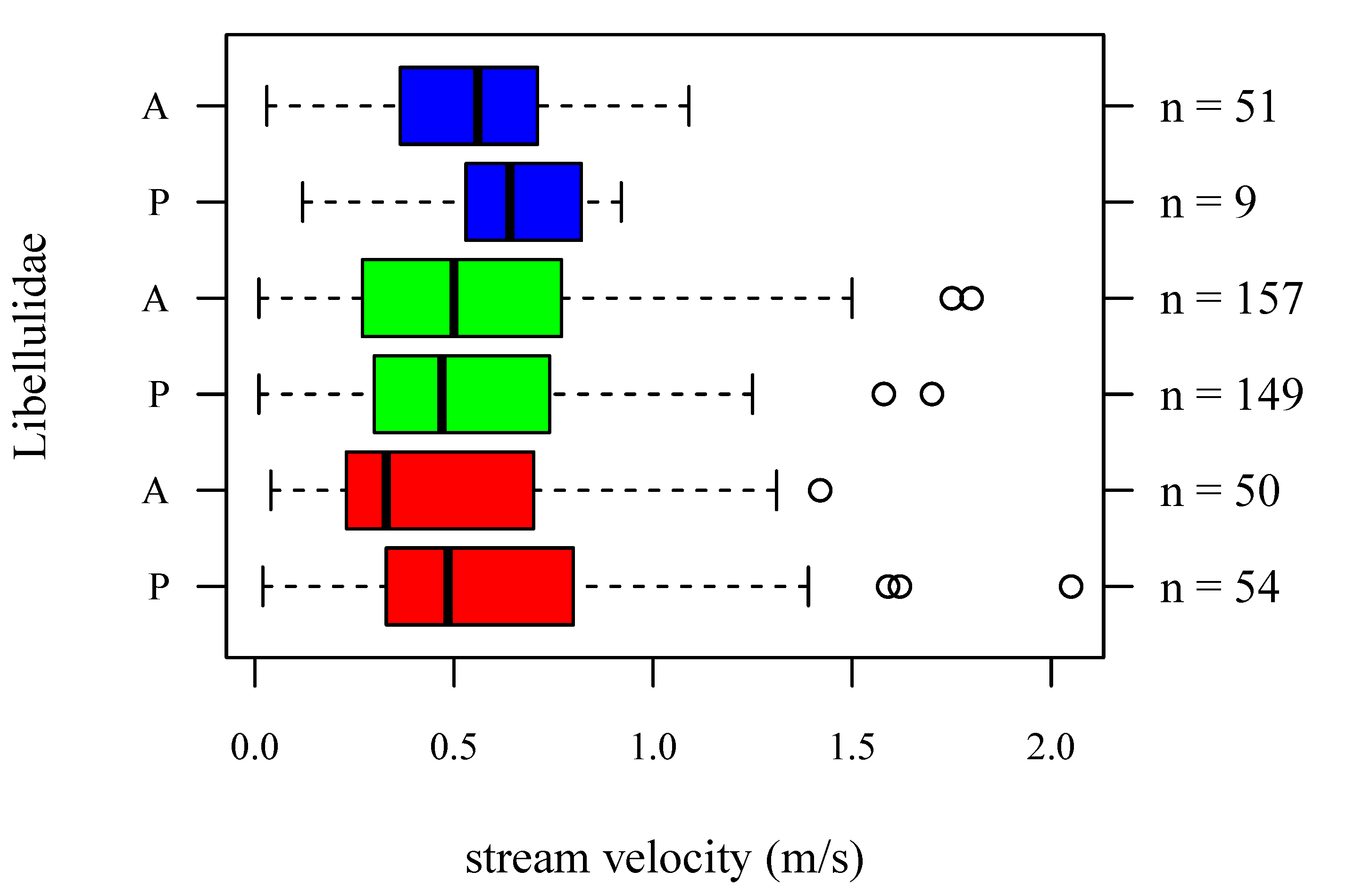

Supplement: Figure S24 — Boxplots indicate the observed stream velocity in Ecuador (red), Ethiopia (green) and Vietnam (blue) at which Libellulidae are found to be present (denoted by P on the left axis) and absent (denoted by A on the left axis). The sample sizes per boxplot are shown on the right axis. (DOCX) [file pone.0108898.s024.docx]

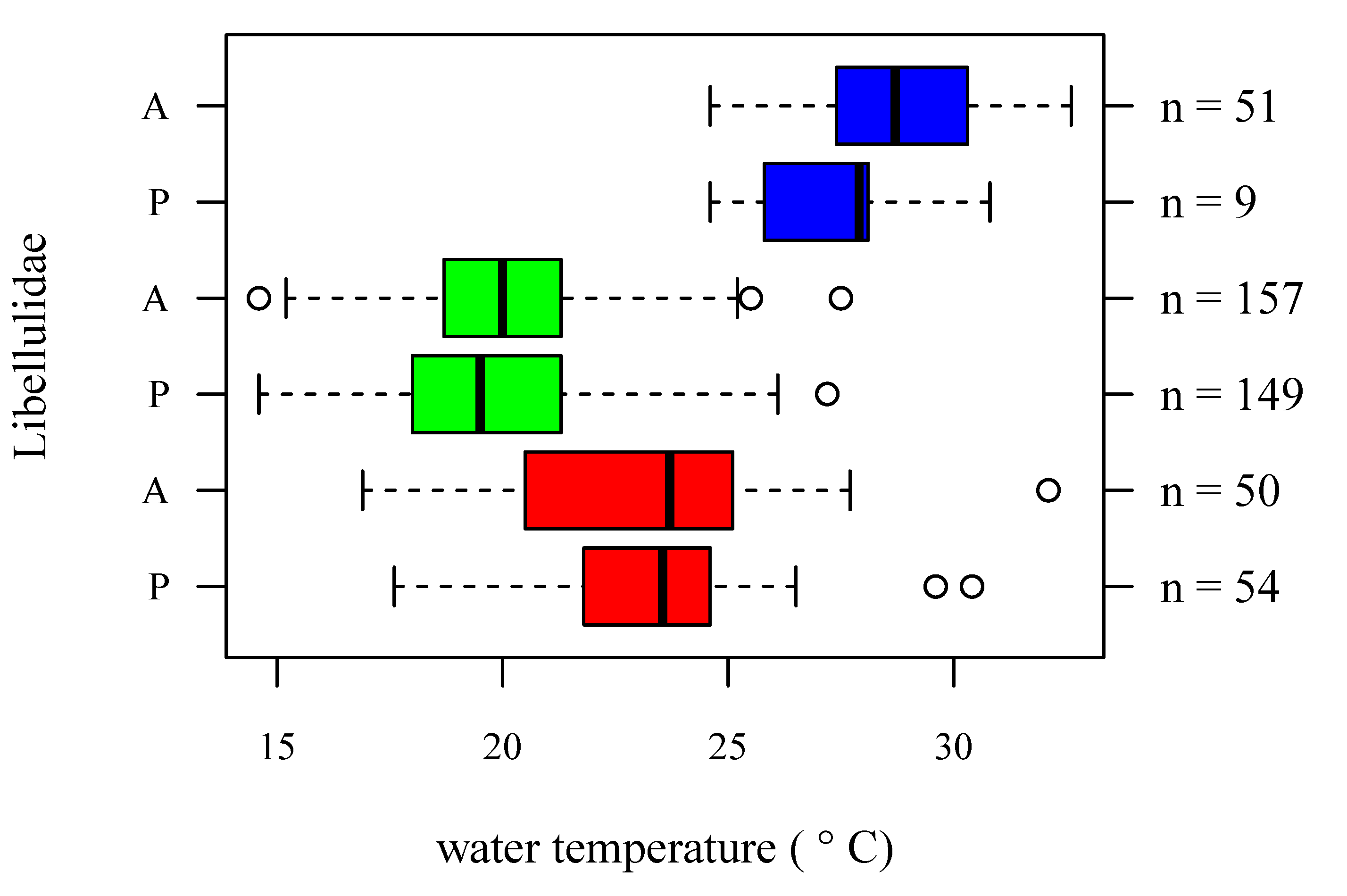

Supplement: Figure S25 — Boxplots indicate the observed water temperature in Ecuador (red), Ethiopia (green) and Vietnam (blue) at which Libellulidae are found to be present (denoted by P on the left axis) and absent (denoted by A on the left axis). The sample sizes per boxplot are shown on the right axis. (DOCX) [file pone.0108898.s025.docx]

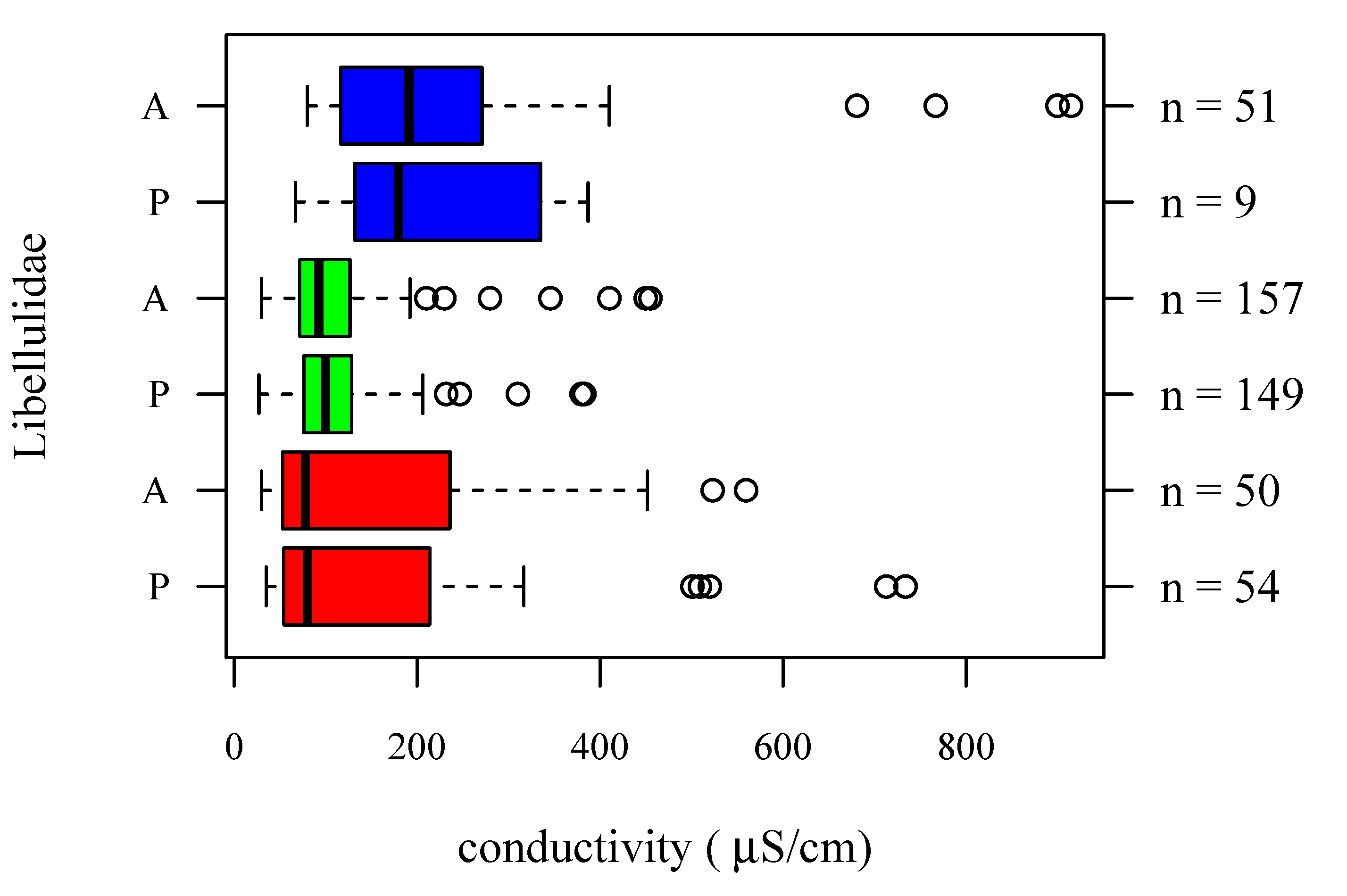

Supplement: Figure S26 — Boxplots indicate the observed conductivity in Ecuador (red), Ethiopia (green) and Vietnam (blue) at which Libellulidae are found to be present (denoted by P on the left axis) and absent (denoted by A on the left axis). The sample sizes per boxplot are shown on the right axis. (DOCX) [file pone.0108898.s026.docx]

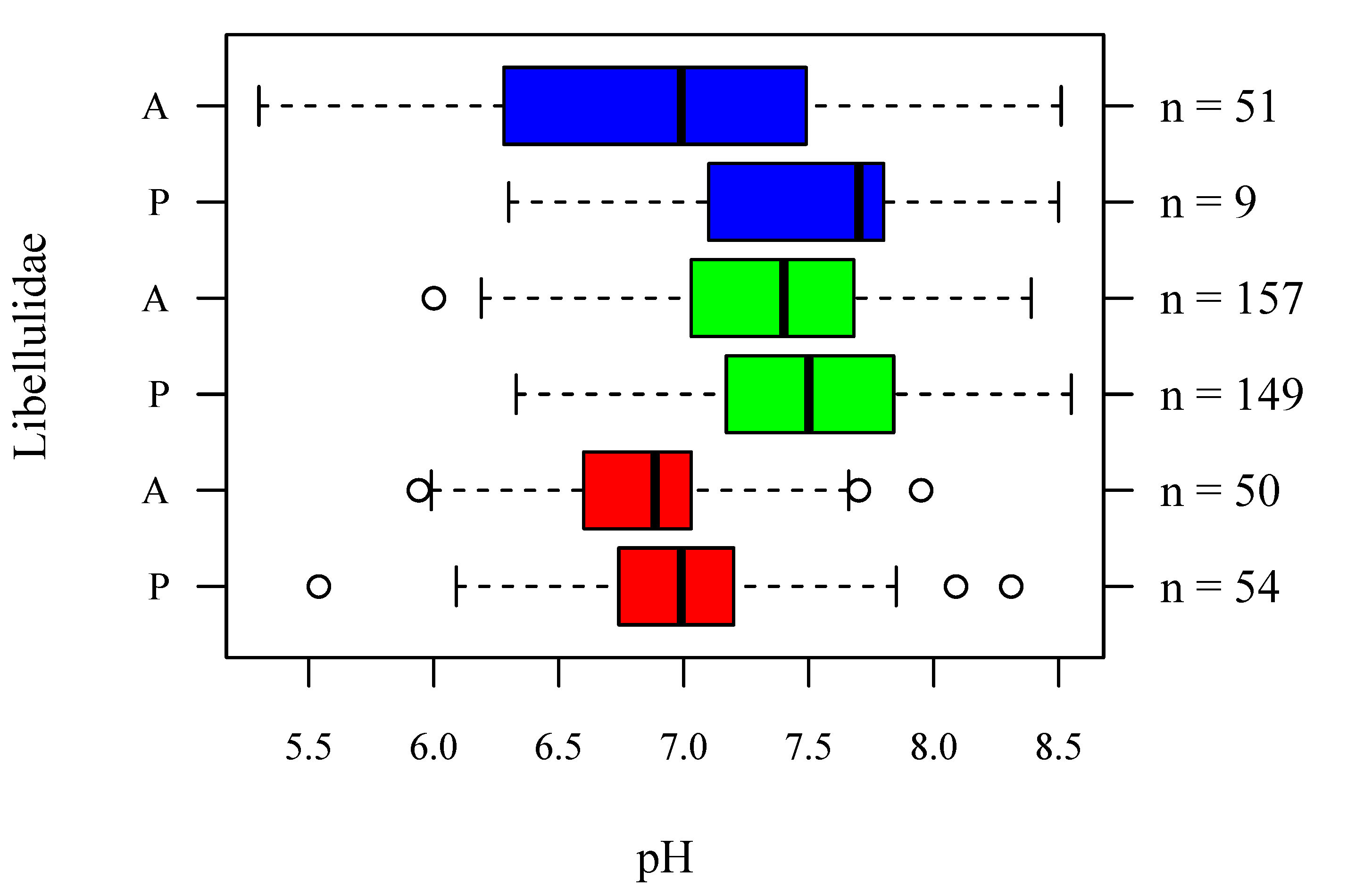

Supplement: Figure S27 — Boxplots indicate the observed pH in Ecuador (red), Ethiopia (green) and Vietnam (blue) at which Libellulidae are found to be present (denoted by P on the left axis) and absent (denoted by A on the left axis). The sample sizes per boxplot are shown on the right axis. (DOCX) [file pone.0108898.s027.docx]

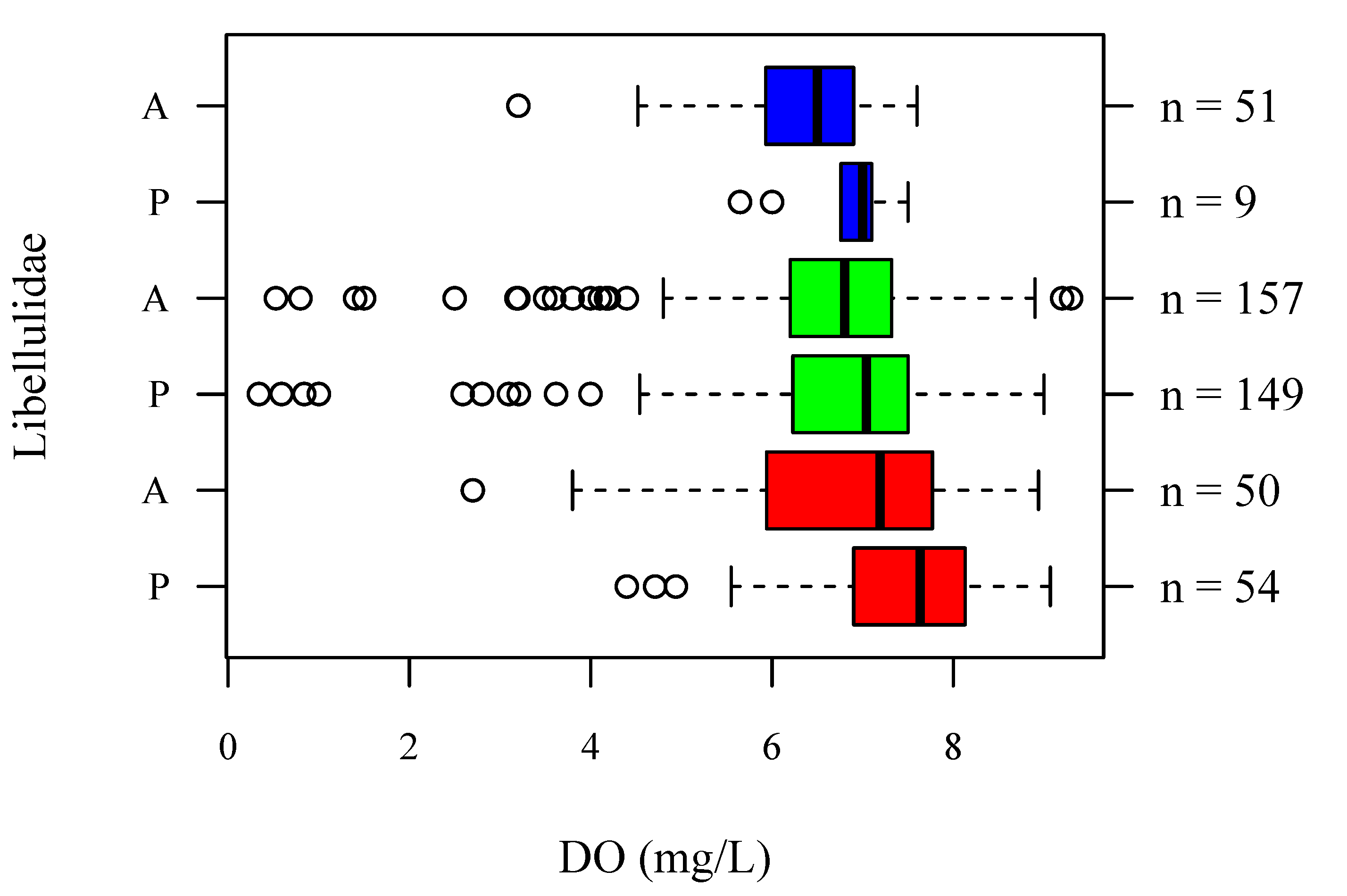

Supplement: Figure S28 — Boxplots indicate the observed DO concentrations in Ecuador (red), Ethiopia (green) and Vietnam (blue) at which Libellulidae are found to be present (denoted by P on the left axis) and absent (denoted by A on the left axis). The sample sizes per boxplot are shown on the right axis. (DOCX) [file pone.0108898.s028.docx]

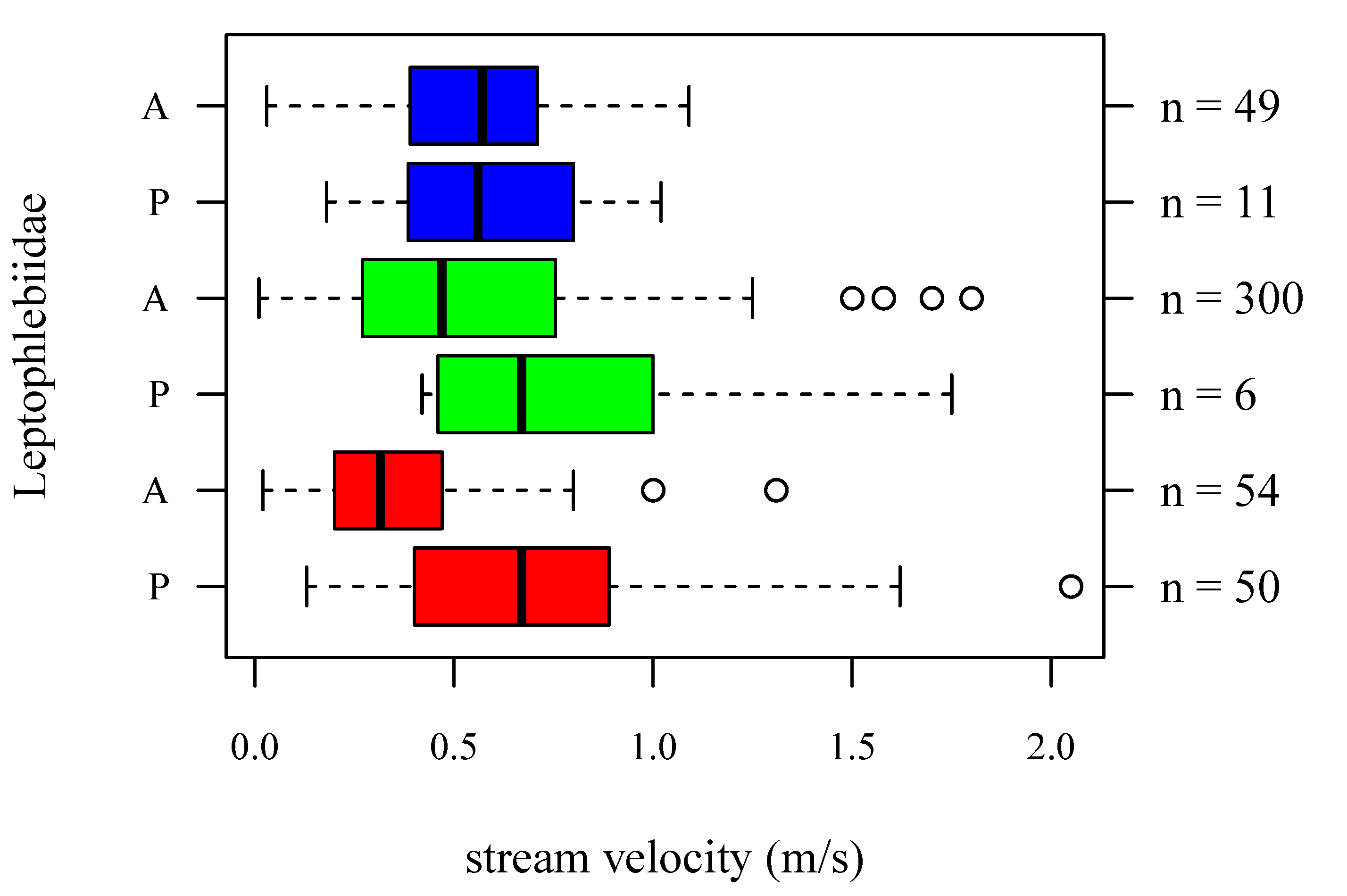

Supplement: Figure S29 — Boxplots indicate the observed stream velocity in Ecuador (red), Ethiopia (green) and Vietnam (blue) at which Leptophlebiidae are found to be present (denoted by P on the left axis) and absent (denoted by A on the left axis). The sample sizes per boxplot are shown on the right axis. (DOCX) [file pone.0108898.s029.docx]

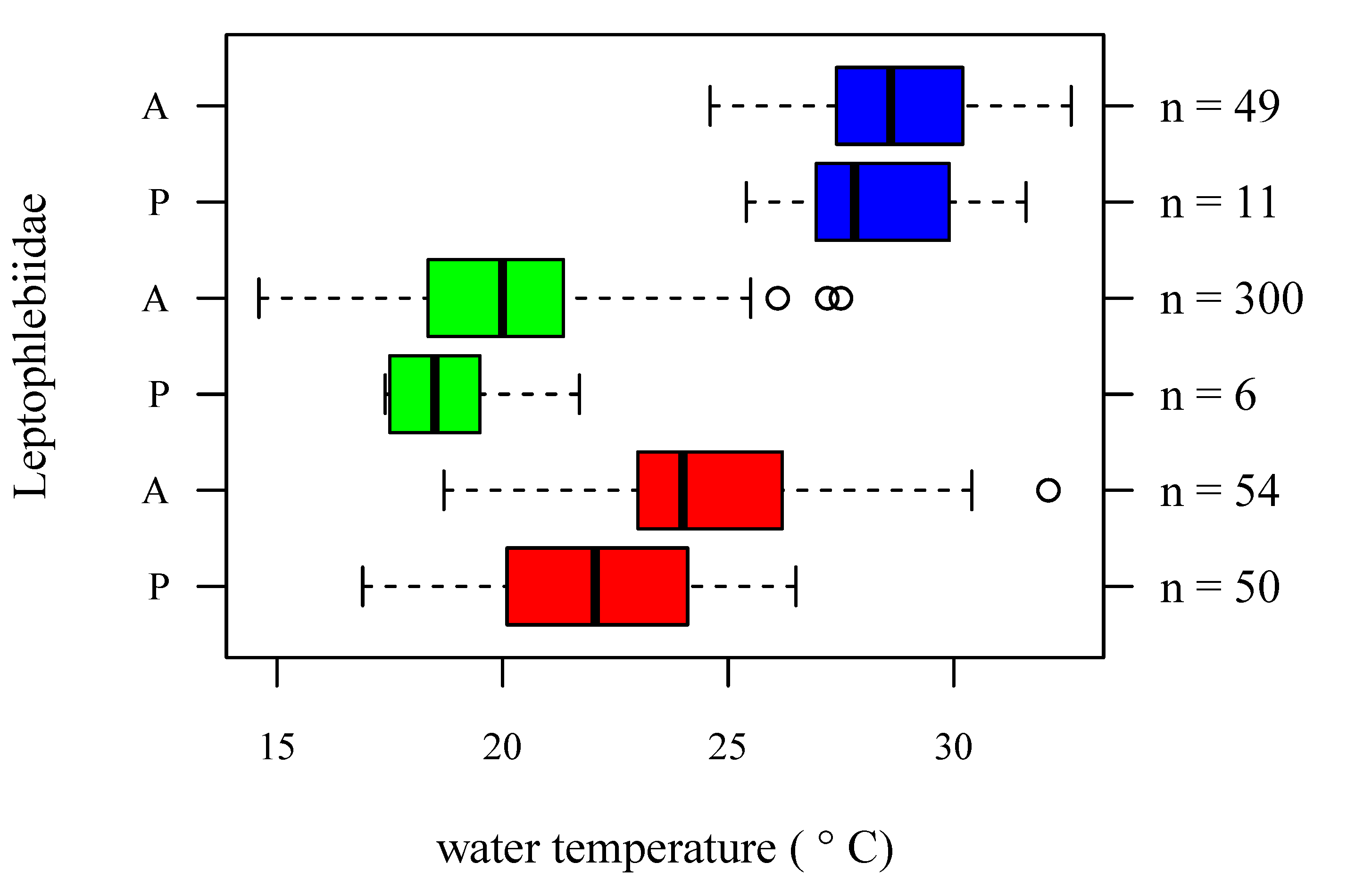

Supplement: Figure S30 — Boxplots indicate the observed water temperature in Ecuador (red), Ethiopia (green) and Vietnam (blue) at which Leptophlebiidae are found to be present (denoted by P on the left axis) and absent (denoted by A on the left axis). The sample sizes per boxplot are shown on the right axis. (DOCX) [file pone.0108898.s030.docx]

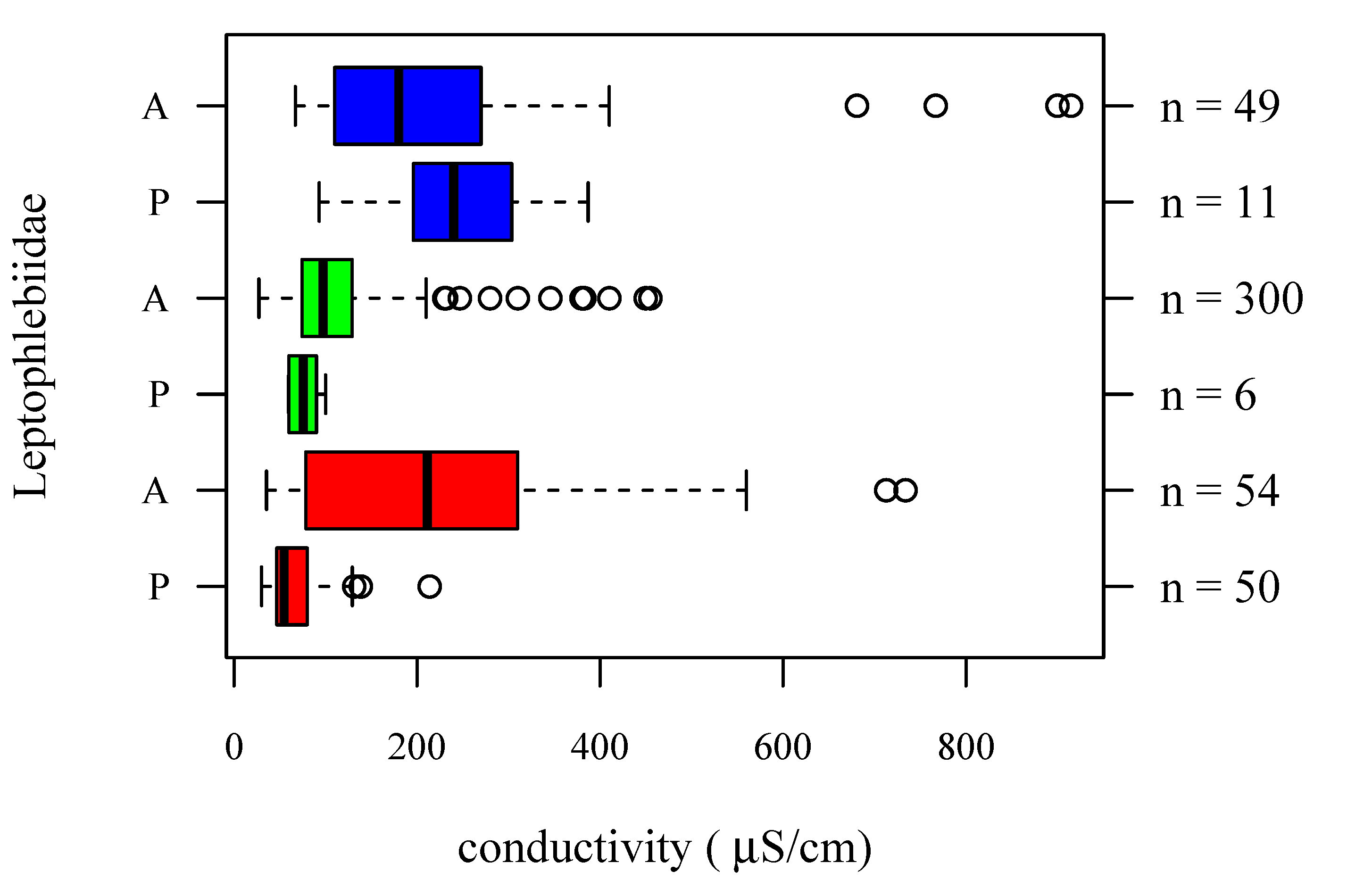

Supplement: Figure S31 — Boxplots indicate the observed conductivity in Ecuador (red), Ethiopia (green) and Vietnam (blue) at which Leptophlebiidae are found to be present (denoted by P on the left axis) and absent (denoted by A on the left axis). (DOCX) [file pone.0108898.s031.docx]

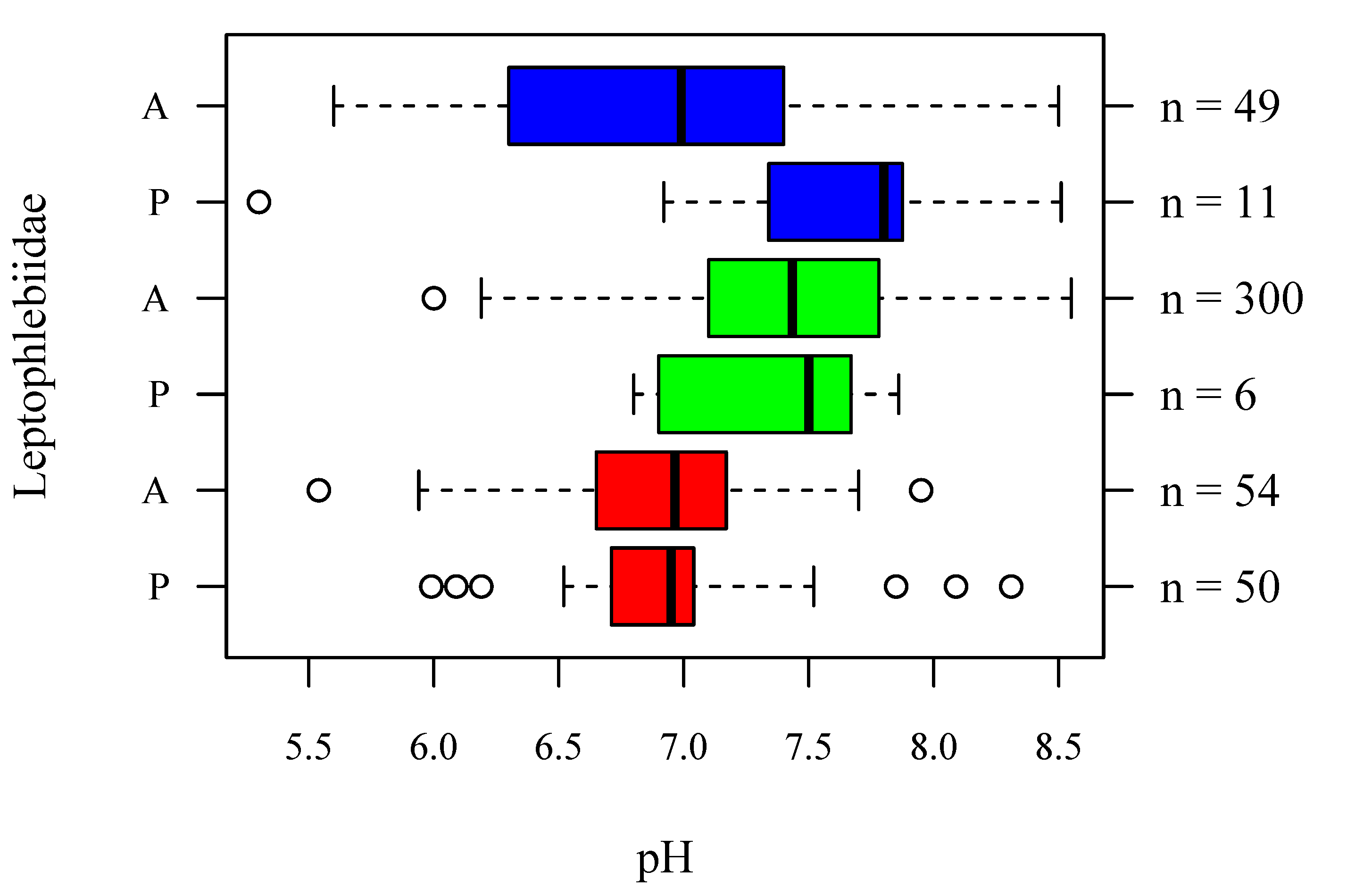

Supplement: Figure S32 — Boxplots indicate the observed pH in Ecuador (red), Ethiopia (green) and Vietnam (blue) at which Leptophlebiidae are found to be present (denoted by P on the left axis) and absent (denoted by A on the left axis). The sample sizes per boxplot are shown on the right axis. (DOCX) [file pone.0108898.s032.docx]

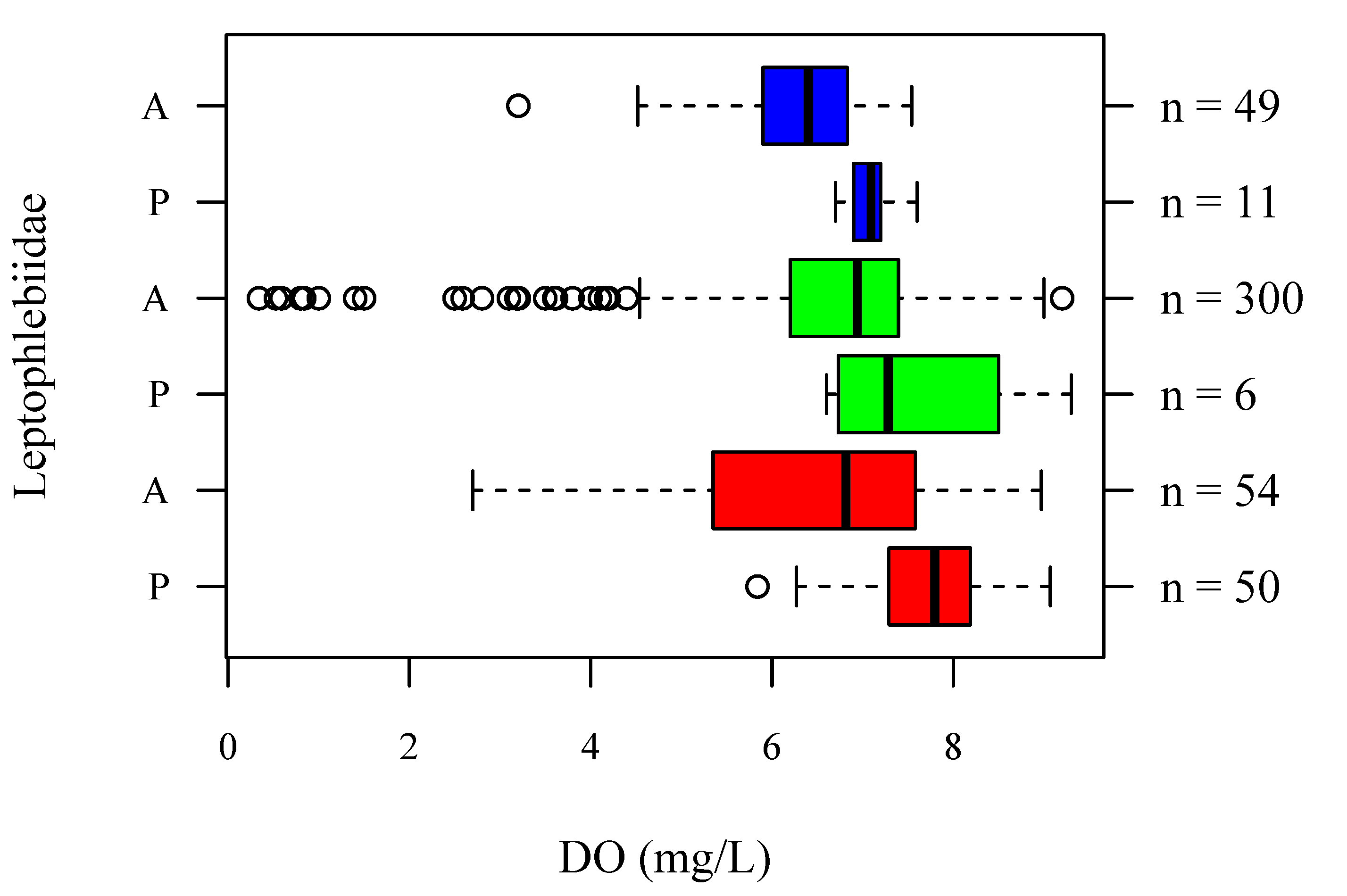

Supplement: Figure S33 — Boxplots indicate the observed DO concentrations in Ecuador (red), Ethiopia (green) and Vietnam (blue) at which Leptophlebiidae are found to be present (denoted by P on the left axis) and absent (denoted by A on the left axis). The sample sizes per boxplot are shown on the right axis. (DOCX) [file pone.0108898.s033.docx]

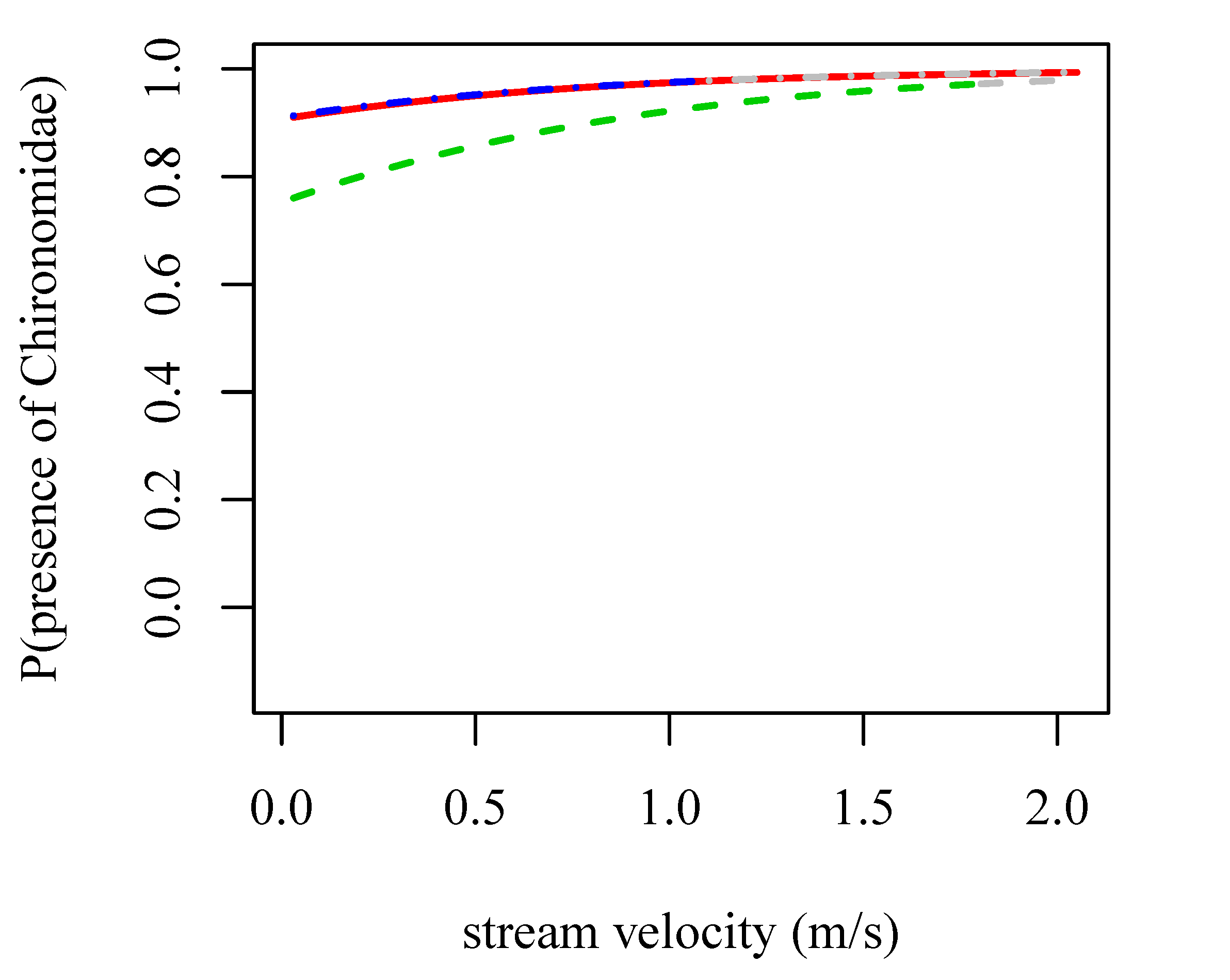

Supplement: Figure S34 — The probability of Chironomidae being present in relation to the stream velocity measured in Ecuador (red, solid), Ethiopia (green, dashed) and Vietnam (blue, dotdashed). The gray-colored ends of the response curves indicate extrapolation outside the observed physical-chemical range in the corresponding river basin. (DOCX) [file pone.0108898.s034.docx]

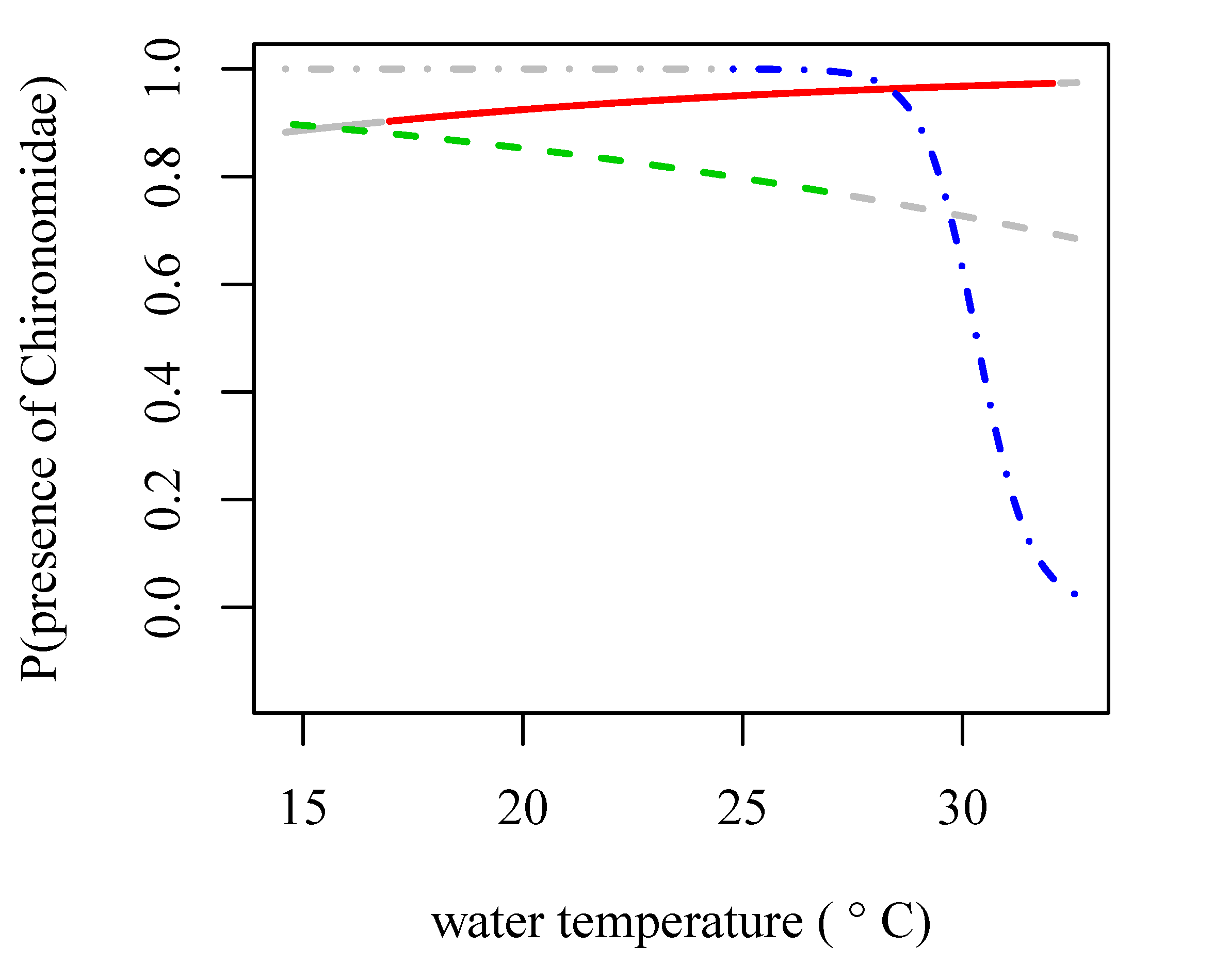

Supplement: Figure S35 — The probability of Chironomidae being present in relation to the water temperature measured in Ecuador (red, solid), Ethiopia (green, dashed) and Vietnam (blue, dotdashed). The gray-colored ends of the response curves indicate extrapolation outside the observed physical-chemical range in the corresponding river basin. (DOCX) [file pone.0108898.s035.docx]

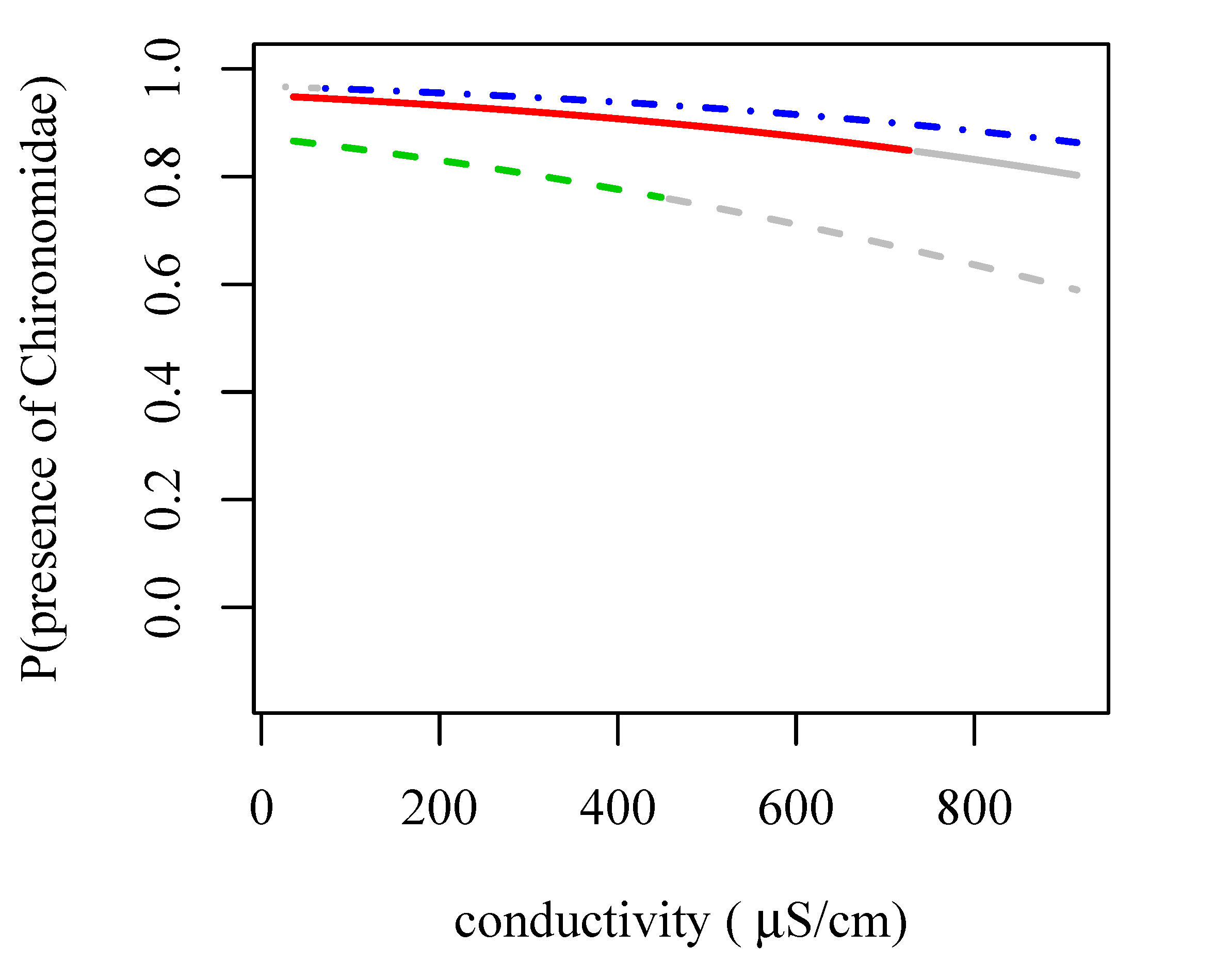

Supplement: Figure S36 — The probability of Chironomidae being present in relation to the conductivity measured in Ecuador (red, solid), Ethiopia (green, dashed) and Vietnam (blue, dotdashed). The gray-colored ends of the response curves indicate extrapolation outside the observed physical-chemical range in the corresponding river basin. (DOCX) [file pone.0108898.s036.docx]

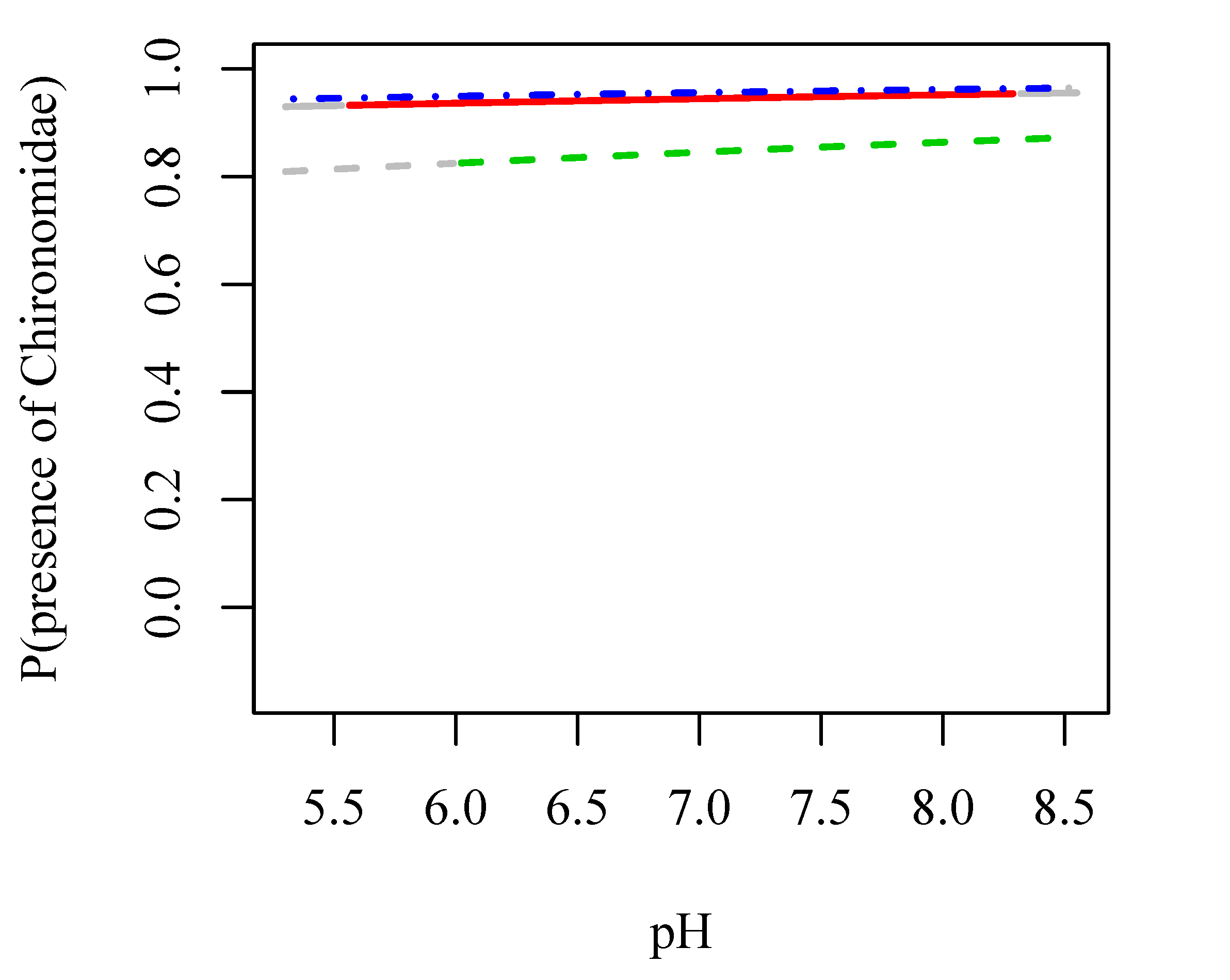

Supplement: Figure S37 — The probability of Chironomidae being present in relation to the pH measured in Ecuador (red, solid), Ethiopia (green, dashed) and Vietnam (blue, dotdashed). The gray-colored ends of the response curves indicate extrapolation outside the observed physical-chemical range in the corresponding river basin. (DOCX) [file pone.0108898.s037.docx]

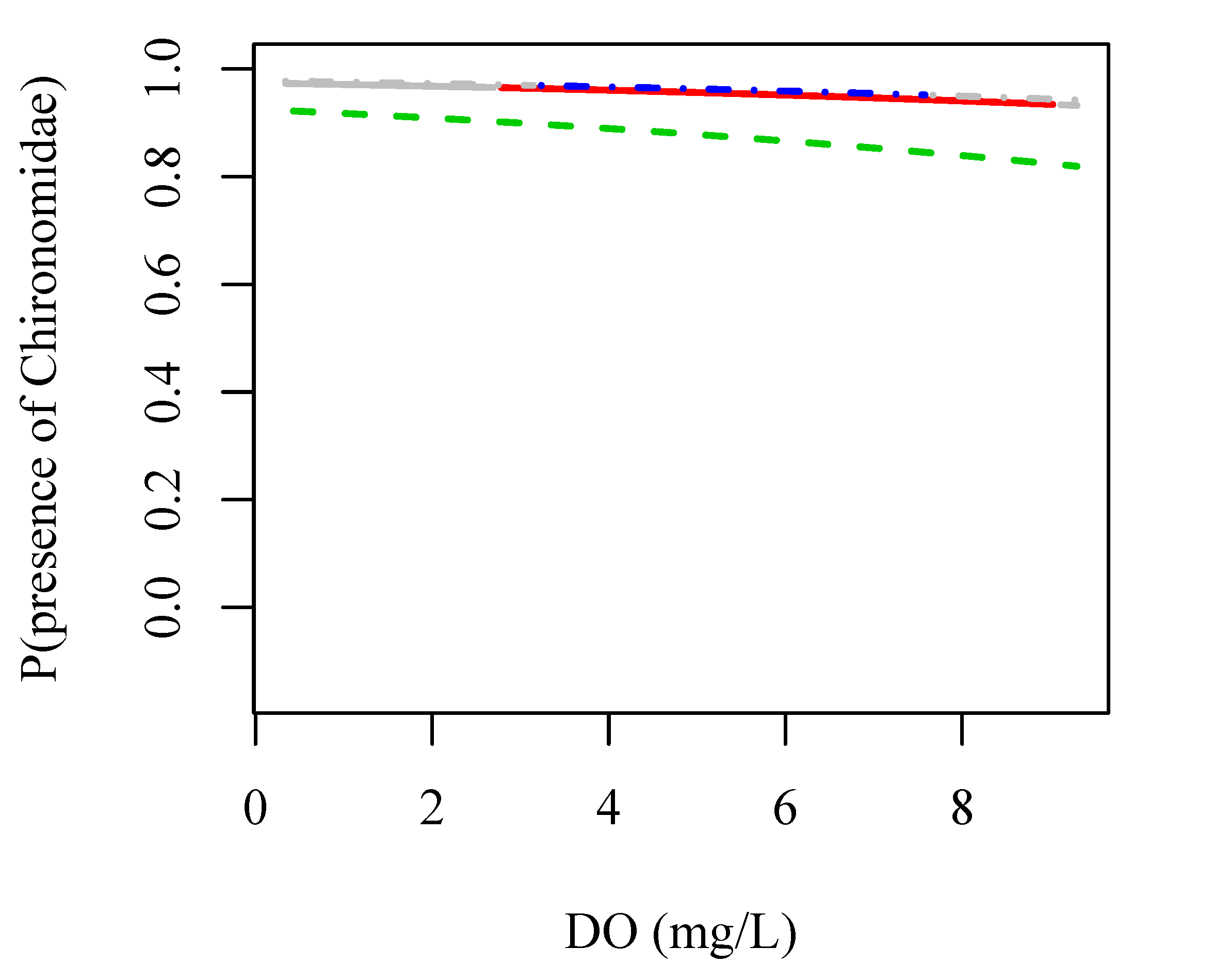

Supplement: Figure S38 — The probability of Chironomidae being present in relation to dissolved oxygen (DO) concentration measured in Ecuador (red, solid), Ethiopia (green, dashed) and Vietnam (blue, dotdashed). The gray-colored ends of the response curves indicate extrapolation outside the observed physical-chemical range in the corresponding river basin. (DOCX) [file pone.0108898.s038.docx]

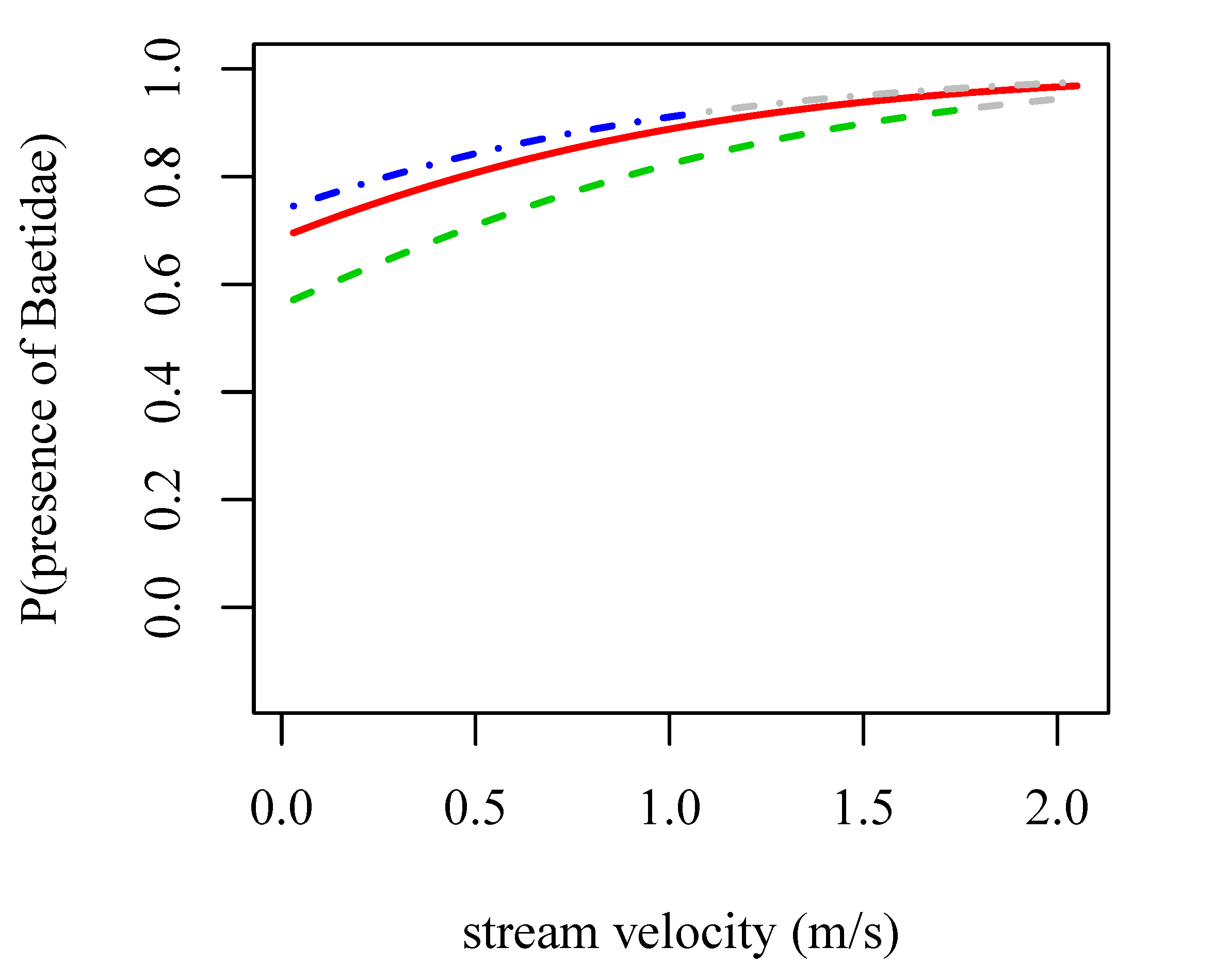

Supplement: Figure S39 — The probability of Baetidae being present in relation to stream velocity measured in Ecuador (red, solid), Ethiopia (green, dashed) and Vietnam (blue, dotdashed). The gray-colored ends of the response curves indicate extrapolation outside the observed physical-chemical range in the corresponding river basin. (DOCX) [file pone.0108898.s039.docx]

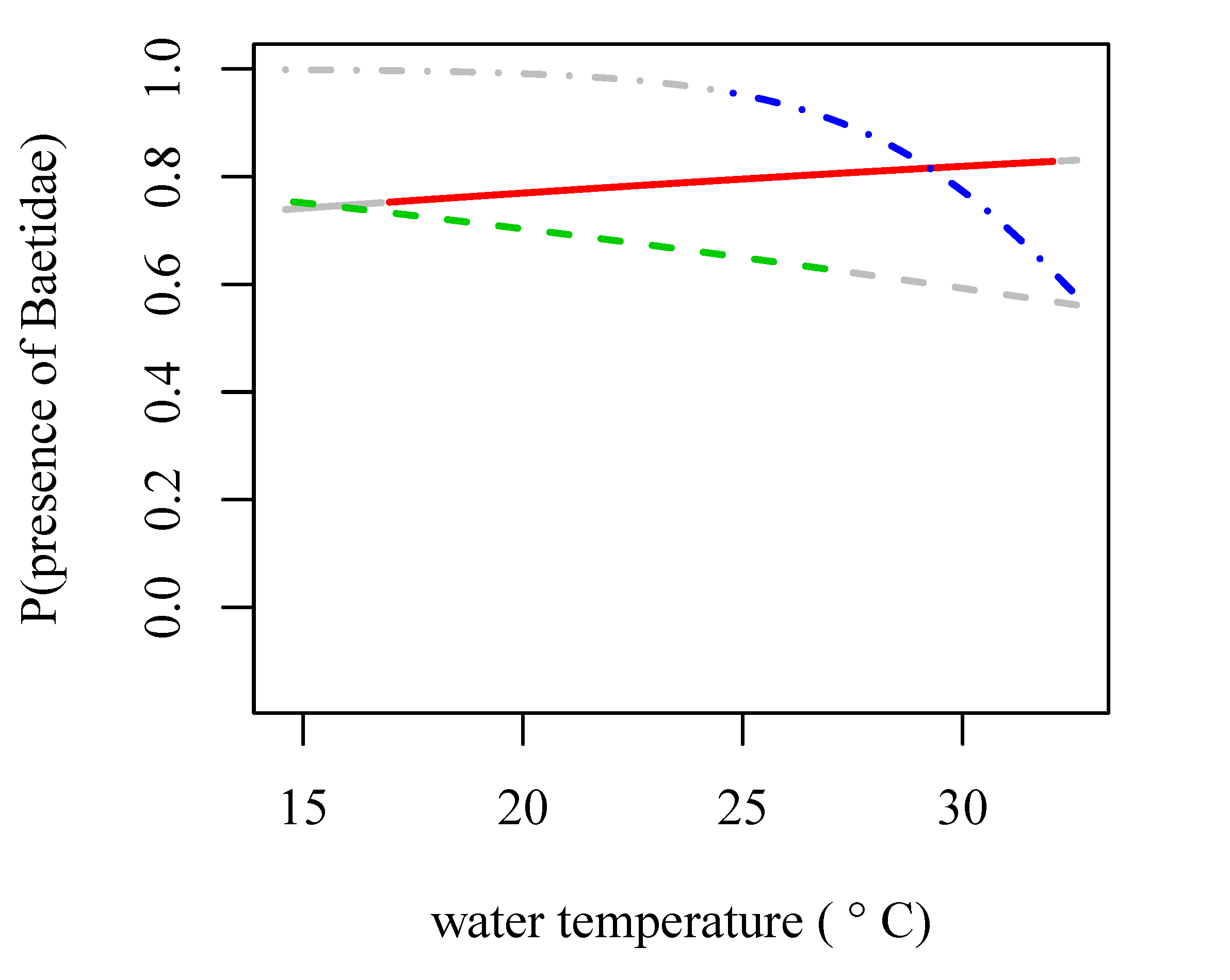

Supplement: Figure S40 — The probability of Baetidae being present in relation to water temperature measured in Ecuador (red, solid), Ethiopia (green, dashed) and Vietnam (blue, dotdashed). The gray-colored ends of the response curves indicate extrapolation outside the observed physical-chemical range in the corresponding river basin. (DOCX) [file pone.0108898.s040.docx]

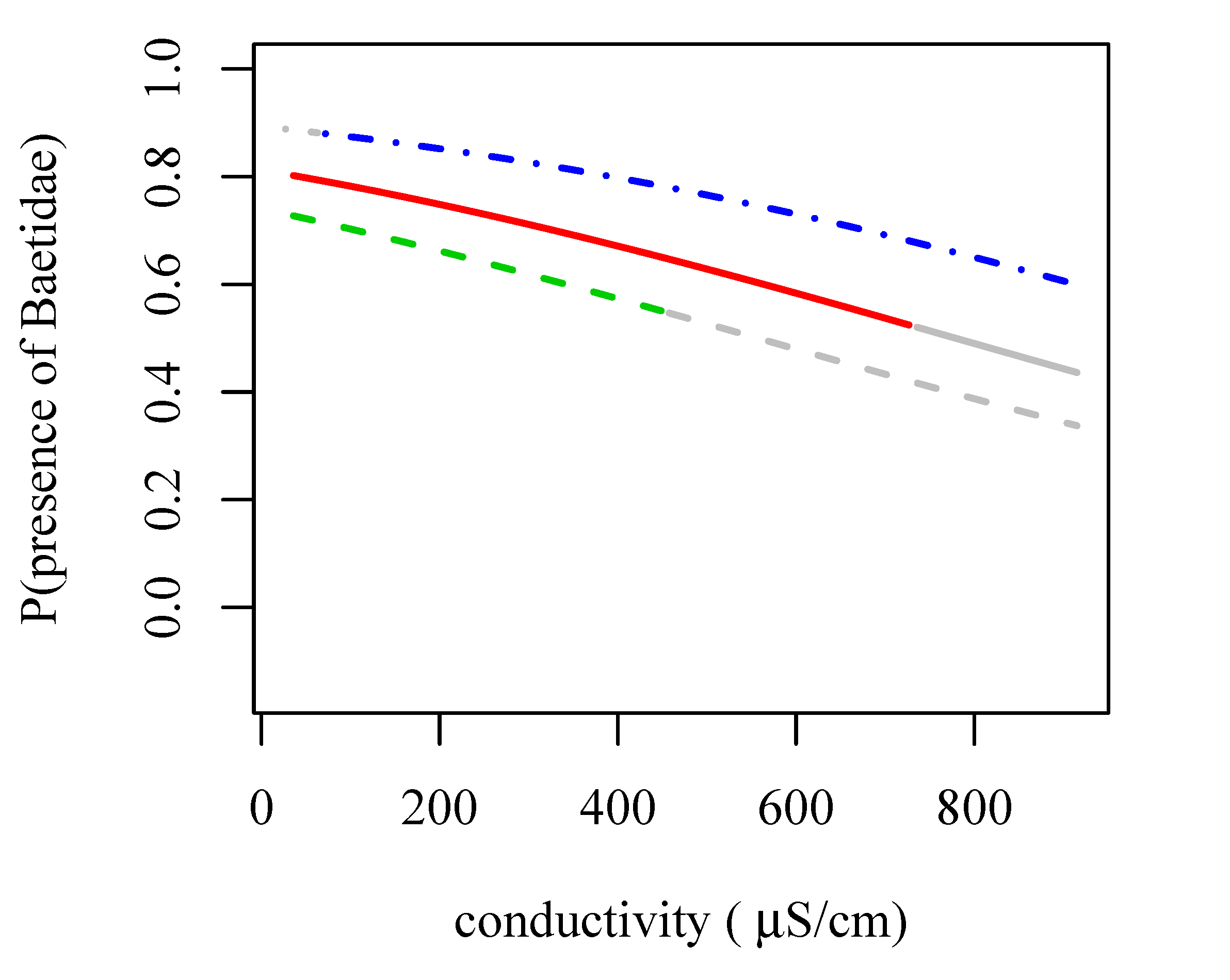

Supplement: Figure S41 — The probability of Baetidae being present in relation to conductivity measured in Ecuador (red, solid), Ethiopia (green, dashed) and Vietnam (blue, dotdashed). The gray-colored ends of the response curves indicate extrapolation outside the observed physical-chemical range in the corresponding river basin. (DOCX) [file pone.0108898.s041.docx]

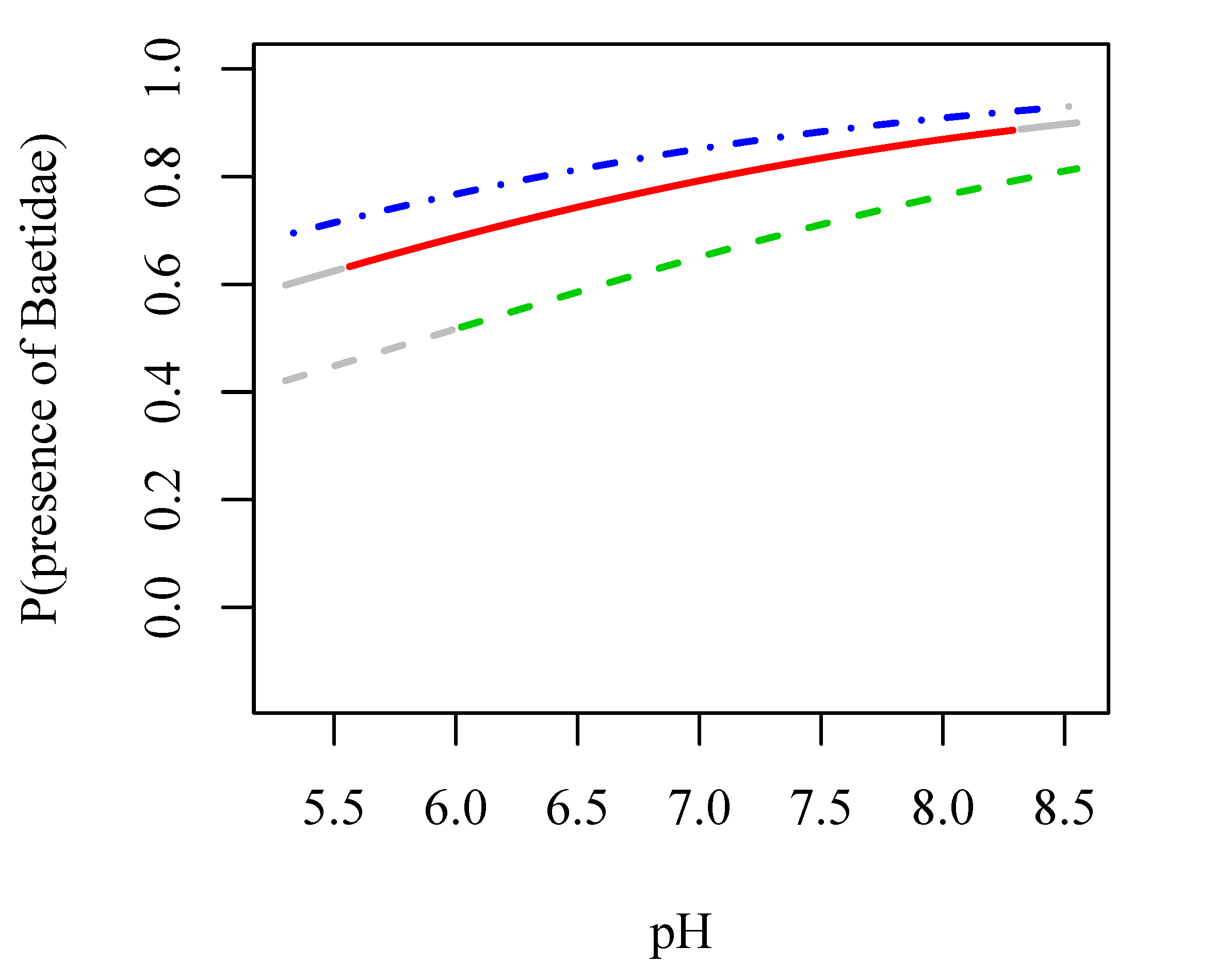

Supplement: Figure S42 — The probability of Baetidae being present in relation to pH measured in Ecuador (red, solid), Ethiopia (green, dashed) and Vietnam (blue, dotdashed). The gray-colored ends of the response curves indicate extrapolation outside the observed physical-chemical range in the corresponding river basin. (DOCX) [file pone.0108898.s042.docx]

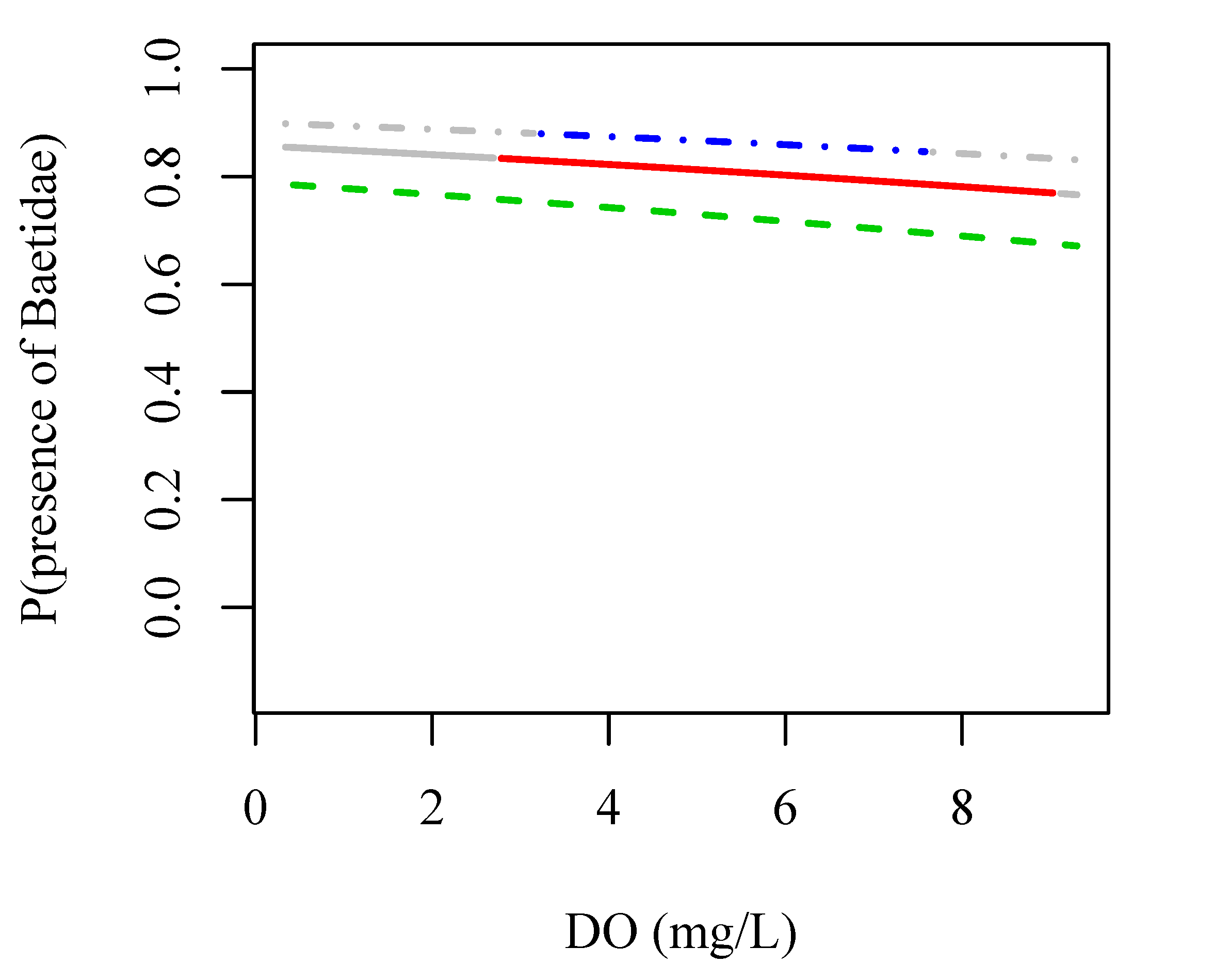

Supplement: Figure S43 — The probability of Baetidae being present in relation to dissolved oxygen (DO) concentration measured in Ecuador (red, solid), Ethiopia (green, dashed) and Vietnam (blue, dotdashed). The gray-colored ends of the response curves indicate extrapolation outside the observed physical-chemical range in the corresponding river basin. (DOCX) [file pone.0108898.s043.docx]

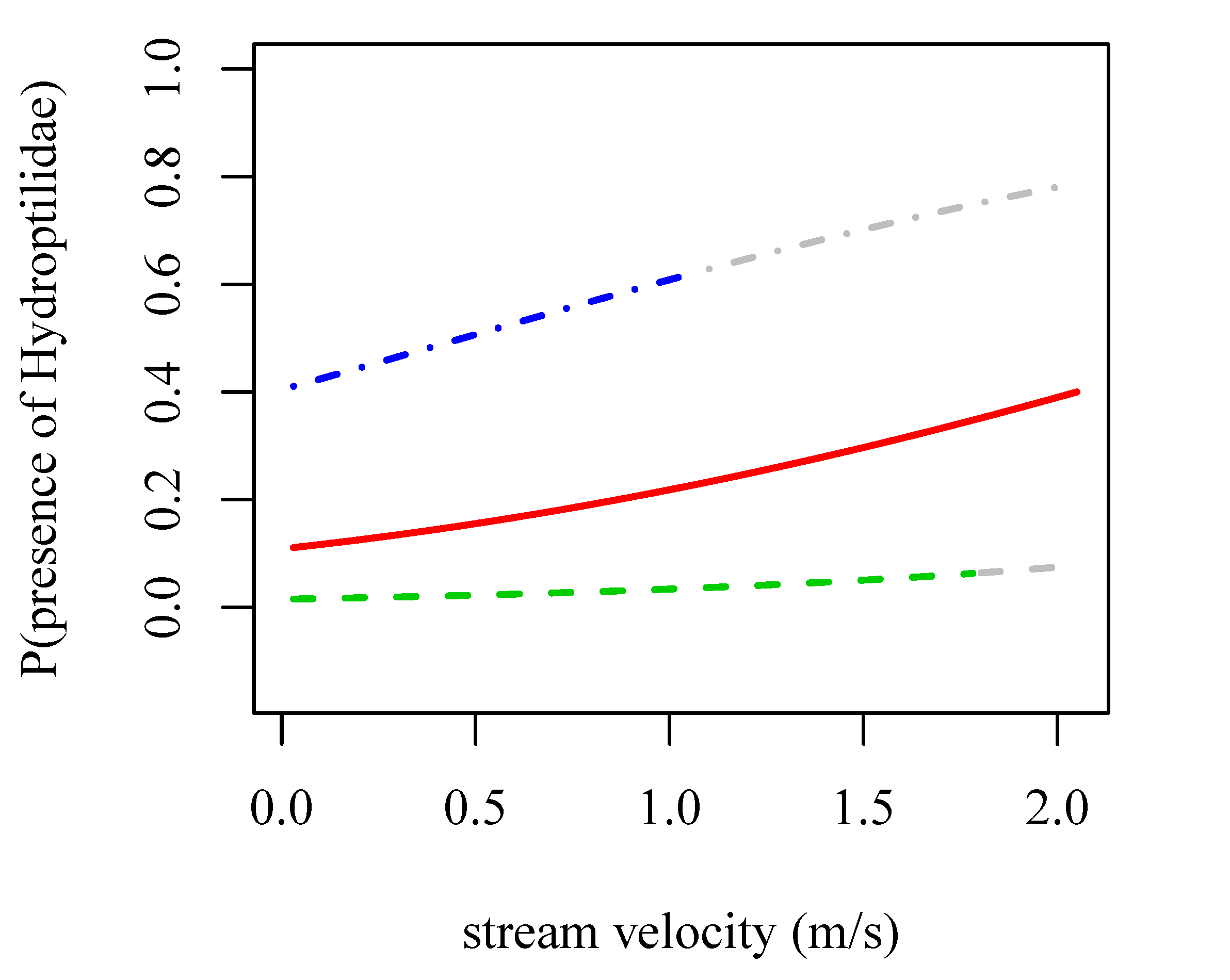

Supplement: Figure S44 — The probability of Hydroptilidae being present in relation to stream velocity measured in Ecuador (red, solid), Ethiopia (green, dashed) and Vietnam (blue, dotdashed). The gray-colored ends of the response curves indicate extrapolation outside the observed physical-chemical range in the corresponding river basin. (DOCX) [file pone.0108898.s044.docx]

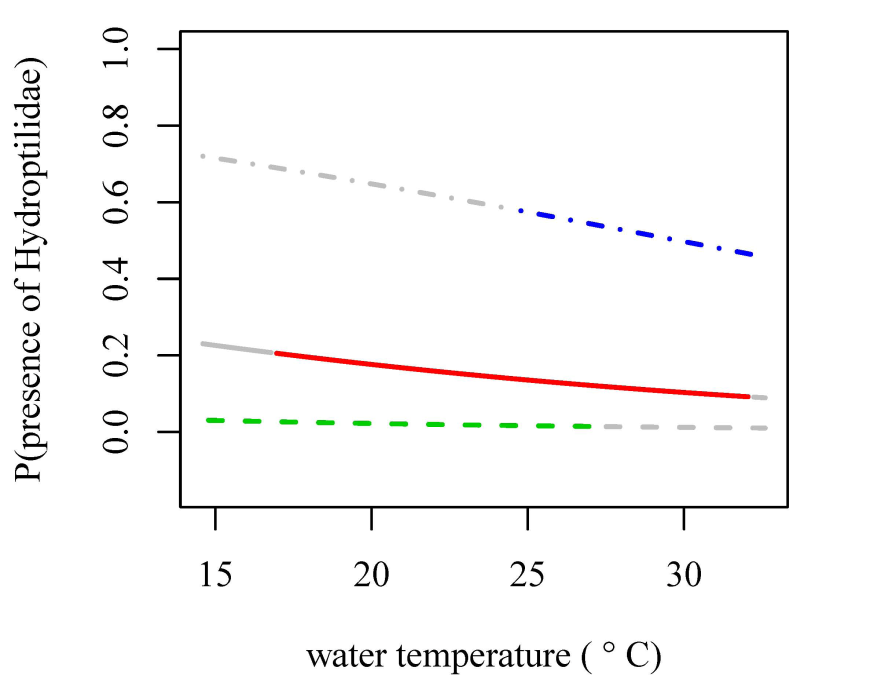

Supplement: Figure S45 — The probability of Chironomidae being present in relation to the Hydroptilidae measured in Ecuador (red, solid), Ethiopia (green, dashed) and Vietnam (blue, dotdashed). The gray-colored ends of the response curves indicate extrapolation outside the observed physical-chemical range in the corresponding river basin. (DOCX) [file pone.0108898.s045.docx]

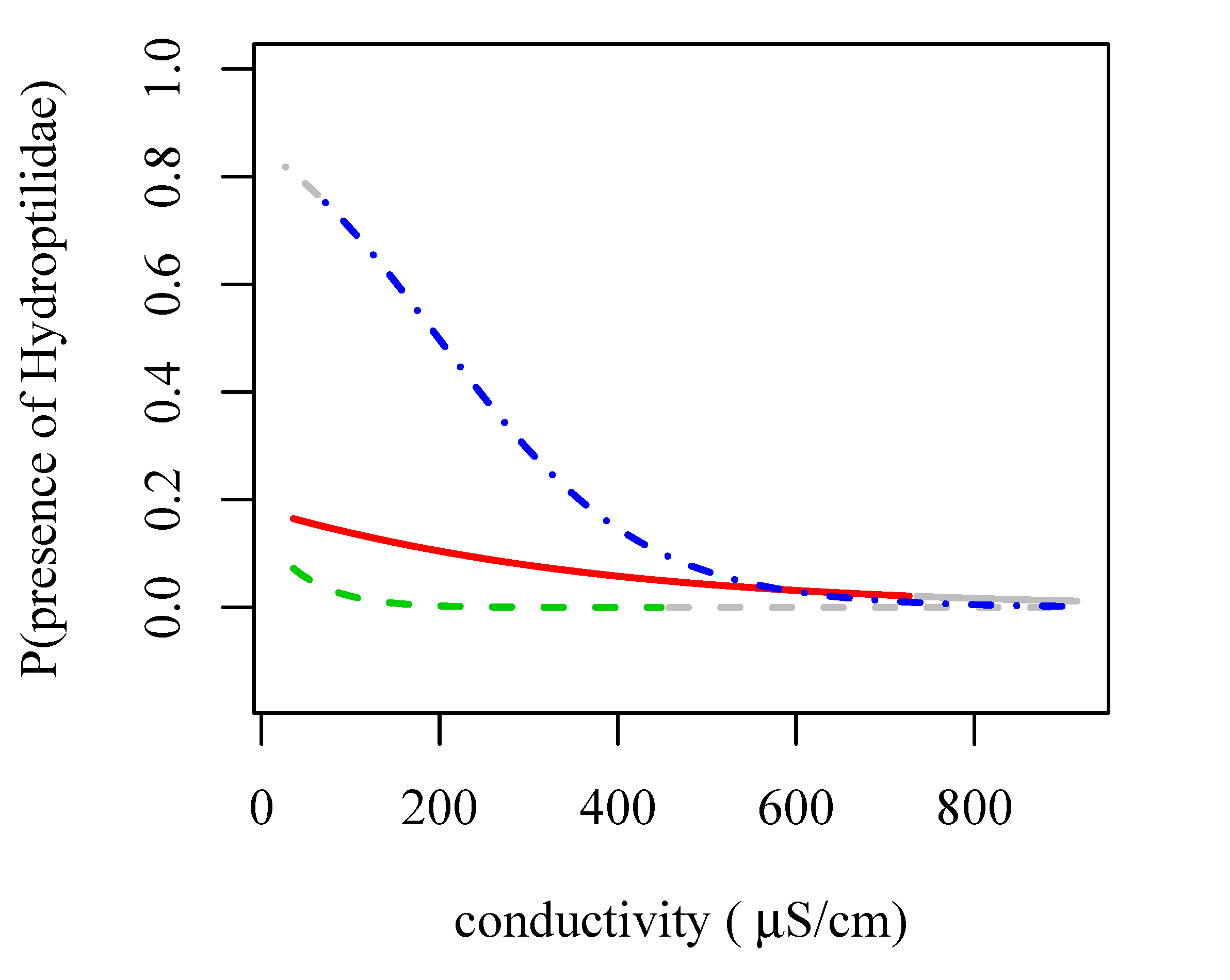

Supplement: Figure S46 — The probability of Hydroptilidae being present in relation to the conductivity measured in Ecuador (red, solid), Ethiopia (green, dashed) and Vietnam (blue, dotdashed). The gray-colored ends of the response curves indicate extrapolation outside the observed physical-chemical range in the corresponding river basin. (DOCX) [file pone.0108898.s046.docx]

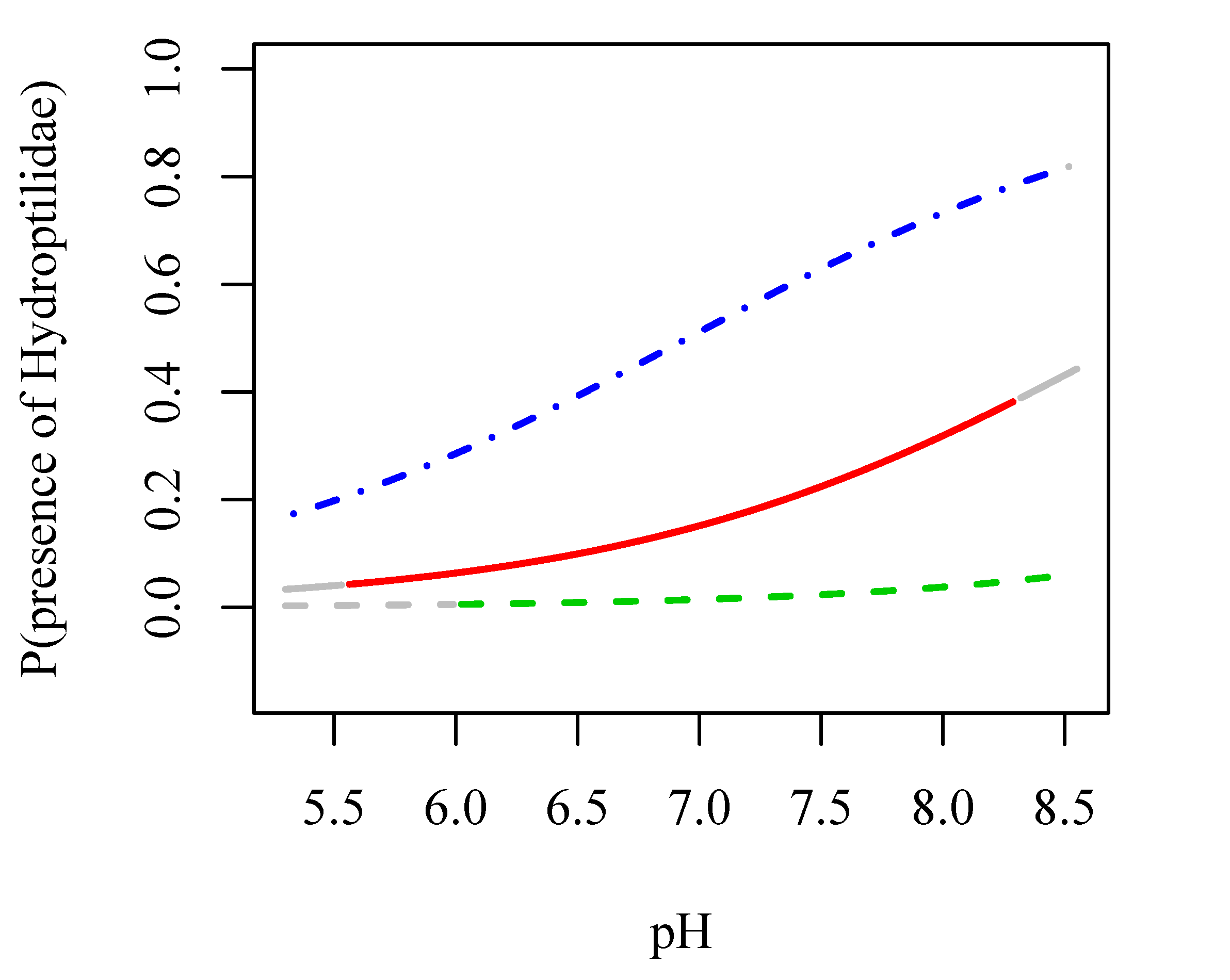

Supplement: Figure S47 — The probability of Hydroptilidae being present in relation to pH measured in Ecuador (red, solid), Ethiopia (green, dashed) and Vietnam (blue, dotdashed). The gray-colored ends of the response curves indicate extrapolation outside the observed physical-chemical range in the corresponding river basin. (DOCX) [file pone.0108898.s047.docx]

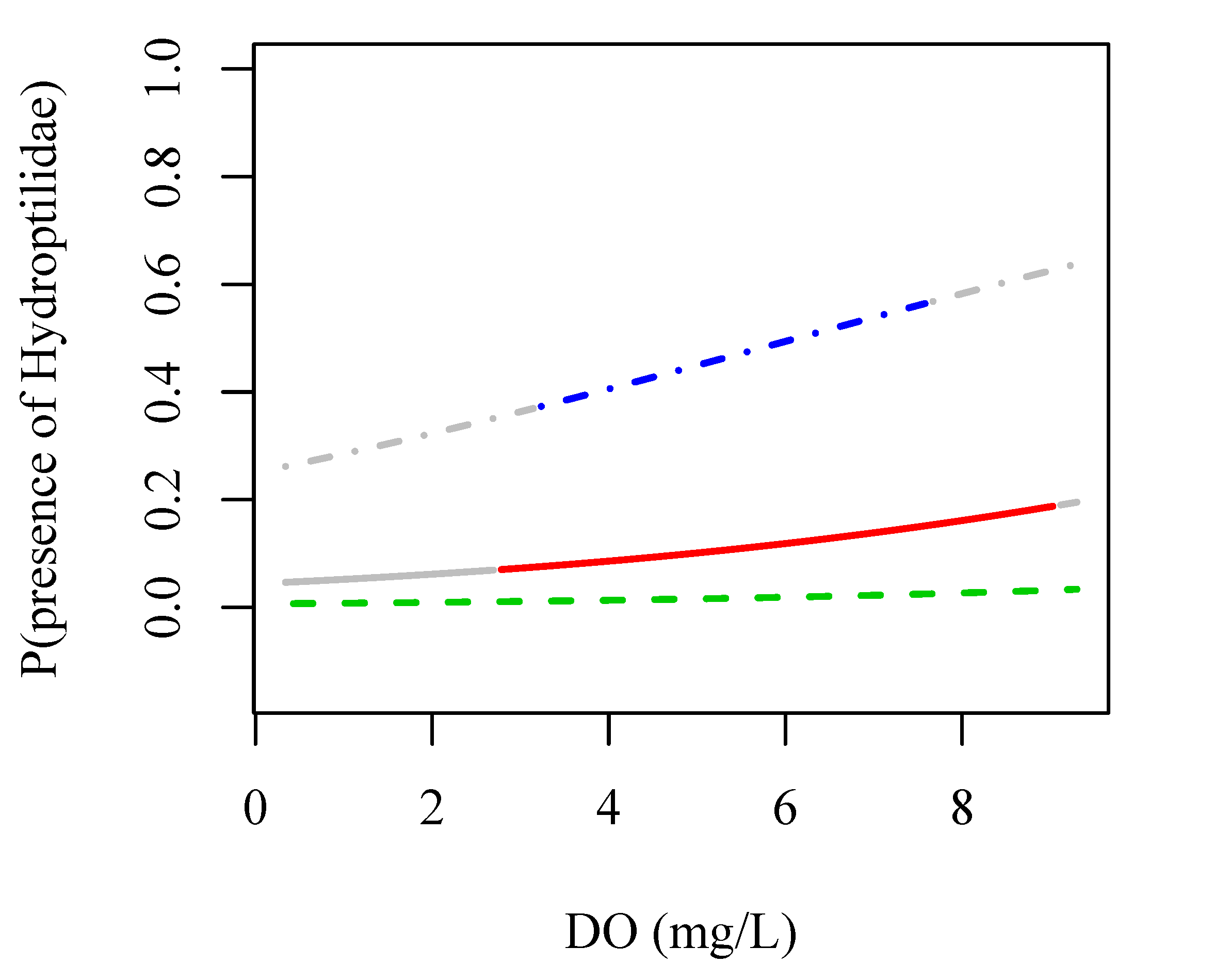

Supplement: Figure S48 — The probability of Hydroptilidae being present in relation to dissolved oxygen (DO) concentration measured in Ecuador (red, solid), Ethiopia (green, dashed) and Vietnam (blue, dotdashed). The gray-colored ends of the response curves indicate extrapolation outside the observed physical-chemical range in the corresponding river basin. (DOCX) [file pone.0108898.s048.docx]

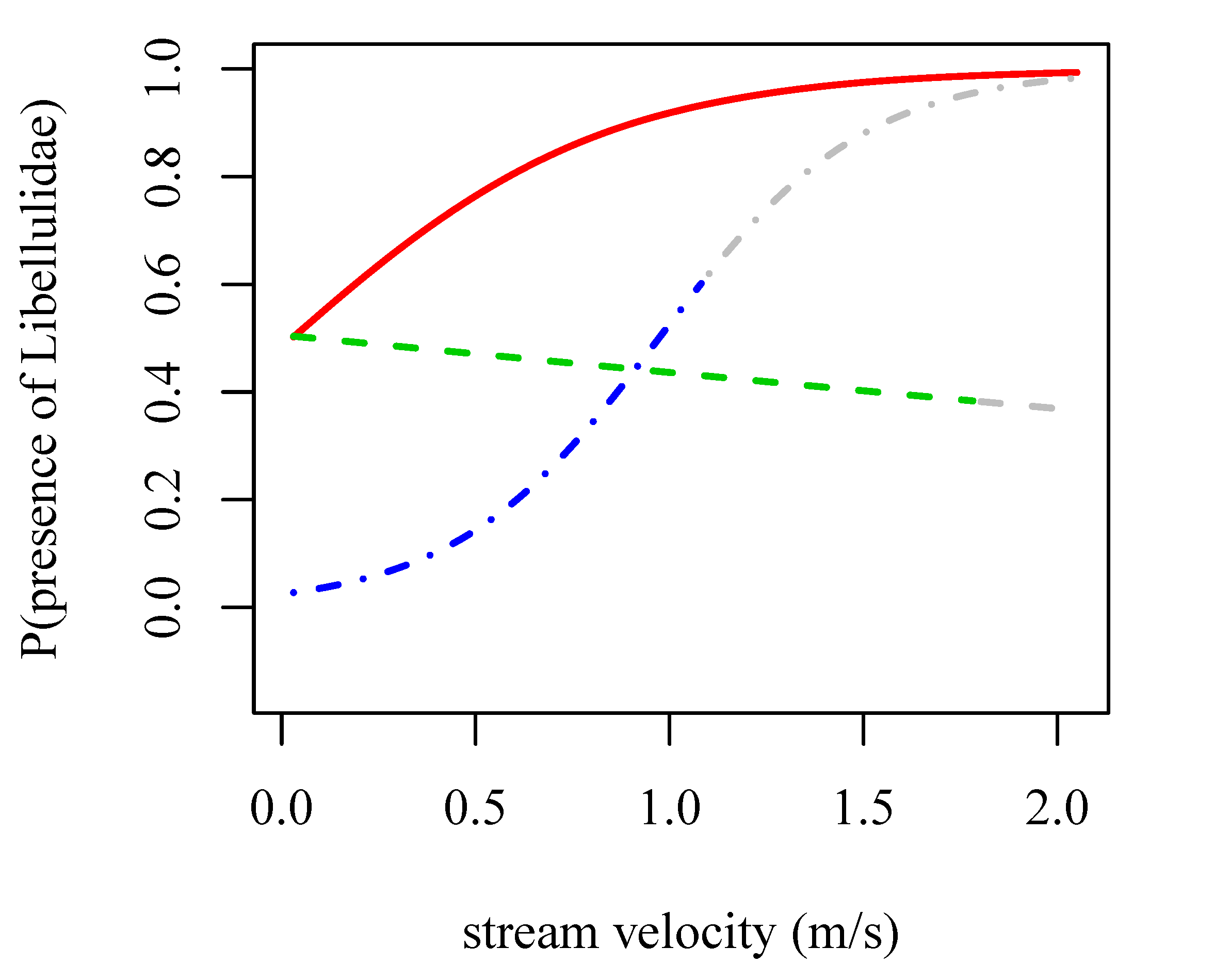

Supplement: Figure S49 — The probability of Libellulidae being present in relation to stream velocity measured in Ecuador (red, solid), Ethiopia (green, dashed) and Vietnam (blue, dotdashed). The gray-colored ends of the response curves indicate extrapolation outside the observed physical-chemical range in the corresponding river basin. (DOCX) [file pone.0108898.s049.docx]

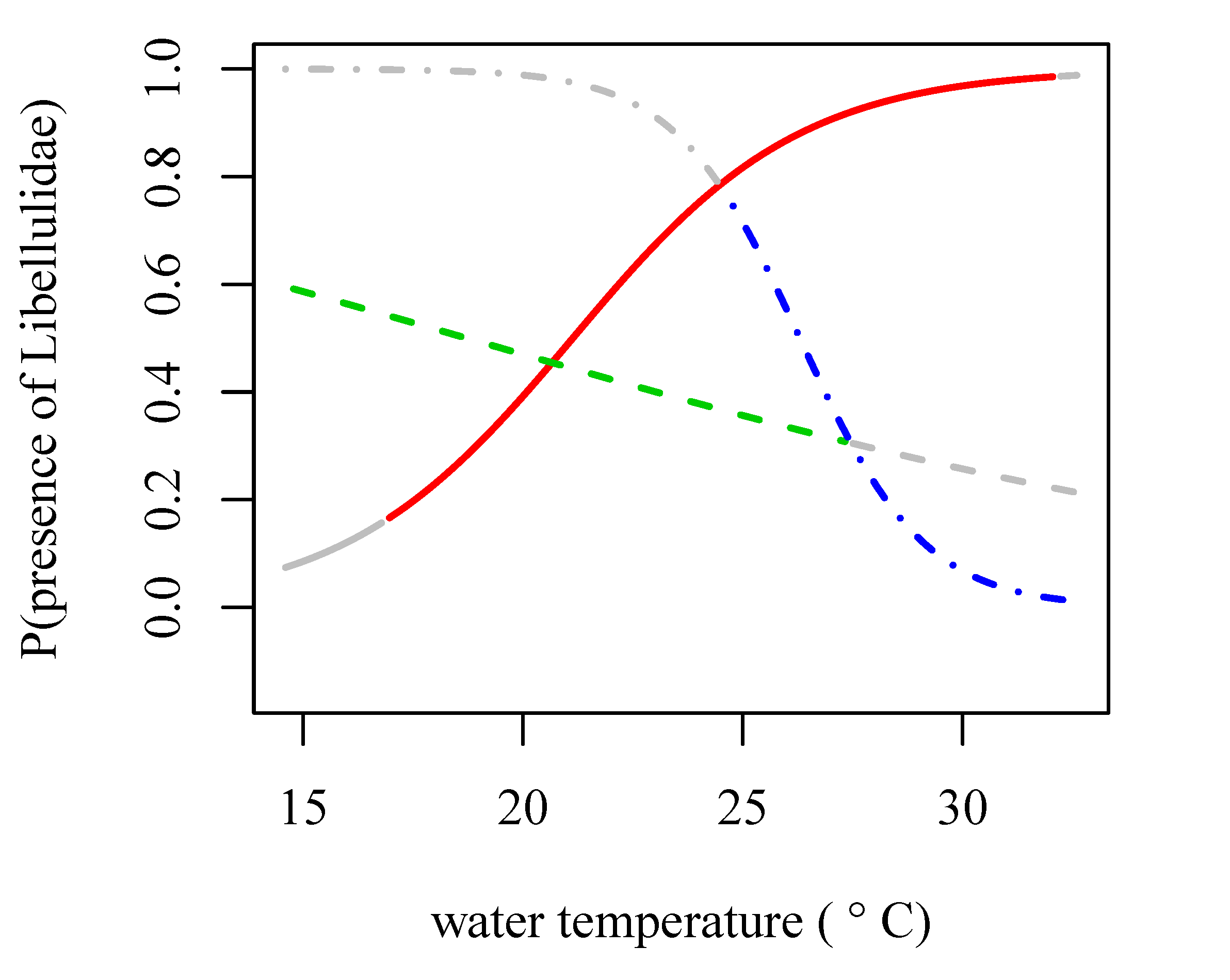

Supplement: Figure S50 — The probability of Libellulidae being present in relation to the water temperature measured in Ecuador (red, solid), Ethiopia (green, dashed) and Vietnam (blue, dotdashed). The gray-colored ends of the response curves indicate extrapolation outside the observed physical-chemical range in the corresponding river basin. (DOCX) [file pone.0108898.s050.docx]

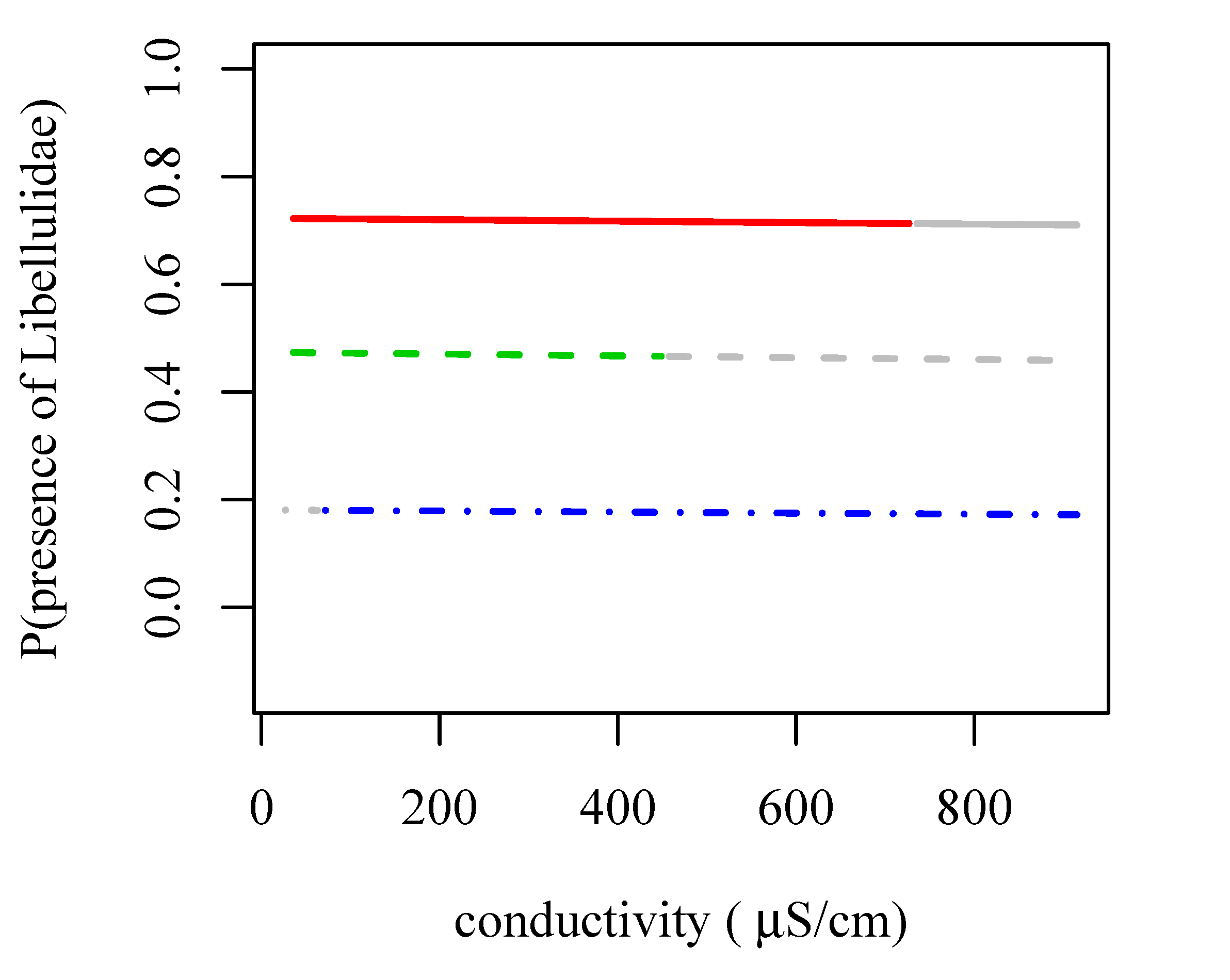

Supplement: Figure S51 — The probability of Libellulidae being present in relation to the conductivity measured in Ecuador (red, solid), Ethiopia (green, dashed) and Vietnam (blue, dotdashed). The gray-colored ends of the response curves indicate extrapolation outside the observed physical-chemical range in the corresponding river basin. (DOCX) [file pone.0108898.s051.docx]

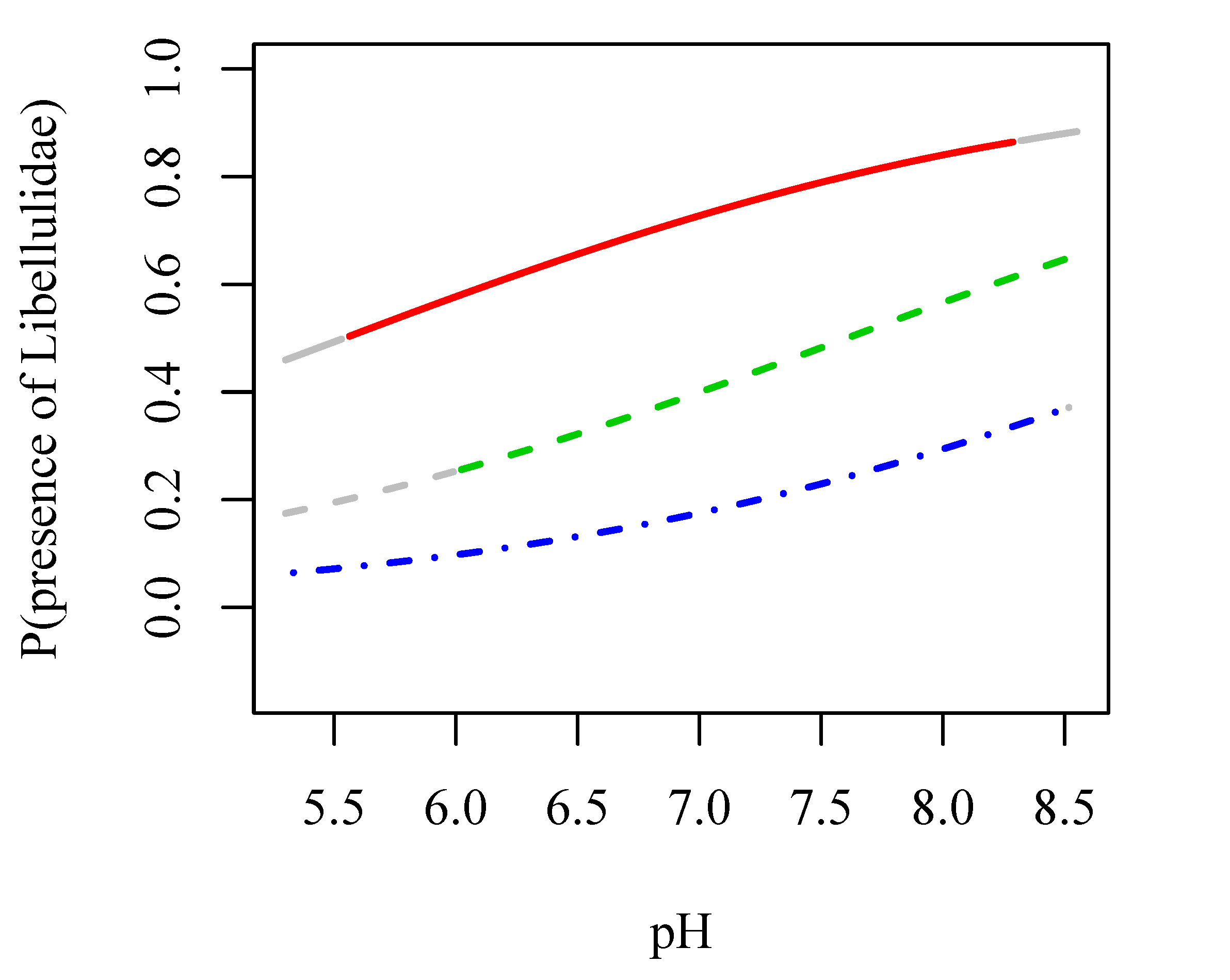

Supplement: Figure S52 — The probability of Libellulidae being present in relation to pH measured in Ecuador (red, solid), Ethiopia (green, dashed) and Vietnam (blue, dotdashed). The gray-colored ends of the response curves indicate extrapolation outside the observed physical-chemical range in the corresponding river basin. (DOCX) [file pone.0108898.s052.docx]

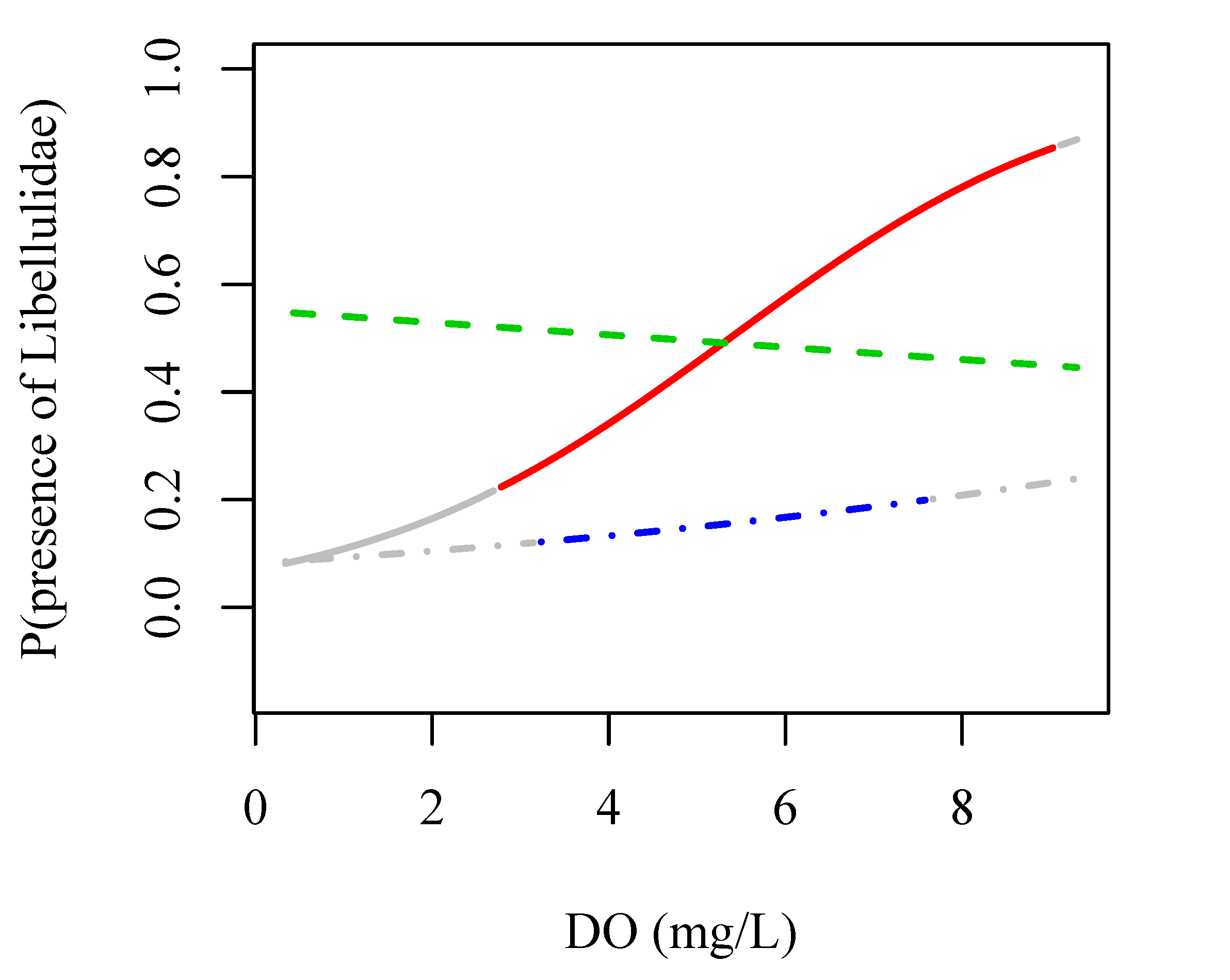

Supplement: Figure S53 — The probability of Libellulidae being present in relation to dissolved oxygen (DO) concentration measured in Ecuador (red, solid), Ethiopia (green, dashed) and Vietnam (blue, dotdashed). The gray-colored ends of the response curves indicate extrapolation outside the observed physical-chemical range in the corresponding river basin. (DOCX) [file pone.0108898.s053.docx]

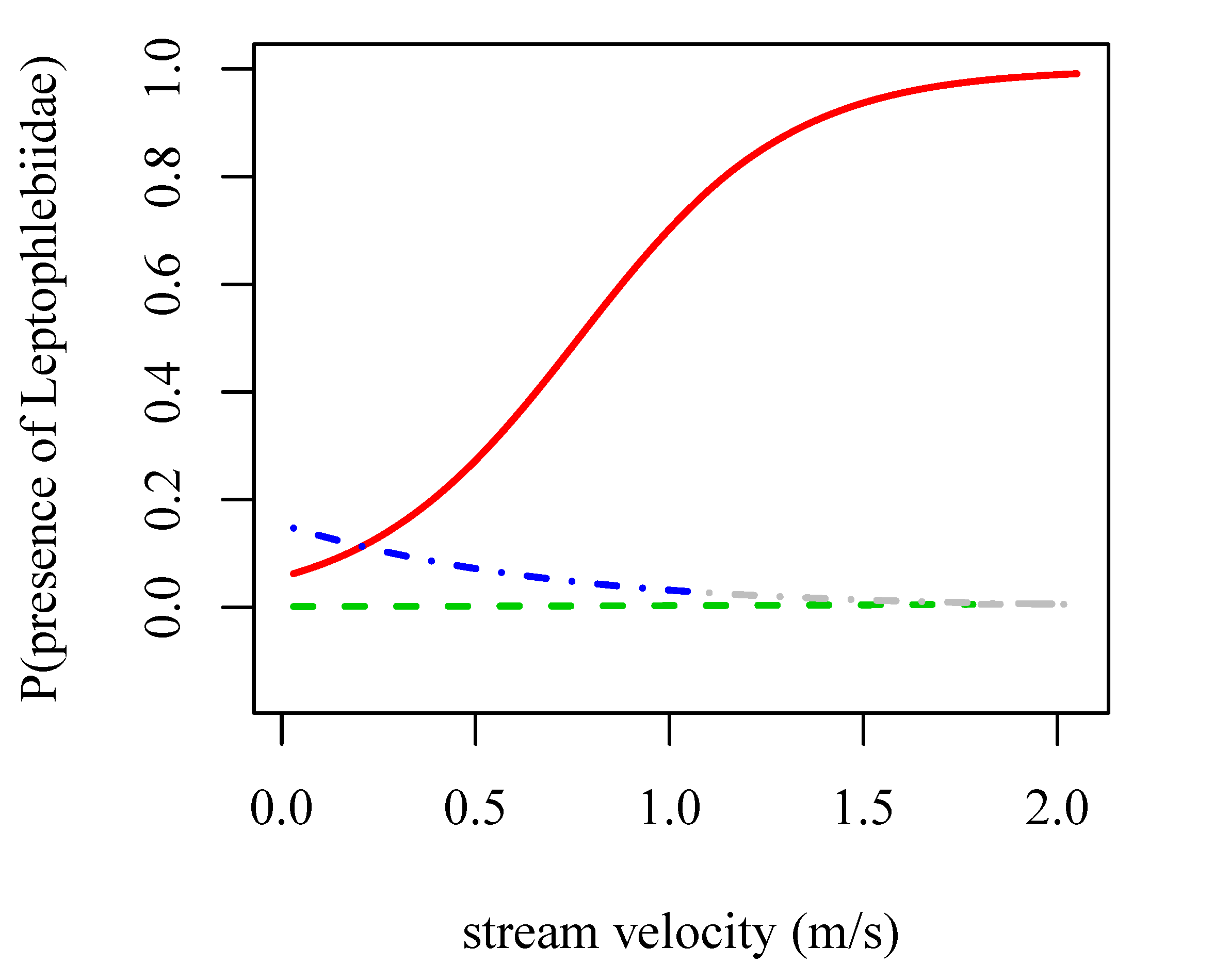

Supplement: Figure S54 — The probability of Leptophlebiidae being present in relation to stream velocity measured in Ecuador (red, solid), Ethiopia (green, dashed) and Vietnam (blue, dotdashed). The gray-colored ends of the response curves indicate extrapolation outside the observed physical-chemical range in the corresponding river basin. (DOCX) [file pone.0108898.s054.docx]

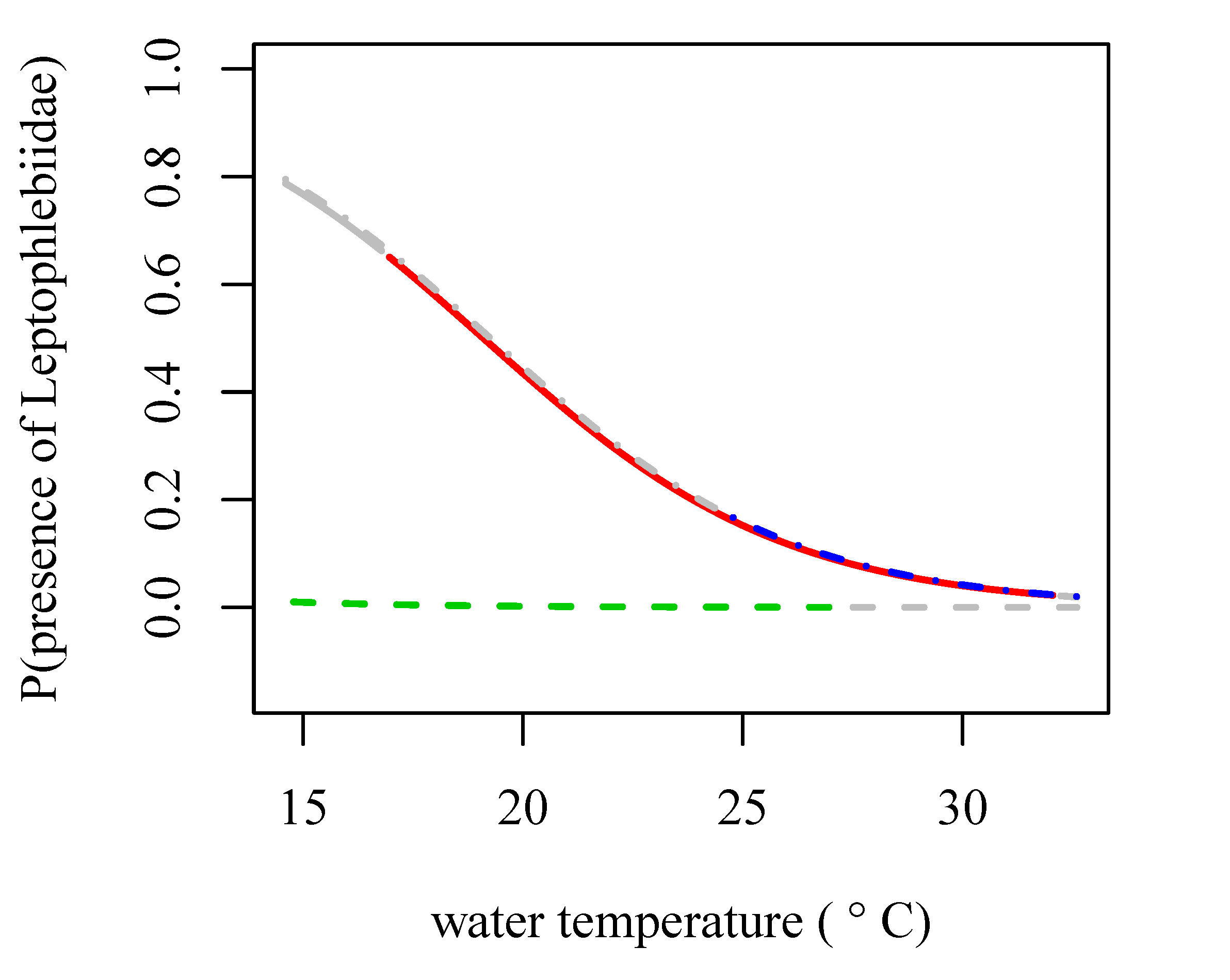

Supplement: Figure S55 — The probability of Leptophlebiidae being present in relation to the water temperature measured in Ecuador (red, solid), Ethiopia (green, dashed) and Vietnam (blue, dotdashed). The gray-colored ends of the response curves indicate extrapolation outside the observed physical-chemical range in the corresponding river basin. (DOCX) [file pone.0108898.s055.docx]

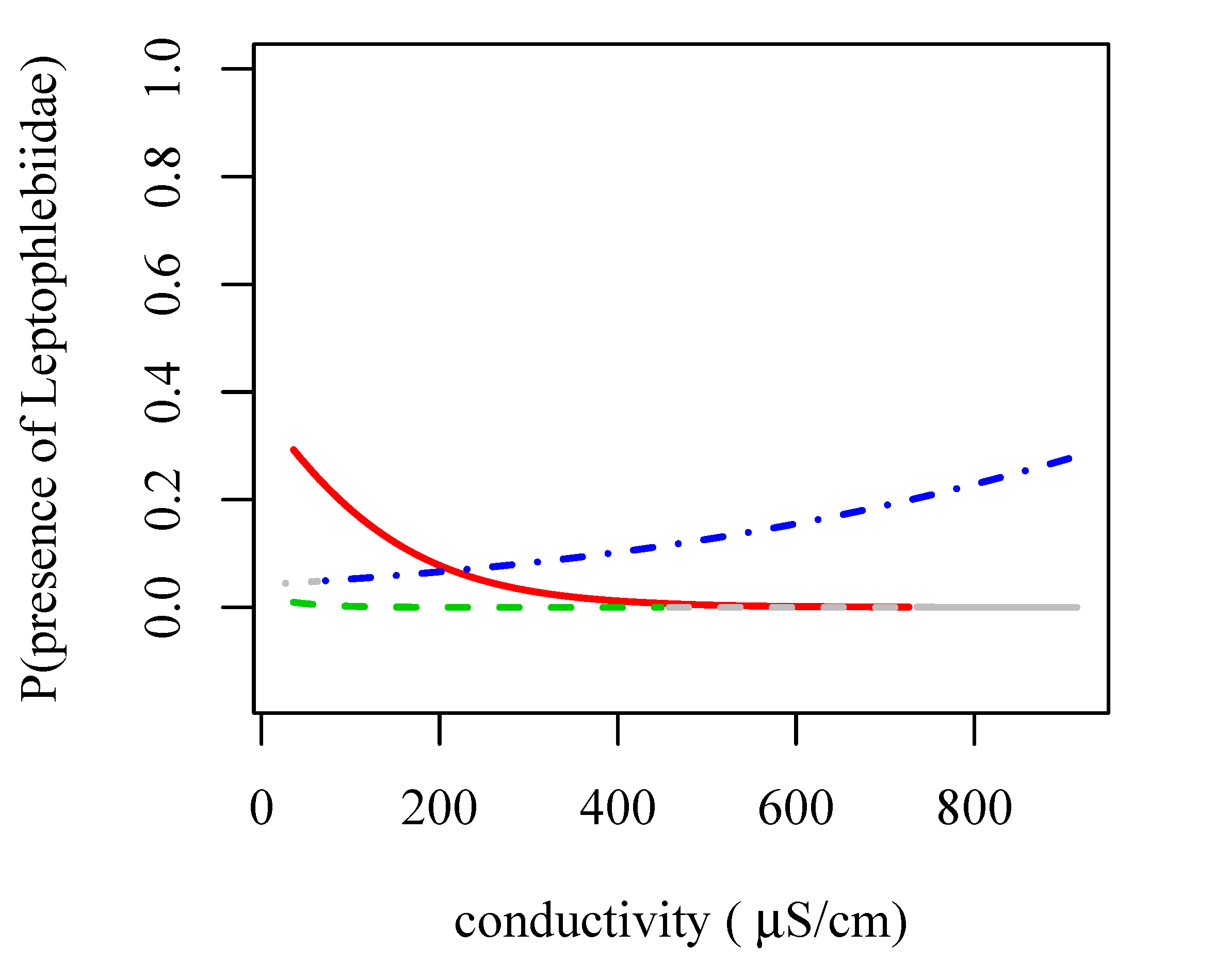

Supplement: Figure S56 — The probability of Leptophlebiidae being present in relation to the conductivity measured in Ecuador (red, solid), Ethiopia (green, dashed) and Vietnam (blue, dotdashed). The gray-colored ends of the response curves indicate extrapolation outside the observed physical-chemical range in the corresponding river basin. (DOCX) [file pone.0108898.s056.docx]

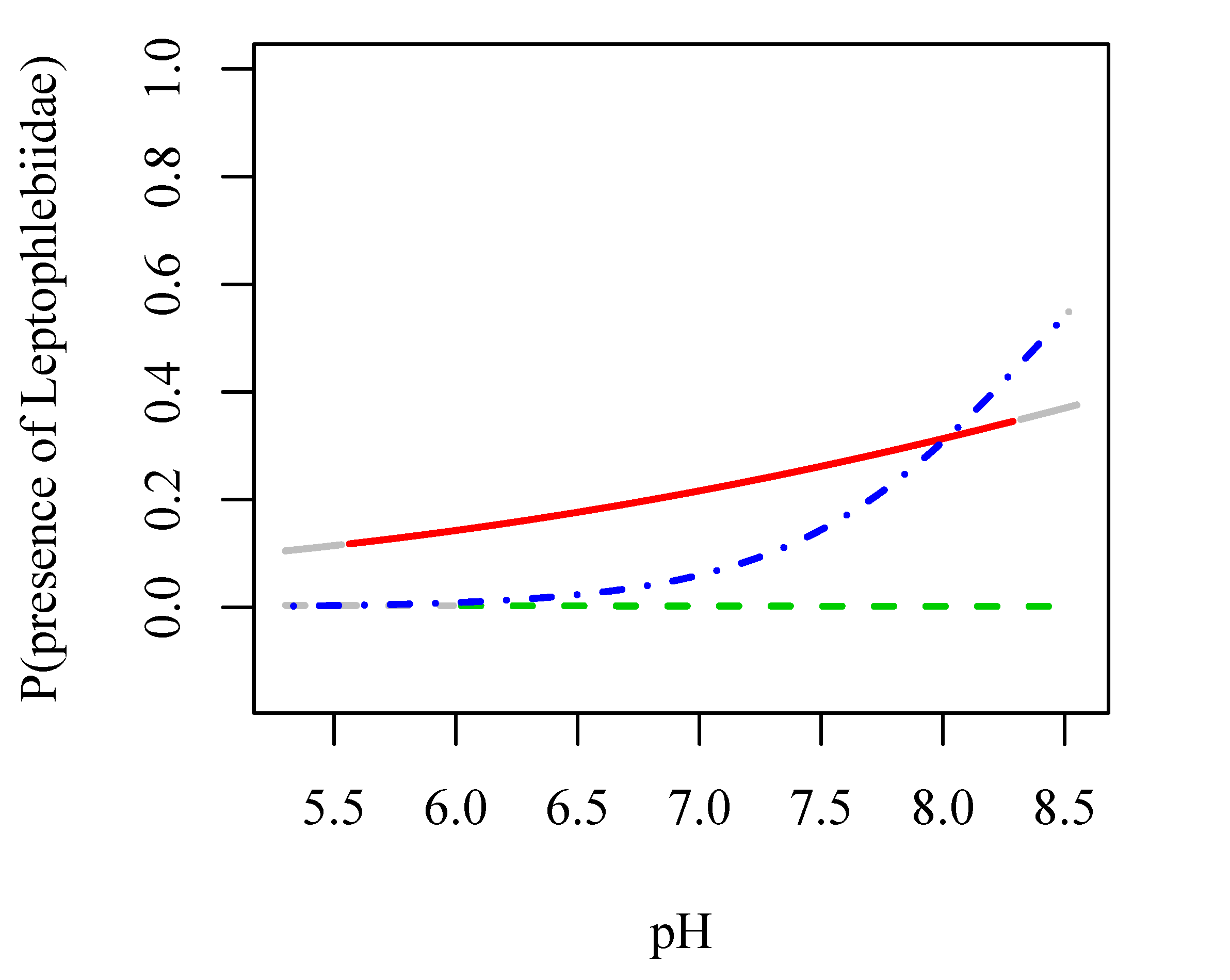

Supplement: Figure S57 — The probability of Leptophlebiidae being present in relation to pH measured in Ecuador (red, solid), Ethiopia (green, dashed) and Vietnam (blue, dotdashed). The gray-colored ends of the response curves indicate extrapolation outside the observed physical-chemical range in the corresponding river basin. (DOCX) [file pone.0108898.s057.docx]

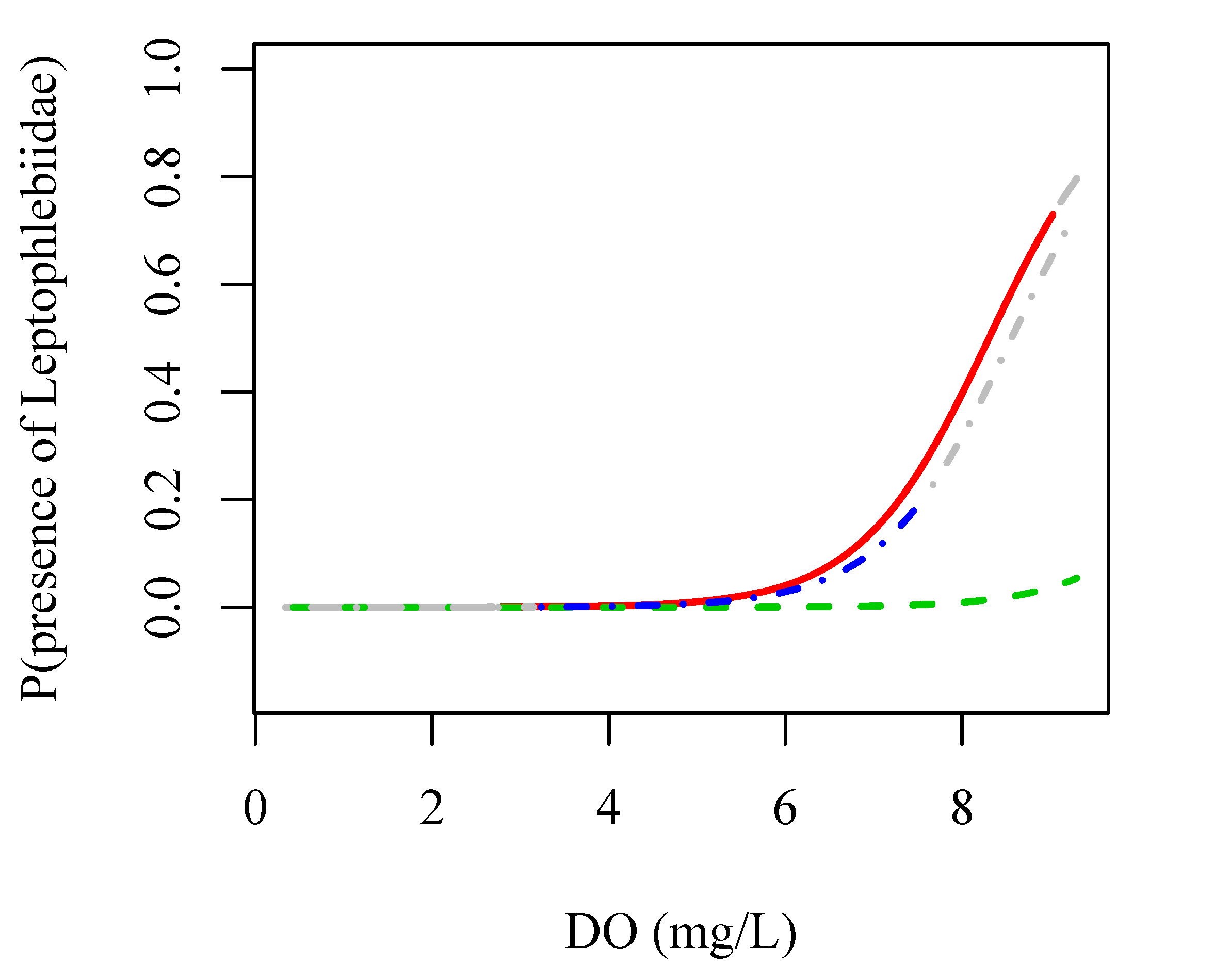

Supplement: Figure S58 — The probability of Leptophlebiidae being present in relation to dissolved oxygen (DO) concentration measured in Ecuador (red, solid), Ethiopia (green, dashed) and Vietnam (blue, dotdashed). The gray-colored ends of the response curves indicate extrapolation outside the observed physical-chemical range in the corresponding river basin. (DOCX) [file pone.0108898.s058.docx]
