# Supplementary material for: Comparison of the Abiotic Preferences of Macroinvertebrates in Tropical River Basins
Source: PLoS One. 2014 Oct 3;9(10):e108898. doi: 10.1371/journal.pone.0108898 (PMC4184827; doi:10.1371/journal.pone.0108898)
Supplement: Table S1 — Seasonal differences per physical-chemical variable and per country. A p-value less than 0.05 demonstrates a significant difference between seasons for the mean physical-chemical variable that is considered for a specific country. Significant relations are indicated in bold. (DOCX) [file pone.0108898.s059.docx]

**Table S1** Seasonal differences per physical-chemical variable and per country. A p-value less than 0.05 demonstrates a significant difference between seasons for the mean physical-chemical variable that is considered for a specific country. Significant relations are indicated in bold.

|  | **Ecuador** | **Ethiopia** | **Vietnam** |
| --- | --- | --- | --- |
| Stream velocity | **< 0.001** | **< 0.001** | **< 0.001** |
| Water temperature | **< 0.001** | **< 0.001** | **< 0.001** |
| pH | **< 0.001** | 0.598 | **< 0.001** |
| Conductivity | **0.006** | **< 0.001** | 0.385 |
| DO concentration | **< 0.001** | **< 0.001** | 0.562 |
