# Supplementary material for: Comparison of the Abiotic Preferences of Macroinvertebrates in Tropical River Basins
Source: PLoS One. 2014 Oct 3;9(10):e108898. doi: 10.1371/journal.pone.0108898 (PMC4184827; doi:10.1371/journal.pone.0108898)
Supplement: Table S2 — Country-wise differences per physical-chemical variable and per season. A p-value less than 0.05 demonstrates a significant difference between countries for the mean physical-chemical variable that is considered for a specific season. Significant relations are indicated in bold. (DOCX) [file pone.0108898.s060.docx]

**Table S2** Country-wise differences per physical-chemical variable and per season. A p-value less than 0.05 demonstrates a significant difference between countries for the mean physical-chemical variable that is considered for a specific season. Significant relations are indicated in bold.

|  | **Dry** | **Wet** |
| --- | --- | --- |
| Stream velocity | 0.322 | 0.780 |
| Water temperature | **< 0.001** | **< 0.001** |
| pH | **< 0.001** | **< 0.001** |
| Conductivity | **0.011** | **0.004** |
| DO concentration | **< 0.001** | 0.162 |
