# Supplementary material for: Comparison of the Abiotic Preferences of Macroinvertebrates in Tropical River Basins
Source: PLoS One. 2014 Oct 3;9(10):e108898. doi: 10.1371/journal.pone.0108898 (PMC4184827; doi:10.1371/journal.pone.0108898)
Supplement: Supplementary file 4 [file pone.0108898.s003.docx]

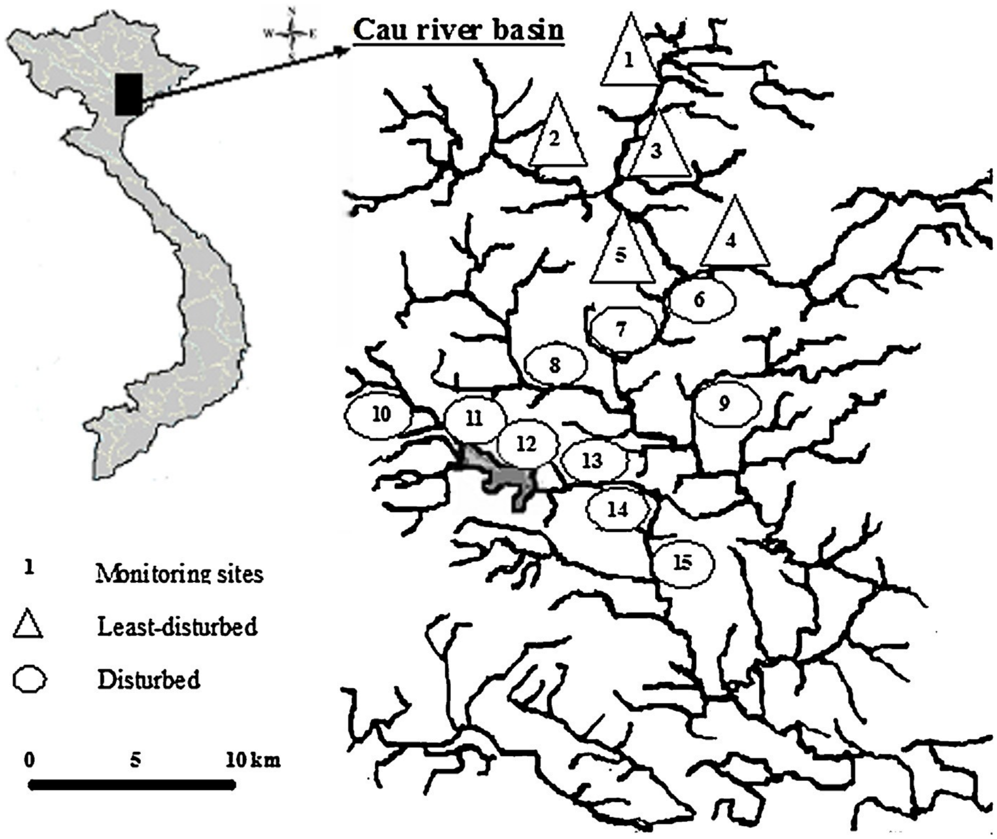

Cau river basin
1
2
3
4
5
6
7
8
9
10
11
12
13
14
15
1 Monitoring sites
Least-disturbed
Disturbed
0 5 10 km
